# Global patterns of commodity-driven deforestation and associated carbon emissions

**DOI:** 10.1038/s43016-026-01305-4

**Published:** 2026-02-23

**Authors:** Chandrakant Singh, U. Martin Persson

**Affiliations:** Department of Space, Earth and Environment, Chalmers University of Technology, Gothenburg, Sweden

**Keywords:** Environmental impact, Forestry, Forest ecology

## Abstract

Rapid agriculture-driven deforestation poses considerable challenges to achieving climate and biodiversity targets. However, the limited scope and comprehensiveness of the datasets available for linking deforestation to food production restrict their effectiveness in supporting forest conservation and climate change mitigation efforts. By integrating the best available spatial and statistical datasets, our deforestation attribution framework (DeDuCE) provides a detailed quantification of deforestation associated with the production of agricultural and forestry commodities. DeDuCE reports 9,332 unique country–commodity deforestation–carbon footprints across 179 countries and 184 commodities annually from 2001 to 2022. Our findings indicate that while global efforts to curb deforestation appropriately focus on cattle meat, oil palm, rubber, soya, cocoa and coffee, global monitoring efforts have largely overlooked staple crops such as rice, maize and cassava. Given their substantial contribution to deforestation and carbon emissions, balancing food security with forest and climate conservation will require greater attention to these crops.

## Abstract (web summary)

By integrating the best available spatial and statistical datasets, this analysis estimates the deforestation associated with the production of 184 agricultural and forestry commodities across 179 countries from 2001 to 2022.

## Main

Forests are essential to livelihoods globally, providing us with food, shelter and a habitable climate. They also supply raw materials for everyday necessities such as paper, medicine, detergents and cosmetics. However, the rising food demand of an ever-growing global population has led to extensive deforestation, with over 90% of global deforestation linked to agriculture^1,2^. A recent Food and Agriculture Organization (FAO) report^1^ suggests that over the past three decades, the world has lost an area of forest more than the size of India^3,4^. When natural forests are cleared for agricultural production, they are replaced by land systems that often lack the biodiversity and carbon storage capacity of the natural forests. Presently, deforestation is estimated to be the largest driver of biodiversity loss on land^5^, contributing nearly one-tenth of total anthropogenic greenhouse gas (GHG) emissions^6,7^, while agricultural deforestation and other land-use change activities accounting for one-third of total food system emissions^8^. These impacts from global food production raise alarming concerns about future food security, and about the suitability and sustainability of our living environments^9–11^.

Recognizing these impacts, governments, companies and civil societies have pushed for forest conservation and climate change mitigation initiatives such as the Reducing Emissions from Deforestation and Forest Degradation^12^ (REDD+), the New York Declaration on Forests^13^ and corporate Zero Deforestation Commitments^14^. These initiatives aim to engage public and private sectors in combating deforestation, incentivizing conservation and promoting deforestation-free supply chains. Notably, the recently adopted European Union Deforestation Regulation (EUDR)^15^ mandates companies to conduct due-diligence reporting to ensure the European Union’s supply chains are deforestation-free.

A key to the successful implementation and evaluation of these policy initiatives is the ability to comprehensively monitor agricultural deforestation and its climate impact^2^. However, while spatial datasets linking food production to deforestation exist for some commodities, they are often geographically limited and do not provide a comprehensive view of global food system impacts^16–18^. Conversely, national and subnational agricultural statistics offer extensive coverage of commodity production but lack the spatial precision required for linking food systems to deforestation^19^ (Supplementary Note 1). As a result, existing global or regional deforestation attribution data are primarily built on bookkeeping models^8,19,20^, with limited integration of remote sensing datasets^18,21,22^. This limited use of remotely sensed data can primarily be attributed to computational challenges in handling and processing large data volumes^23^. Consequently, datasets that do integrate remote sensing often lack ongoing updates or refinements postpublication and tend to aggregate data over lengthy periods^18,21,22,24^, diminishing their relevance over time.

With the growing trend among organizations to adopt more advanced and innovative approaches to forest resource and risk assessments^25,26^, shifting the paradigm from traditional statistical methods requires the integration of remote-sensing datasets and the utilization of powerful cloud-computing resources^27^. Such integration is imperative for stakeholders to adapt to the rapidly evolving food systems landscape and make informed decisions that balance growing food demand with forest conservation. To assist with this, we introduce the Deforestation Driver and Carbon Emissions (DeDuCE) model, which leverages the computational power of Google Earth Engine (GEE) to meld the spatiotemporal precision of the best available remote sensing data and the comprehensiveness of agricultural statistics. The model tracks deforestation and associated carbon emissions, and links them with the production of agriculture and forestry commodities globally.

### State-of-the-art of the model

**Extended Data Fig. 1:**
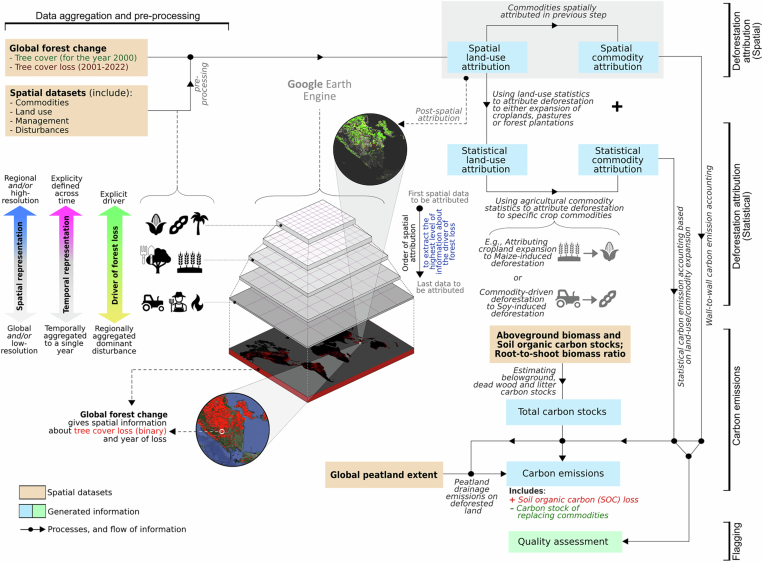
Framework for the Deforestation Driver and Carbon Emission (DeDuCE) model. This framework consists of three key components: deforestation attribution (spatial and statistical), carbon emission calculation, and quality assessment. In the first step, we utilise remote sensing and (sub-) national agricultural statistics to determine what portion of the total annual tree cover loss is attributable to specific commodities. From this, we next calculate carbon emissions linked to commodity-driven deforestation, including emissions from peatland drainage on deforested lands. Finally, we evaluate the reliability of our deforestation estimates by assessing the quality of the input data used in our analysis. A detailed description of the datasets used in this model is provided in Supplementary Table 2.

The DeDuCE model provides annual estimates of deforestation and associated carbon emissions due to the production of agricultural and forestry commodities. In this study, the term ‘commodity’ is used broadly to encompass all agricultural and forestry products, irrespective of whether it is intended for market trade or subsistence use. Covering 179 countries and 184 commodities between 2001 and 2022, the model delivers 9,332 unique deforestation–carbon footprint estimations (Supplementary Table 1). The model achieves this comprehensive deforestation attribution by overlaying global spatiotemporal data of tree cover loss^28^ with best available datasets on crops, land uses, dominant deforestation drivers^24^ and state of forest management (Extended Data Fig. 1 and Supplementary Table 2). Each tree-cover-loss pixel is linked to the most detailed information available about the direct land-use change (dLUC)^29,30^ (that is, a specific commodity or land use).

In cases where deforestation cannot be spatially attributed to a specific commodity, the model uses agricultural statistics (at national and subnational levels^3,31^) to identify likely or potential drivers of deforestation. This reflects statistical land-use change (sLUC), a measure of deforestation risk, and is implemented through a two-step statistical land-balance approach^19^ (Supplementary Fig. 1). Through this, the model accounts for key land-use change dynamics, such as competition between cropland, pasture and other land uses, and includes cropland and pasture abandonment. These factors are crucial for attributing deforestation to agricultural commodity production, but are poorly captured in existing life-cycle inventory databases^32^. Additionally, carbon emissions associated with deforestation are estimated by overlaying identified deforestation drivers with data on forest^33^ and soil^34^ carbon stocks, including emissions from peatland^35^ drainage (Extended Data Fig. 1).

By combining GEE’s computational capabilities to process terabytes of high-resolution spatiotemporal data with Python’s open-source programming for deforestation–emission accounting, our model aligns with FAIR data principles^36^, striving to promote accessibility, integrity and transparency. This integration also ensures replicability of model results, while fostering community engagement, inviting researchers and stakeholders to contribute and refine the model (Supplementary Note 2). Such engagements are crucial because growing food demand greatly influences regional and remote landscapes due to different environmental, technological, regulatory and socioeconomic factors^37–40^.

Presently, there is a lack of clear and explicit guidelines regarding data and methods for deforestation–emission accounting^41,42^, leading to inconsistent outcomes across its use cases (Supplementary Note 3). Although such discrepancies are understandable given our limited capacity to model the complex and dynamic nature of global food systems, the DeDuCE model addresses these challenges by providing a versatile and globally homogeneous framework for attributing deforestation and carbon emissions to commodities.

While combining extensive spatial and statistical data within a globally consistent modelling framework is a strength of the DeDuCE model, it also presents a limitation: differences in the scale, scope (for example, dLUC and sLUC), and quality of input data across space and time mean that users must be cautious when comparing results from different regions and commodities. To communicate this clearly, we provide an Integrated Quality Index (IQI) for each data point, which considers both quantitative and qualitative assessments of the spatial and statistical input data to provide an indication of confidence—although not a direct measure of accuracy—in the resulting deforestation estimates. This enhances the model’s utility as a tool for supporting global sustainability and conservation efforts by highlighting the variation in the robustness of model results across regions, countries, and commodities (Supplementary Notes 4 and 5 and Supplementary Table 3). It also aids in identifying countries and commodities where better data are needed to improve estimates of commodity-driven deforestation (see Results subsection ‘Quality assessment and potential for model improvement’).

Furthermore, the model’s versatility allows for the inclusion of diverse datasets (Supplementary Table 2) and is designed to integrate emerging datasets, ensuring its relevance and adaptability over time. This makes it possible to adjust parameters such as tree cover density for forest classification, lag periods between forest clearing and agricultural land establishment, control over attribution methodology, and amortization periods, as per the required use case.

## Results

### Global overview of deforestation and carbon emissions

**Fig. 1:**
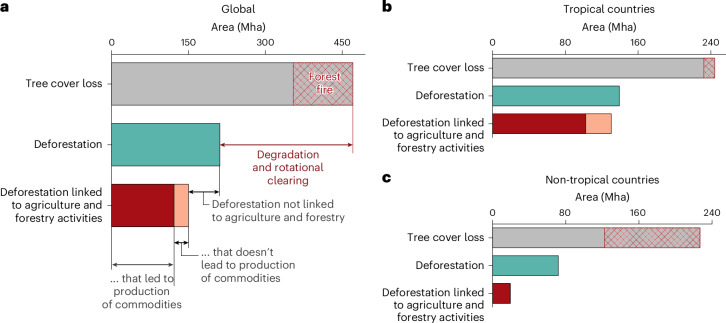
Deforestation linked to agriculture and forestry from global tree cover loss estimates (2001–2022). **a**, Bar plot illustrating deforestation driven by agriculture and forestry activities derived from global tree cover loss estimates (refers to loss of tree canopy within a 30-m-pixel globally between 2001 and 2022^28^; tree cover density ≥25%). The difference between tree cover loss and deforestation (that is, loss of natural forests associated with deliberate clearing of forest for commodity production) is broadly referred to as forest degradation and rotational clearing. This includes tree cover loss on managed or degraded lands established before the year 2000 (for example, rotational clearing in managed forests and plantations or loss of sparse growth on degraded land systems), but excludes the conversion of natural forests into forest plantations, which is counted as deforestation. Within this, loss of natural forests due to fires, post-year 2000, that are not followed by the establishment of commodity-driven land use is indicated with hatch patterns. Additionally, the scope of deforestation driven by agriculture and forestry activities extends to include the instances where it can be directly linked to the production of these commodities, and where it occurs independently of such production. The latter scenario is examined by evaluating the extent of this deforestation that cannot be linked to any specific commodity in the DeDuCE’s modelling framework (Extended Data Fig. 1). However, in this calculation, deforestation linked to pasture does not account for variations in cattle stocking rates or pasture productivity, which could indicate clearing for speculation (rather than production). Possible mechanisms where deforestation does not lead to the production of commodities are discussed in ref. ^2^. **b**,**c**, To offer a comparative insight into deforestation dynamics across different biomes, we have also separated our analysis for tropical (**b**) and non-tropical (**c**) countries. Source data

**Fig. 2:**
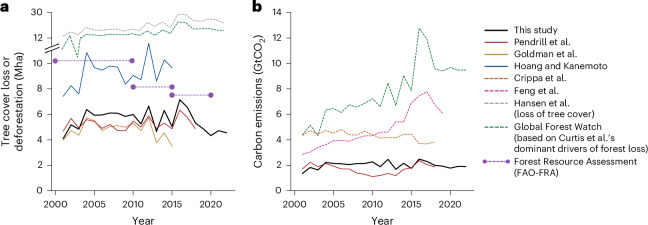
Comparison of different commodity-driven deforestation and carbon emission estimates. **a**,**b**, A comparison between our deforestation estimates (**a**) and associated carbon emission estimates (**b**) with those from established literature sources. The comparison includes estimates from Pendrill et al.^49^ (covering only tropical counties), Goldman et al.^18^ (covering only EUDR commodities), Hoang and Kanemoto^21^, Crippa et al.^8^ (including all food-production-driven land-use change activities), Feng et al.^22^ (accounting for tree cover loss due to agriculture and forestry activities across the tropics), Hansen et al.^28^ (tree cover ≥25%), Global Forest Watch^90^ (tree cover ≥25%; including only tree cover loss due to commodity-driven deforestation, shifting agriculture and forestry from Curtis et al.^24^), and the FAO’s global forest resource assessment report (FAO-FRA; loss of naturally regenerating forest)^3^. A brief summary of the studies and datasets used for this comparison can be found in Supplementary Table 4. Source data

The DeDuCE model suggests that of the 471 Mha of global tree cover loss observed between 2001 and 2022, only 26% (5.5 ± 0.8 Mha yr^−1^) is driven by expansion of croplands, pastures and forest plantations for commodity production (Fig. 1a). This estimate is considerably smaller than the FAO’s reported deforestation estimate of 8–10 Mha yr^−1^, even with the consideration that only 90% of the FAO’s reported deforestation is attributed to agriculture^1^ (though the discrepancy is notably smaller in more recent years; Fig. 2a). In comparison, Curtis et al.^24,43^ estimate that 44–76% of global tree cover loss is attributed to agriculture and forestry activities. This discrepancy occurs because Curtis et al.^24^ overlook spatiotemporal heterogeneity—by attributing only the dominant forest-loss driver over the whole timeframe—and finer land-use change dynamics (for example, rotational clearing) (Fig. 1). Furthermore, the share of commodity-driven deforestation from DeDuCE exhibits stark contrasts between tropical and non-tropical regions: 42% of the tree cover loss in tropical countries is attributed to production associated with agriculture and forest plantations, compared to just 9% in non-tropical countries (Fig. 1b,c).

Compared to previous assessments^2^, DeDuCE presents a lower overall estimate of deforestation due to agriculture and forestry activities, yet it shows marginally higher figures for deforestation leading to production (Fig. 1b). Notably, Pendrill et al.^2^ estimated that as much as a third to half of agriculture-driven deforestation did not result in any identifiable agricultural production. In contrast, our analysis puts this number much lower, at just over a fifth (25 Mha from a total of 130 Mha agricultural-driven deforestation; Fig. 1b). This improved understanding about the role of commodity production in driving deforestation is due to our use of high-resolution agricultural land-use maps, reducing reliance on coarse dominant forest-loss driver data and poor-quality agricultural statistics. Additionally, our integration of forest fire data^44^ and the sequential attribution framework of the DeDuCE model (that is, attributing forest-loss pixels to agricultural land use before attributing forest loss to fire; Methods) enables us to distinguish wildfires, often propagating in grass-dominated natural and seminatural landscapes^45^, from fires used to clear land for agricultural expansion. The remaining discrepancies between agriculture-driven deforestation and productive use of the cleared land in the tropics—which still are substantial—probably reflect challenges in land tenure clarity and disputes^2^. For instance, speculative clearing anticipating future agricultural returns, planned infrastructural developments, uncertain future forest conservation legislations and availability of large expanses of undesignated public lands may fail to evolve into productive agricultural or forestry ventures^46,47^.

**Fig. 3:**
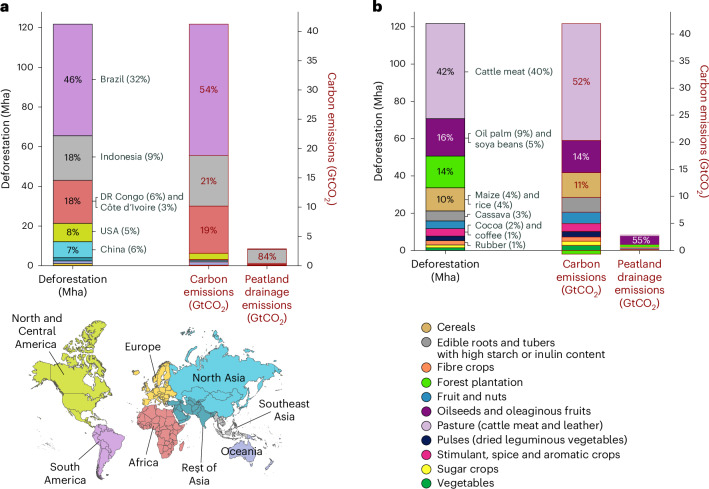
Global overview of commodity-driven deforestation and carbon emissions from DeDuCE (2001–2022). **a**,**b**, Deforestation is attributed to agriculture and forestry commodities, and the corresponding carbon emissions globally, categorized by geographical regions (**a**) and commodity groups (**b**). The left bar in each panel shows deforestation by area; the right bars present associated carbon emissions. Emissions from peatland drainage linked to deforestation are shown separately as the rightmost bar plot in each panel. Between 2001 and 2022, total deforestation associated with the production of agricultural and forestry commodities amounted to 121.8 Mha, resulting in 41.2 GtCO_2_ of carbon emissions (equation (1)), plus an additional 2.9 GtCO_2_ through peatland drainage. The relative contributions of geographical regions, commodity groups, and selected major deforestation countries and commodities are also expressed as percentages of total deforestation and carbon emissions. Furthermore, the contribution of commodities, broken down by geographical regions, is illustrated in Supplementary Fig. 2. Country- and commodity-specific estimates are available at https://www.deforestationfootprint.earth. Source data

We estimate nearly 41.2 GtCO_2_ emissions from commodity-driven deforestation globally from 2001 to 2022 (1.9 ± 0.3 GtCO_2_ yr^−1^). Additionally, emissions from peatland drainage on land deforested since 2001 contribute to approximately 2.9 GtCO_2_ (0.13 ± 0.08 GtCO_2_ yr^−1^; Figs. 2b and 3), accounting for about 7% of global annual peatland drainage emissions^48^. Our carbon emission estimates are substantially lower than previously reported (Fig. 2b), except for those of Pendrill et al.^49^, who only cover the tropics. Crippa et al.^8^, using FAOSTAT data^31^, estimate agricultural land-use emissions (including those from deforestation) at 4.3 ± 0.3 GtCO_2_ yr^−1^, which is twice our estimate (excluding deforestation emissions from forestry activities from Fig. 2b). Because forests hold the majority of carbon stocks, agricultural land-use changes other than deforestation are unlikely to account for the remaining land-use change emissions. The probable reason for this discrepancy is that Crippa et al.’s^8^ estimates do not utilize spatial information on deforestation, agricultural land use or carbon stocks, but simply assume that 80% of all deforestation is due to agricultural land-use change. This underscores the value of utilizing remote-sensing-based data to assess agriculture-driven deforestation and associated carbon emissions.

**Fig. 4:**
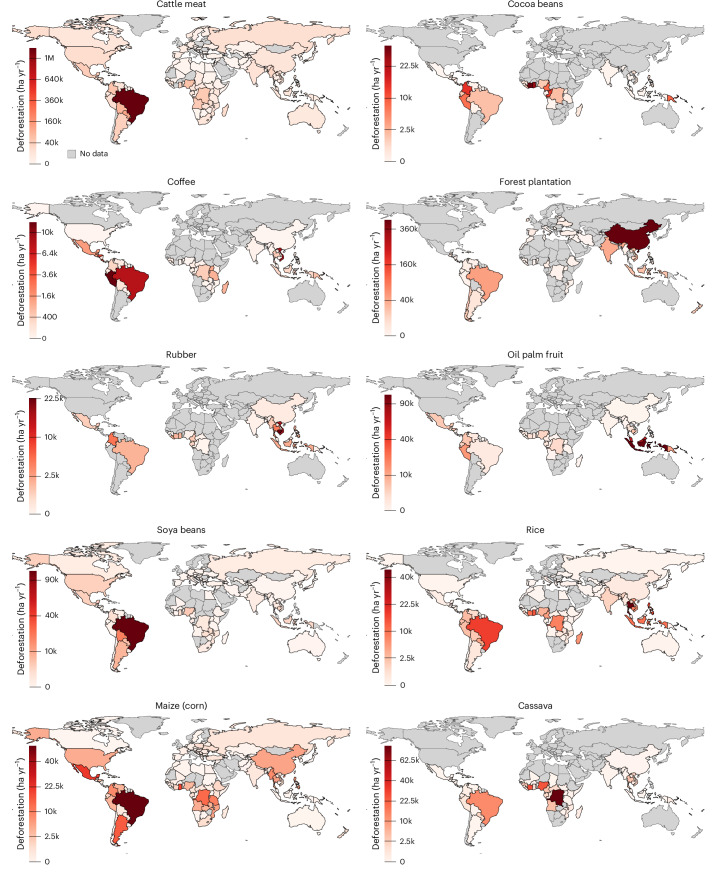
Spatial patterns of major deforestation-risk commodities (aggregated for 2018–2022). This figure illustrates the spatial distribution of deforestation-risk commodities regulated under the EUDR, along with major staple crops. The deforestation estimates are averaged over the recent five years (2018–2022) and represented in ha yr^−1^. The IQI for these commodities is detailed in Supplementary Fig. 4. Deforestation-risk regions for the commodities (shown above) in Brazil at the municipality-level are illustrated in Supplementary Fig. 5. Source data

Our analysis also reveals an uneven distribution of both deforestation and the resulting carbon emissions across regions and commodities (Fig. 3): between 2001 and 2022, South America leads in both, with Southeast Asia and Africa also showing major contributions. Together, these three regions account for roughly 82% of global deforestation and 94% of carbon emissions due to expanding agriculture and forest plantations. Additionally, during this period, deforestation in Southeast Asia alone is responsible for nearly 84% of global peatland drainage emissions (Fig. 3a). Nevertheless, two countries outside the tropics—China and the United States—closely trail the top three countries globally—Brazil, Indonesia and the Democratic Republic of Congo (DR Congo)—in terms of deforestation area, although not in carbon emissions (Fig. 3a). We suspect that the low deforestation estimates associated with forest plantations in boreal regions (Fig. 4) may be due to datasets inadequately capturing the conversion of natural forests and the absence of a primary forest mask^50^, probably leading to their underestimation in our estimates.

In terms of specific commodity groups, over the period 2001–2022, deforestation driven by pasture expansion (primarily for cattle meat production) represents about 42% of total deforestation and 52% of the carbon emissions (Figs. 3b and 4). This is followed by the cultivation of oilseeds and oleaginous fruits, especially oil palm and soybeans, which account for 16% of total deforestation and 14% of carbon emissions. Notably, oil-palm-induced deforestation, primarily in Southeast Asia, alone accounts for nearly 55% of peatland emissions (Figs. 3b and 4). Other major contributors to deforestation include forest plantations (14%), stimulant and aromatic crops (3%, largely driven by cocoa beans and coffee cultivation) and fibre crops (2%, mostly rubber) (Fig. 3b).

Although these commodities are included in the EUDR^15^ due to their high deforestation and trade shares, our analysis also reveals that staple crops—specifically maize, rice and cassava—cumulatively account for about 11% of total deforestation (Fig. 3b), exceeding that of cocoa, coffee and rubber. Unlike other commodities, whose production and deforestation are concentrated in specific regions (for example, oil palm in Southeast Asia, soybeans in South America), the deforestation associated with staple crops is globally distributed (Fig. 4). Moreover, given that nearly half of the global average human diet consists of staple commodities^51^, and their cultivation is expected to increase to feed the growing population^52^, incorporating staple crops into deforestation monitoring and regulatory frameworks will be vital for curbing global deforestation, promoting sustainable agricultural supply chains and ensuring future food security.

When comparing our estimates for major deforestation-risk agricultural commodities with other datasets (Supplementary Note 5, Supplementary Table 3 and Supplementary Fig. 3), we find that while trends for certain commodities, such as cocoa beans in Côte d’Ivoire and Ghana, oil palm in Indonesia, and pasture in Brazil, are consistent across different datasets, considerable differences arise for other major forest-risk commodities. Although these discrepancies are less pronounced at the global or pantropical level, they become quite stark at the individual country–commodity level (Supplementary Table 3 and Supplementary Fig. 3). However, compared to the only previously existing pantropical, commodity-specific deforestation driver dataset^2^, the DeDuCE estimates align much more closely with high-resolution remote-sensing studies. Where larger discrepancies exist, they are primarily due to differences in the underlying forest, deforestation and commodity datasets (Supplementary Note 5).

### Quality assessment and potential for model improvement

**Fig. 5:**
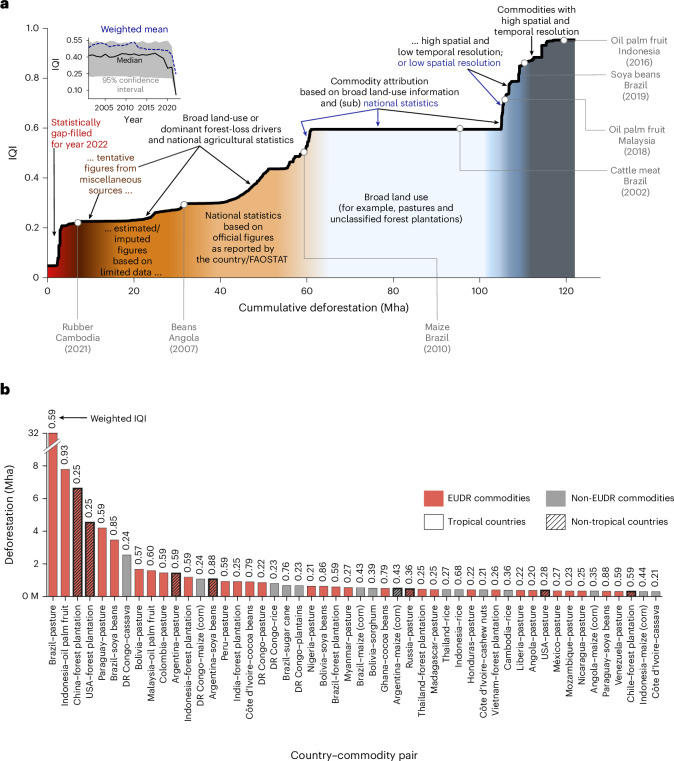
Quality of commodity-driven deforestation estimates (2001–2022). **a**, The ranked line plot visualizes the IQI of all deforestation estimates for different country–commodity pairs, arranged from the lowest IQI score (on the left) to the highest (on the right) between 2001 and 2022. The labels provide insights into the dominant data types and their level of explicitness, which contribute to the respective IQI rankings. Inset: temporal IQI subplot in which the 95% confidence interval represents the 2.5th and 97.5th percentiles of the IQI values. **b**, To highlight the quality of data currently used for deforestation attribution (2001–2022), we present the top-50 deforestation-risk country–commodity pairs along with their respective weighted average IQI. These top-50 country–commodity pairs account for approximately 70% of global deforestation. Source data

The IQI, based on the spatiotemporal granularity, explicitness and the accuracy of the spatial and statistical datasets used as inputs in the DeDuCE model, indicates the reliability of the resulting direct deforestation estimates (‘Quality assessment’). Only 12–15% of attributed deforestation in DeDuCE is derived from spatial commodity-specific datasets, representing dLUC (IQI ≥ 0.6; Fig. 5a). In contrast, 30–35% of the attribution uses broad spatial land-use information (for example, the extent of pastures), mainly attributing deforestation to cattle meat and forest plantations (dLUC; 0.6 > IQI ≥ 0.55). The remaining 50–58% blends spatial and statistical datasets, where the resulting estimates should be interpreted as a measure of deforestation risk for a given commodity (sLUC; IQI < 0.55) (Fig. 5a). In this case, deforestation estimates derived from officially reported agricultural statistics (including subnational statistics) receive a higher score, whereas those imputed or estimated by FAOSTAT are assigned a lower score, as illustrated by the progression of FAO quality flags in Fig. 5a.

Despite using the best available datasets, pixel- or municipality-level deforestation attribution is limited to certain commodities and countries (Supplementary Table 2). Thus, we should target areas where enhancements will considerably boost the quality of deforestation estimates. Examining the IQI of the top-50 deforestation-risk country–commodity pairs (accounting for 70% of global deforestation; Fig. 5b), we find that forest plantations (in China, the United States and India) and pastures (outside South America) often receive lower IQI scores. This is probably due to the challenge of mapping pastures and forest plantations because their spectral signatures are similar to natural grasslands and forests. Additionally, staple commodities are not well represented in terms of data quality, even though several countries have substantial deforestation associated with these commodities (Fig. 5b). Furthermore, due to poor-quality spatial data and agricultural statistics, African countries show consistently lower-quality deforestation estimates, which include commodities such as cassava, maize, rice, beans and cocoa (Fig. 5b).

Consequently, global deforestation attribution could be greatly improved by incorporating global maps of (1) pastures, (2) forest plantations and (3) cereals (primarily for maize and rice), and by (4) improving spatial representation of agricultural commodities contributing to deforestation in Africa (particularly in DR Congo and Nigeria). Existing initiatives such as Global Pasture Watch^53^, the Spatial Database of Planted Trees^54^ (SDPT), the WorldCereal database^55^ and the Global Subnational Agricultural Production^56^ (GSAP) database could provide critical data to help close these gaps in the near future.

### Influence of modelling assumptions on deforestation and carbon emission estimates

To assess the robustness of the DeDuCE model, we examined the sensitivity of deforestation and carbon emission estimates to various modelling assumptions (Table 1). The most notable changes were observed when we ran the model solely or primarily as a statistical deforestation attribution model, using the global forest change^28^ (GFC) data only (similar to ref. ^49^) or together with data on dominant forest-loss drivers^24^ (similar to refs. ^21,22^). In these cases, deforestation and carbon emission estimates were inflated by 40–85% compared with our current estimates (Table 1), explaining the discrepancy with Crippa et al.^8^. This inflation occurs because these attribution methodologies use datasets that overlook spatiotemporal heterogeneities or equate all tree cover loss with deforestation.

**Table 1:** Sensitivity of deforestation and carbon emission estimates to modelling parameters

Broad category	Sensitivity control	Sub-category	% change from reference	
			Deforestation	Carbon emissions
**Forest and deforestation**	Tree cover density^28^	≥10%	6.59	1.42
		≥75%	−29.98	−10.97
	JRC Global forest cover 2020^57^ (only compared with deforestation estimates from 2020 to 2022)	—	−11.15	−39.72
	JRC TMF Deforestation^59,60^ (only compared for TMF countries)	—	−28.17	−18.84
**Forest plantation**	Assuming that all plantations from SDPT^54^ were established before the year 2000	All commodities	−0.03	<0.01
		Only forest plantations	−0.17	−0.41
**Attribution lag period from the year of deforestation**	Spatial lag period (only compared for the MapBiomas countries)	1 year	−9.95	−8.97
		5 years	4.25	3.82
	Statistical lag period	1 year	−0.72	−0.84
		5 years	0.09	0.54
**Inclusion of spatial datasets**	Partial statistical attribution (only using Global forest change^28^, the dominant driver of forest loss^24^ dataset and agricultural statistics^31^)	All commodities—global	40.53	39.48
		Oil palm–Indonesia	19.72	38.82
		Cocoa—Côte d’Ivoire	−29.21	−42.56
		Soya beans—Brazil	234.81	373.56
	Full statistical attribution (only using the Global forest change^28^ dataset and agricultural statistics^31^)	All commodities—global	86.00	73.12
		Oil palm—Indonesia	20.27	36.19
		Cocoa—Côte d’Ivoire	−28.87	−42.16
		Soya beans—Brazil	151.83	246.03
**Land-use expansion**	Croplands do not expand over pastures first, but expand over forests directly	—	0.22	0.31
	Net agricultural expansion	—	−7.63	−9.20
	All statistical land-use attribution is restricted by FAOSTAT	—	−1.23	−1.65
	All statistical land-use attribution not restricted by FAOSTAT	—	20.78	28.08
**Agriculture statistics (only for Brazil)**	National-level agricultural statistics	—	0.12	0.10
**Multiple cropping** **(only for Brazil)**	Not accounting for the harvested area from multiple cropping	Maize	35.38	35.83
		Beans	19.56	29.11
		Potatoes	47.63	39.65
		Groundnuts	−7.13	−9.16
**Accumulation period of peatland drainage emissions**	Global (compared with accumulation until the year 2022 from the year of deforestation)	No accumulation	—	−91.39
		5 years from the year of deforestation	—	−58.91
		10 years from the year of deforestation	—	−26.09
		15 years from the year of deforestation	—	−7.07
**Net carbon emissions (excluding peatland drainage emissions)**	Only considering 75% of the mature plant carbon stocks for the replacing commodity (equation (1))	All commodities	—	8.16
		All tree crops	—	16.46
		Oil palm	—	16.74

The absolute reference and sensitivity analysis values are provided in Supplementary Table 5. The deforestation attribution and carbon emission estimates from all sensitivity analyses are available from Zenodo^88^.

Another notable source of uncertainty concerns forest and deforestation definitions: changing tree-cover thresholds or baseline forest maps changed deforestation estimates by as much as −30% to +7% (Table 1). Notably, using the EU Joint Research Centre’s (JRC’s) recent forest cover map^57^ resulted in a 12% reduction in deforestation estimates. Although this map closely aligns with the FAO’s forest definition^3^ and excludes agriculture and forest plantations—despite its flaws^58^—its 2020 base year makes it unsuitable for our 2001–2022 deforestation attribution. Using JRC’s tropical moist forest (TMF) deforestation data^59,60^ led to a reduction in estimated deforestation by nearly 30% in the countries it covers. The core reason lies in methodological differences: GFC detects the first tree-cover loss event annually, whereas JRC TMF only identifies deforestation when disturbances in a tree-cover pixel persist for more than 2.5 years (ref. ^61^). Additionally, JRC TMF deforestation does not account for the loss of dry forests, making its deforestation estimates more conservative.

Another important parameter influencing model estimates is the period between forest-loss detection and agricultural land establishment used for attributing deforestation. We find that a longer lag period captures more delayed land-use changes (often in the case of tree crops and forest plantations), while a shorter lag period does the opposite (Table 1). Interestingly, another major source of model uncertainty that is difficult to account for globally is multiple cropping (that is, multiple harvesting cycles on the same land). Analysing results for Brazil, we found that not accounting for multicropping increased deforestation estimates by about 20–50% for commodities with larger harvested areas (for example, maize, beans; potentially due to proportional commodity attribution in Supplementary Information, equations (9)–(12)) while reducing estimates for those with lower harvested areas (for example, groundnuts) (Table 1). Despite 12–20% of global croplands being multicropped^62^, assessing their dynamics on a global scale remains challenging due to the lack of appropriate data that capture the multiple harvest cycles of globally diverse crop combinations.

Although the sensitivity of carbon emissions closely follows the sensitivity of deforestation estimates, this is not the case for peatland drainage emissions linked to deforestation. Also, it is important to recognize that while emissions from forest biomass and soil organic carbon (SOC) are treated as inevitable due to our relatively better understanding of their carbon stocks (‘Carbon emissions calculation’), estimating the lifetime of peatland drainage emissions is challenging because these emissions can persist for several decades after deforestation. Sensitivity analysis reveals that a shorter accumulation period (5–10 years) can underestimate peatland drainage emissions by about 25–60% (Table 1 and Supplementary Fig. 6). Thus, treating peatland drainage emissions in the same manner as other committed deforestation emissions, despite their fundamentally different temporal dynamics, risks underestimating their true impact, and requires careful consideration across both space and time.

Lastly, our carbon emission estimates are probably conservative because the baseline approach does not explicitly represent average crop carbon stocks prior to harvest, which vary across regions and management systems. Instead, we deduct mature crop carbon stocks from gross emissions (equation (1)), even though average carbon accumulation by these commodities before harvest is typically lower and influenced by factors such as crop residue management, pruning intensity and rotation length in perennial cropping systems. However, assuming that crops, on average, reach 75% of their mature carbon stock before harvest, we estimate global emissions (excluding peatland drainage emissions) to be 8% higher than in our baseline reported scenario, with the largest effect observed for tree crops such as oil palm (+16%) (Table 1).

## Discussion

The DeDuCE model reinforces that food systems are the primary driver of deforestation (Figs. 1 and 3) and a major source of global carbon emissions^8^. The data produced by the model can serve as a strong evidence base for developing national GHG inventories^63^, reporting standards^30^, targeted policies^12^ and regulatory frameworks^29^. Such guidance is crucial for private- and public-sector organisations to manage and adapt their operations and value chains in line with global sustainability targets^64^.

The importance of developing food system emission inventories was highlighted at COP 28, at which nations were urged to integrate agriculture and food systems into their national climate and biodiversity plans^65^. To meet this commitment, governments must comprehensively assess their food system impacts—by estimating agricultural land-use changes and associated carbon emissions—and set targets to reduce emissions in their Nationally Determined Contributions by 2025. Shifting from broad-stroke assessments^8^ to detailed, commodity-specific deforestation and carbon emission estimates will help identify priority areas for targeted actions. Furthermore, globally consistent food system emission estimates are crucial for coordinating global action and aligning conservation and mitigation strategies^66^.

The private sector also stands to gain from globally comprehensive deforestation and carbon emission accounting. A prime example is the Science-Based Targets initiative for Forest, Land, and Agriculture (SBTi FLAG)^29^, which guides companies in setting emission reduction targets and provides independent validation of these targets against current sustainability goals. With a specific focus on deforestation due to EUDR commodities, rice, maize and wheat, among other products, companies should use the best and most complete data available per commodity and region, trailing back 20 years, to comprehensively assess their present emissions^29^—a requirement for which the DeDuCE data are highly suited. This also applies to financial institutions, which are increasingly called upon to evaluate the sustainability of their investments^67^.

**Extended Data Fig. 2:**
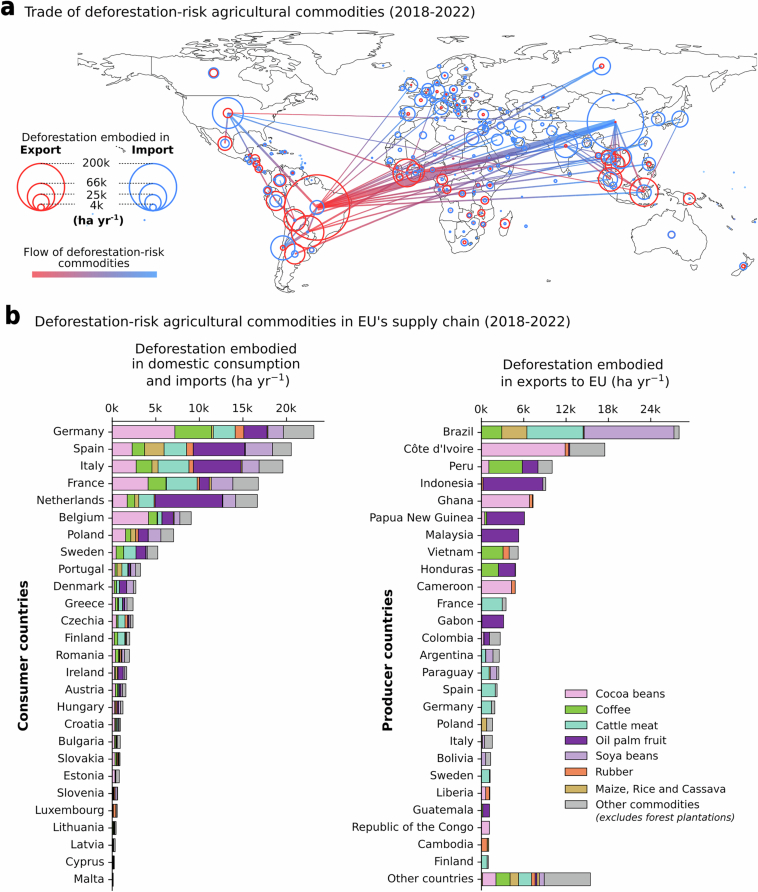
Global supply chain’s exposure to deforestation (aggregated for 2018–2022). (**a**) This figure illustrates the deforestation embodied in the trade of agricultural commodities worldwide, with exporter countries represented by red circles and importer countries by blue circles. The lines connecting these countries indicate the trade networks and the width of these lines highlights the extent of deforestation embodied in those trades. Minor trade flows, that is, less than 2% of the maximum deforestation embodied in trade, are not shown for clarity. (**b**) The figure focuses on the EU’s supply chain, showing deforestation embodied in both domestic consumption and trade. It quantifies the exposure of EU countries and their associated producer (or exporter) countries to agricultural commodities. To assess deforestation embodied in trade, we use DeDuCE’s deforestation estimates averaged over 2018–2022 with a physical trade model^68^ (see data availability), following the methodology outlined in ref.^49^.

The estimates from the DeDuCE model can also support assessments of the environmental footprint of food consumption and the deforestation exposure of global supply chains. Combining our deforestation estimates with a physical trade model^68^ (Data availability), we find that in 2022, about 30% of global agricultural deforestation was embodied in traded goods. South America and Southeast Asia are major exporting hubs for these deforestation-risk commodities, while China, the European Union, the United States, India and Japan are major importers (Extended Data Fig. 2a). Furthermore, the European Union, being the second largest trader of deforestation-risk agricultural commodities, accounts for about 14% of all globally traded deforestation-risk agricultural commodities. Major EU economies, such as Germany, Spain, Italy, France and the Netherlands, are primary importers of cocoa, coffee, oil palm, soybeans, cattle meat and maize (Extended Data Fig. 2b).

The EUDR—currently set to launch by the end of 2026^15^—requires food system actors to establish due-diligence systems that mitigate deforestation risks within supply chains^69^. These systems must reflect the deforestation-risk of exporter countries, based on a benchmarking system designed to account for rates of deforestation, agricultural expansion and commodity production^15^. However, unclear thresholds for classifying deforestation-risk benchmarks^15^ due to the lack of global-scale spatiotemporal deforestation data have posed considerable challenges for implementing the EUDR^58^. We believe that the commodity-driven deforestation estimates provided by the DeDuCE model can offer essential input for EUDR risk benchmarking.

Although the EUDR aims to promote sustainable land-use practices, many exporter countries have expressed concerns about its implications on trade due to their economic priorities, legal frameworks and the additional costs required to develop enforcement capabilities^70,71^. These factors can, in turn, increase the potential for leakages to non-EU markets^72^ (Extended Data Fig. 2a). The estimates from the DeDuCE model can be used to assess such leakages for countries committed to achieving their climate goals.

In conclusion, we believe that the versatility of the DeDuCE model, combined with the comprehensiveness of its results, which integrate the best available spatial and statistical data to provide up-to-date estimates of commodity-driven deforestation and carbon emissions, makes it ideal for a broad range of global forest conservation and climate change mitigation efforts.

## Methods

The DeDuCE model leverages a comprehensive array of spatial datasets and agricultural statistics to quantify deforestation and associated carbon emissions from agricultural and forestry activities. The modelling framework involves three primary steps (Extended Data Fig. 1), each detailed below: (1) spatial and statistical deforestation attribution associated with the production of agriculture and forestry commodities; (2) carbon emissions calculation, including emissions from deforestation over peatlands (through peatland drainage); and (3) quality assessment, examining the quality of the input data and its contribution to the model’s estimates (Extended Data Fig. 1).

The model generates annual deforestation and carbon emission estimates, along with an IQI for each country–commodity pairing at the national level (and subnational level for Brazil), adhering to the administrative boundaries defined by the Database of Global Administrative Areas (GADM) v.4.1^73^. Detailed information on the datasets used in this model is presented in Supplementary Table 2.

### Deforestation attribution

Spatial attribution utilizes a wealth of remote-sensing data to allocate tree cover loss to either specific commodities (for example, soybeans or oil palms), specific land uses (for example, croplands, pastures, forest plantations or mixed land-use mosaics) or broad deforestation drivers (for example, commodity-driven deforestation or forestry activities) (Extended Data Fig. 1). When the proximate cause of deforestation is not attributable to a single commodity via spatial attribution, we use statistical attribution based on agricultural and forestry statistics to attribute deforestation to specific commodities (Supplementary Fig. 1).

#### Spatial attribution

We begin by defining forest and deforestation. Here, forests are defined as trees established through natural regeneration^3^, and the conversion of these natural forests to other land uses is referred to as deforestation^3^. This definition of forest excludes forest plantations that are intensively managed for wood, fibre and energy^3^. To delineate these categories, we use the GFC dataset^28^ (Extended Data Fig. 1) which defines tree cover based on the presence of woody vegetation exceeding 5 m in height, with tree cover loss representing the replacement of woody vegetation within each 30-m pixel. Recognizing that not all woody vegetation constitutes natural forest, we adopt a tree cover density threshold of ≥25% per pixel^74^ (although this threshold can be adjusted to suit varying definitions of forest and deforestation, as shown in Table 1) and apply a global forest plantation mask (Supplementary Fig. 7; see discussion in Supplementary Information, Forest plantation mask) to distinguish loss of natural forests from managed forests (for example, rotational clearing of forest plantations). Pixels not meeting this natural forest criterion are excluded from further assessments.

It is important to recognize that the GFC dataset provides tree cover density values only for the year 2000. Although this aligns with our objective of evaluating deforestation from 2001 onwards, the dataset offers annual estimates of tree cover loss (for the first loss event), but not gain (necessary to account for forest regrowth) and subsequent losses. Consequently, losses of secondary forests—those regenerating naturally after the removal of native natural forests—established post-2000 are therefore not captured. Although global estimates of secondary forest loss and carbon emissions are largely unknown, studies on the Brazilian Amazon indicate that losses of primary and secondary forests have been quite comparable over the past two decades (both between 1 and 1.5 Mha yr^−1^)^75^, with secondary forest loss can contributing up to 11 ± 3.7 MgCO_2_ ha^−1^ yr^−1^ in emissions^76^. However, the lack of annual forest-regrowth data prevents us from incorporating it into the DeDuCE modelling framework. Secondary regrowth will only be included if it was already classified as forest in the year 2000—as can occur in older deforestation frontiers with extensive agricultural-land abandonment, such as in the Brazilian Amazon (Supplementary Note 5).

To assess the contribution of agricultural and forestry activities to annual deforestation, we overlay different land-use products that demarcate cropland^77^, forest plantation^78^ and pasture extents^79^, and crop commodities such as soybeans^16^ and cocoa^80^ on an annual tree cover loss layer^28^ spanning from 2001 to 2022 (Extended Data Fig. 1 and Supplementary Table 2; see discussion in Supplementary Information, Processing temporally explicit and temporally aggregated datasets). Through this, we gain insights into (1) whether a given pixel of forest loss constitutes deforestation and (2) what was the proximate cause of that deforestation (Extended Data Fig. 1).

To ensure a coherent integration of this data, we use a hierarchical attribution based on a scoring system that evaluates each dataset’s relevance based on spatial coverage, temporal frequency, and the specificity of deforestation driver and causation (that is, explicitness) (Supplementary Table 6). Further particulars of this scoring system are delineated in ‘Quality assessment’ below, but for each forest-loss pixel, we prioritize the most detailed information on the direct cause of forest loss. This means that we prioritize spatial data on specific agricultural commodities, then broader land-use categories, and finally general or dominant forest-loss drivers. Whenever datasets overlap in content (similar land use or commodity), those with higher spatiotemporal resolution take precedence. Furthermore, our model refrains from attributing forest loss to spatial data beyond the most recent year of available information, ensuring that our analysis reflects the latest land-use status. This approach ensures that once a pixel’s forest-loss driver is accounted for, it is no longer considered in the further attribution process.

In the final step of the spatial attribution, we address forest loss resulting from fires, a natural process crucial for ecological equilibrium, particularly in boreal regions. We systematically remove fire-related forest loss from our deforestation attribution, using spatiotemporal data^44^ that identify such events. Additionally, for regions not captured by the commodity and land-use datasets listed in Supplementary Table 2, we employ a global dataset by Curtis et al.^24^ that identifies the dominant drivers of forest loss (supplemented with the global forest plantation mask to segregate natural forest loss from the rotational clearing over managed plantations post-2000; Supplementary Fig. 7). All preprocessing methodologies applied to these spatial datasets are detailed in Supplementary Table 7.

The result of the spatial attribution is a dataset that summarizes, at the (sub)national level, the amount of deforestation attributed to specific commodities (soy, maize, sugarcane), land uses (croplands, pastures or forest plantations) or as the mosaics of multiple land uses, and deforestation drivers (Extended Data Fig. 1). The entire process of spatial deforestation attribution, involving the analysis of terabytes of spatial data, is conducted utilizing GEE.

#### Statistical attribution

Despite spatial attribution, considerable deforestation remains unclassified to specific commodities. This occurs for three main reasons: (1) when we have specific land-use information indicating the cause of deforestation is either a cropland, pasture or forest plantation; (2) the presence of land-use mosaics, specifically the MapBiomas^79^ dataset, which identifies pixels as a cropland and pasture mosaic when the algorithm cannot distinctly separate the two, or the Curtis et al.^24^ dataset, which determines the primary driver of forest loss aggregated over a 22-year period; or (3) instances where forest loss is not linked to any specific commodity or land-use by the existing spatial datasets (Supplementary Table 2).

To attribute forest loss to a specific commodity in these cases, we follow a two-step statistical land-balance approach (Supplementary Fig. 1). We first attribute deforestation in the latter two cases to either cropland, pasture or forest plantations. This method utilizes annual land-use data from FAOSTAT^31^ and FRA^3^ to inform on the extent of land-use expansion in these indeterminate areas of deforestation (referred to as ‘statistical land-use attribution’ in Extended Data Fig. 1; discussed in Supplementary Information, Statistical land-use attribution; Supplementary Note 6). Second, we further attribute cropland-driven deforestation to various crop commodities according to their respective increases in harvested area (again using FAOSTAT^31^; referred to as ‘statistical commodity attribution’ in Extended Data Fig. 1 and Supplementary Fig. 1). Similarly, deforestation from pasture expansion is allocated between cattle meat and leather. Deforestation attributed to forest plantations is allocated broadly to forestry products, due to the absence of detailed forestry-commodity statistics. See Supplementary Information, Statistical commodity attribution for a detailed discussion.

### Carbon emissions calculation

To calculate carbon emissions, excluding those from peatland drainage, we assess changes in carbon stocks due to forest loss. Our analysis concentrates on five key stocks: aboveground biomass (AGB), belowground biomass (BGB), deadwood, litter and soil organic carbon (SOC) (Extended Data Fig. 1). Notably, BGB and SOC losses are typically delayed responses to aboveground disturbances^22^. However, for the purpose of our analysis, these losses are treated as if they are an inevitable consequence of the deforestation, often referred to as ‘one-off’ or ‘committed’ losses. Essentially, it implies that once a region is deforested, the belowground carbon and associated SOC is also considered lost, even though this process might happen slowly over time.

AGB per pixel (in Mg px^−1^) is derived from the aboveground live biomass density data for year 2000 at 30-m resolution^33^. Based on this spatial AGB map and a 1-km-resolution map of root-to-shoot biomass ratio^81^, we estimate BGB. Deadwood and litter biomass densities are also spatially calculated as proportions of AGB, informed by biome-specific look-up tables that factor in elevation and precipitation (look-up table in ref. ^33^) (Supplementary Table 2). These biomass densities are converted to carbon densities (that is, MgC px^−1^) using a standard biomass-to-carbon conversion ratio of 0.47 for forest ecosystems, as recommended by the IPCC^82^.

For spatially attributed commodities, carbon emissions are calculated by overlaying forest-loss pixels onto the corresponding total carbon stock maps. For statistically attributed commodities, emissions are apportioned based on their proportion to the total forest loss associated with that commodity’s land use (that is, if maize’s statistically attributed forest loss accounts for 50% of all forest loss from croplands, maize would also bear 50% of the total carbon emissions statistically attributed to cropland-driven deforestation; Supplementary Information).

SOC stock data are obtained from the SoilGrids2.0 dataset^34^, which provides SOC stocks at different depths at 250-m resolution (in MgC ha^−1^). To avoid double-counting carbon emissions, we exclude SOC pixels overlapping with the global peatland map from our analysis. Emissions from peatland drainage linked to deforestation are analysed separately, as described below. For our purposes, we consider SOC within the top 100 cm of soil, the layer most affected by land-use changes, and downscale this data to a 30-m resolution (estimates expressed in MgC px^−1^). In light of limited data on SOC losses over deforested regions, we adopt an alternative approach informed by meta-analyses, which indicates that converting natural forests to either a cropland, pasture or forest plantation will typically result in decreased SOC stocks. Consequently, we represent SOC loss as a fraction of the existing SOC stocks for different replacing land uses and biomes (Supplementary Table 8). These SOC emissions are then added to the carbon emissions calculated from AGB, BGB, deadwood and litter (equation (1)).

We then deduct the committed carbon sequestration potential of the replacing commodity (for example, carbon stored as vegetation biomass if the replacing land use is maize or forest plantation) (equation (1)). This deduction is informed by a meta-analysis of mature plant carbon stocks across commodities (in MgC ha^−1^), and is categorized into 40 commodities across 11 commodity groups (Supplementary Table 9). If data for a specific commodity are absent, we refer to the plant carbon stocks of its respective commodity group (Supplementary Table 9). These values represent the maximum carbon storage in the replacing crop systems, while the time-averaged sequestration before harvest (often lower than the maximum carbon stock) will vary between crops and management systems (for example, the share of carbon stored in roots and residues and how the latter are managed will influence carbon turnover times in annual crop systems; the length of rotation periods and pruning frequency will impact average carbon stocks in perennial crop systems), which means that our estimates of net emissions are conservative.

The resulting net carbon emissions are then expressed in MtCO_2_:

1 $$\begin{array}{l}\begin{array}{l}\mathrm{net}\;\mathrm{carbon}\\ {\mathrm{emissions}}\end{array}=\mathrm{AGB}+\mathrm{BGB}+\mathrm{deadwood}+\mathrm{litter}+\mathrm{SOC}\;\mathrm{loss}\\\qquad\qquad\quad-\begin{array}{l}\mathrm{plant}\;\mathrm{carbon}\;\mathrm{stocks}\; \mathrm{of}\\ \mathrm{replacing}\;\mathrm{commodity}\end{array}\end{array}$$

#### Peatland drainage emissions

To align with the deforestation attribution analysis, our model concentrates on carbon emissions from deforestation occurring on peatlands post-2000, deliberately excluding continuous emissions from peatlands deforested earlier. By superimposing a high-resolution global peatland map (a composite map prepared from multiple sources at 30-m resolution^35^) onto identified forest loss, we isolate peatland deforestation linked to specific commodities and land-uses post-2000 (Extended Data Fig. 1). In the presence of spatial commodity data, overlapping peatland deforestation is directly attributed to the corresponding commodity. In their absence, however, we evenly allocate deforested peatland areas among all identified commodities expansions within a country (similar to statistical attribution).

To estimate the emissions from peatland drainage, we use emission factors reported by published literature (often represented in MgCO_2_ ha^−1^ yr^−1^). These factors are informed by subsidence observations and standardized rates of peat oxidation, providing a scientifically grounded approach to these emission factor calculations^82,83^. Based on previous meta-analyses of peatland emission factors^82–85^ (Supplementary Table 10), we have stratified emission factors by land-use expansions (such as peatland drainage due to cropland, pasture or forest plantation expansions; or oil palm expansions specifically) and deforestation biome (that is, tropical, temperate and boreal), which allows us to apply these factors to specific drainage conditions for different biomes. We multiply these emission factors with peatland drainage area (result expressed in MgCO_2_ yr^−1^). Unlike committed emissions, these peatland drainage emissions continue to accumulate, year on year, from the initial deforestation event until the conclusion of our study period (discussed in Supplementary Information, Peatland drainage emissions). For instance, a hectare of peatland cleared and drained for oil palm in 2010 incurs annual emissions of 54.41 MgCO_2_ every year until 2022.

In addition to providing annual (that is, unamortized) deforestation and carbon emission estimates for country–commodity pairings, we also present amortized estimates (excluding peatland drainage emissions). For amortization, we distribute these estimates evenly over a 5-year period. This amortization aligns the temporal scale of deforestation’s impact with the timeframe of agricultural production, offering a more nuanced understanding of the long-term environmental footprint of crop cultivation and forestry activities^86,87^ (Supplementary Note 7).

### Quality assessment

Our methodology integrates multiple spatial and statistical datasets, making it necessary to assess the quality or reliability of our deforestation estimates aggregated for each country–commodity pairing (Fig. 5 and Extended Data Fig. 1). To this end, we developed an IQI designed to reflect the relative confidence in each deforestation estimate. Combining multiple criteria, it measures how much the input data deviates in relation to, what we consider, the ideal characteristics of input data required to precisely quantify direct drivers of deforestation (dLUC), namely the highest spatial and temporal resolution, explicitness, and full accuracy (equation (2) and Supplementary Table 11). This assessment should not be confused with just the accuracy of underlying datasets or the model’s deforestation estimates because the latter is particularly challenging to assess deterministically for an integrated dataset of this scale and comprehensiveness. To quantify the quality of our deforestation estimates, we take into account three factors (equation (2)):

1. Forest loss or deforestation (FL_*i,t*_) attributed to a specific commodity (*i*) in a specific region and year (*t*).
2. Overall accuracy (OA_*j*_) of the input dataset (*j*), which contributed to the aggregation of final deforestation estimates. This value is provided by the respective studies and datasets (Supplementary Table 2) and is assumed to encompass all aspects of input data’s accuracy. Thus, FL_*i,j*_ represents the contributions from each input data source (*j*) to the deforestation estimates attributed to a specific commodity (*i*).
3. Score_*j*_, a metric developed by us to normalize OA_*j*_ and make it comparable between all the input datasets of different types (that is, remote sensing-based and statistical) (Supplementary Information, Scoring metric justification, and Supplementary Table 6). This normalization hinges on three pivotal (and equally weighted) criteria assessing each input dataset’s spatial and temporal granularity, as well as explicitness or specificity of deforestation driver (Supplementary Table 11).

Spatially, a maximum score (of 1) is assigned to datasets with a resolution ≤10-m, tailored to individual countries. Temporally, annual datasets from 2001 to 2022 for herbaceous crops, and comprehensive data from 2000 or earlier for tree crops and forest plantations receive the top score. For tree crops and forest plantations, data from the year 2000 or earlier allow us to distinguish post-2000 deforestation from rotational clearing, thus removing the need for plantation masking. For explicitness, datasets mapping a singular agricultural or forestry commodity, validated by field data, are scored highest. Any fluctuation from these conditions results in the score of the dataset being penalized. The detailed scoring criteria are given in Supplementary Table 11.

This approach above works well when only spatial commodity datasets contribute to deforestation estimates (dLUC) (equation (2); and see discussion in Supplementary Information, Calculation of Integrated Quality Index (IQI)). However, the datasets we use also include broad spatial land-use information, which, when combined with agricultural land-use and commodity production statistics, provide estimates of commodity-driven deforestation (sLUC). In such cases, it is crucial to reflect the reliability of these agricultural statistics in the quality of our deforestation estimates. Because FAOSTAT does not provide overall accuracy, but report Flags—a qualitative assessment of the reported value (see the description of FAOSTAT flags in Supplementary Table 12)—we incorporate them into our quality assessment framework. We achieve this by multiplying the overall accuracy of the spatial land-use dataset (OA_*j*_; Supplementary Table 6) with the agricultural statistics quality flags (equation (2); and see discussion in Supplementary Information, Calculation of Integrated Quality Index (IQI)). Within these quality flags, data reported by official sources to FAOSTAT receive the highest score, while those that are estimated, imputed or extracted from unofficial sources are assigned progressively lower scores (Supplementary Table 12).

2 $$\begin{array}{l}{\rm{IQI}}_{i,t}=\displaystyle\frac{\mathop{\sum}\nolimits _{j=1}^{n}{({\mathrm{FL}}_{i,j}\times {\mathrm{OA}}_{j}\times {\mathrm{Score}}_{j})}_{t}}{{\mathrm{FL}}_{i,t}},\\{\mathrm{OA}}_{j}\,=\left\{\begin{array}{ll}{\mathrm{OA}}_{j} & \,{\mathrm{if}}\;{\mathrm{only}}\;{\mathrm{spatial}}\;{\mathrm{commodity}}\\&{\mathrm{datasets}}\;{\mathrm{contribute}}\;{\mathrm{to}}\,\\&{\mathrm{deforestation}}\;{\mathrm{attribution}}\\ {\rm{OA}}_{j}\times \left(\displaystyle\frac{{\mathrm{Flag}}_{\mathrm{land}\mathrm{use}}+{\mathrm{Flag}}_{\mathrm{production}}}{2}\right) & \qquad \mathrm{otherwise}\end{array}\right.\end{array}$$

In the DeDuCE model’s two-step land-balance approach, we use two agricultural statistics. Here, Flag_land use_ and Flag_production_ represent the quality of land-use and commodity production data, respectively. It is important to note that the IBGE dataset for Brazil does not provide flags for commodity production (Flag_production_). Thus, we assign a value of 1, reflecting the official figure flag as IBGE directly reports the data. Examples of IQI calculations under various scenarios are provided in Supplementary Information.

Note that while a higher IQI indicates greater confidence that a data point approximates direct deforestation (dLUC) for a given commodity and country, it should not be interpreted as a direct measure of accuracy. In some cases, high-IQI estimates may still diverge from reference data, whereas low-IQI estimates may align by chance or compensating biases (Supplementary Note 3 and Supplementary Table 3). Such discrepancies can arise from methodological differences or from limitations in spatial datasets that imperfectly capture land-use transitions, and in agricultural statistics that reflect statistical attribution (sLUC) rather than direct, spatially verified deforestation (dLUC).

### Reporting summary

Further information on research design is available in the Nature Portfolio Reporting Summary linked to this article.

## Supplementary information

Supplementary Information Supplementary Figs. 1–7, Tables 1–12 and Notes 1–7.

Reporting Summary

## Source data

Source Data Fig. 1 Source data.

Source Data Fig. 2 Source data.

Source Data Fig. 3 Source data.

Source Data Fig. 4 Source data.

Source Data Fig. 5 The source data for Fig. 5 are divided into two parts. The source data for Fig. 5a are archived on Zenodo while that for Fig. 5b are accessible here.

## Data Availability

The deforestation and carbon emission estimates generated by the DeDuCE model, including those from sensitivity analyses, are available from Zenodo via 10.5281/zenodo.13624636 (ref. ^88^). The trade analysis presented in Extended Data Fig. 2 is available from Zenodo via 10.5281/zenodo.10633818 (ref. ^89^). All the datasets used in this study are documented in Supplementary Table 2. The insights from the DeDuCE model can be viewed at https://www.deforestationfootprint.earth. Source data are provided with this paper.

## Code availability

The Google Earth Engine and Python code for running the DeDuCE model, and the code needed to replicate the analysis presented in this study, are available at GitHub: https://github.com/chandrakant6492/DeDuCE.
