# Supplementary material for: Global patterns of commodity-driven deforestation and associated carbon emissions
Source: Nat Food. 2026 Feb 23;7(2):138–51. doi: 10.1038/s43016-026-01305-4 (PMC12935532; doi:10.1038/s43016-026-01305-4)
Supplement: Supplementary file 1 — Supplementary Figs. 1–7, Tables 1–12 and Notes 1–7. [file 43016_2026_1305_MOESM1_ESM.pdf]

nature food

Supplementary information https://doi.org/10.1038/s43016-026-01305-4

Global patterns of commodity-driven deforestation and associated carbon emissions

In the format provided by the authors and unedited

Table of contents

A. Supplementary Notes 2

1. Why combine spatial data with agricultural statistics for global deforestation attribution? 2
2. Replicability and community engagement 3
3. Lack of clear and explicit guidelines on data and methods may hinder deforestation and carbon emission accounting 4
4. Inherent risk of combining different spatial and statistical datasets 5
5. Comparison of DeDuCE deforestation estimates with high-quality remote sensing-based studies 6
6. Multi-cropping and commodity-specific harvested area bias 7
7. Intention behind amortised and unamortised estimates 8

B. Supplementary Methods 9

1. Forest plantation mask 9
2. Processing temporally explicit and temporally aggregated spatial datasets 10
3. Statistical land-use attribution 10
   - 3.1 Estimating gross land-use expansion 10
   - 3.2 Handling land-use mosaics 11
   - 3.3 Capping deforestation due to forestry activities 12
   - 3.4 Gap filling 12
4. Statistical commodity attribution 13
   - 4.1 Deforestation attributed to crop commodities 13
   - 4.2 Deforestation attributed to pasture commodities 14
   - 4.3 Deforestation attributed to forestry commodities 14
5. Peatland drainage emissions 14
6. Quality assessment 15
   - 6.1 Scoring metric justification 15
   - 6.2 Calculation of Integrated Quality Index (IQI) 15

C. Supplementary Figures 18

Supplementary Fig. 1 | Visual representation of the statistical deforestation attribution (i.e., two-step land balance model) 18

Supplementary Fig. 2 | Geographical overview of commodity-driven deforestation (2001-2022) 19

Supplementary Fig. 3 | Comparison of deforestation estimates of major deforestation-risk commodities and countries with other studies. 20

Supplementary Fig. 4 | Integrated Quality index (IQI) of major deforestation-risk commodities as shown in Fig. 4. 21

Supplementary Fig. 5 | Hotspots of major deforestation-risk commodities for Brazil (aggregated for 2018-2022) 22

Supplementary Fig. 6 | Contribution of peatland drainage emissions to total deforestation carbon emissions 23

Supplementary Fig. 7 | Framework for distinguishing natural forest loss and loss over managed forests.. 24

D. Supplementary Tables 25

Supplementary Table 1 | Country and commodity groups with their respective deforestation-carbon emission estimates and integrated quality index (2001-2022). 25

Supplementary Table 2 | Datasets used in this study and their description 26

Supplementary Table 3 | Comparison of DeDuCE deforestation estimates with remote sensing datasets. 28

Supplementary Table 4 | Summary of the datasets and models used for deforestation and carbon emission comparisons in Fig. 2. 30

Supplementary Table 5 | Absolute values of deforestation and carbon emission estimates used for sensitivity analysis. 32

Supplementary Table 6 | Scoring individual datasets for attribution and quality assessment 36

Supplementary Table 7 | Pre-processing and attribution assumptions for the spatial datasets.......... 37

Supplementary Table 8 | Loss of soil organic carbon (SOC) across different land use and biomes 39

Supplementary Table 9 | Plant carbon stocks of replacing commodities and commodity groups across different biomes 39

Supplementary Table 10 | Emission factor used to estimate carbon emissions from deforestation on peatlands 40

Supplementary Table 11 | Criteria's for scoring different aspects of spatial datasets 41

Supplementary Table 12 | The FAO flags, their description and associated penalisation.......... 42

Supplementary references 43

A. Supplementary Notes

1. Why combine spatial data with agricultural statistics for global deforestation attribution?

Spatial datasets, such as land-use and land-cover products, that are derived from remote sensing and geospatial analysis, offer considerable advantages for deforestation assessment by providing high-resolution, wall-to-wall coverage across broad landscapes[1,2]. They enable direct observation and quantification of land-use changes over time, often at fine spatial and temporal scales, allowing the identification of deforestation hotspots[3,4], patterns of landscape fragmentation[5], and the dynamics of land conversion[6]. Moreover, spatial datasets—whether originating directly from or derived from satellite imagery—offer objective, repeatable, and relatively unbiased measurements, independent of national reporting biases or inconsistencies. This makes them crucial not only for monitoring deforestation at large scales, but also in remote or politically sensitive areas where field data and on-the-ground monitoring and verification efforts are limited.

Despite these advantages, producing large-scale land-cover or land-use maps remains challenging due to inconsistencies in definitions (e.g., what constitutes a forest or pasture)[7], sensor limitations (affecting what we can observe, how clearly, and how frequently), and classification ambiguities (i.e., the reliability of interpreting satellite-derived information). For example, similar land-use types, such as natural forests and plantations or pasture and grasslands, can be difficult to distinguish remotely, often leading to misclassification without appropriate field validation[8,9]. Temporal inconsistencies driven by dynamic land-use changes and varying data quality across regions further complicate global mapping efforts[10]. Additionally, a lack of comprehensive ground-truth validation in many areas undermines both the product development and the accuracy of such products[11]. Collectively, these challenges limit the development

of reliable spatial datasets, which differ in their spatio-temporal scope (i.e., what land cover or land use can be mapped, and when and where), explicitness (the depth of detail captured about land systems), and accuracy (reliability with respect to ground truth).

In contrast, statistical datasets, such as agricultural production statistics[12,13], leverage national- or sub-national level inventories, production surveys, and trade information to capture broader economic drivers and sectoral dynamics that may not be immediately visible from spatial data alone. For example, FAOSTAT, one of the largest archives of agricultural statistics, gathers data through annual questionnaires distributed to FAO member countries, national publications, official websites, and, in some cases, expert observations and elicitation[14]. Although these statistical datasets lack fine spatial resolution—typically aggregating information to national or sub-national boundaries—they provide valuable insights into commodity production for a wide range of crops and livestock, offering contextual understanding of global land-use dynamics that purely spatial datasets cannot yet achieve.

Given the current state of the world, where rapid deforestation poses major challenges to achieving global climate and biodiversity targets[15]—directly impacting future food security and a habitable climate[16,17]—there is a pressing need for a reliable global dataset that can identify drivers of deforestation at the finest possible scales (often necessary for crafting actionable strategies and guiding policy making) across all relevant commodities. It is essential not just to monitor deforestation linked to commodities for which spatial data are more readily available or of higher accuracy, but to systematically link deforestation to the full spectrum of commodities and economic sectors to inform relevant policy actions for forest conservation and climate action; particularly critical for tracing indirect drivers of deforestation, such as displacement effects and complex supply chain dynamics[18,19].

While we recognise that a globally consistent, pixel-level dataset linking deforestation to individual commodities remains an ideal goal, given current limitations—including spatial and statistical data constraints, definitional inconsistencies, and modelling complexities—it is unlikely that such a dataset will be fully realised in the immediate future. Nonetheless, improvements in the spatial representation of individual commodities[20–22], finer-scale statistical reporting (which is now transitioning from national to sub-national level[23]) and spatial-statistical data fusions[24–28] are expected to advance considerably in the near term. With the DeDuCE model, we combine the best available spatial and statistical datasets (be it at national, sub-national or pixel scale) as they become available, aiming to attribute deforestation as accurately as possible to specific commodities and geographies, while recognising that the precision of attribution necessarily varies by country and commodity depending on data availability and quality.

Such a hybrid approach acknowledges that fully consistent representation across the globe is currently unachievable, as different datasets have different spatio-temporal niches and perform differently across regions and time, even when underlying conditions (e.g., satellite imagery sources, field sampling methods, or training algorithms) are similar. However, this variability also offers an opportunity: it allows us to evaluate how methodological differences in the underlying data, assumptions, and modelling biases influence the final deforestation estimates, thereby highlighting areas where future improvements in land-use change monitoring are most needed (Fig. 5).

2. Replicability and community engagement

Replicability is a key objective of our modelling approach. We have built the model using two widely adopted, open-access programming structures: **Google Earth Engine (GEE)** and **Python**. GEE enables users to process large volumes of satellite imagery and geospatial data directly in the cloud, eliminating the need for high-end hardware or local data downloads. All the spatial processing—such as applying tree cover thresholds, masking forest plantations, aligning land-use layers, integrating and reclassifying land-use information, and aggregating spatial inputs—is performed using documented, script-based functions.

In contrast to proprietary GIS platforms like ArcGIS, which often require licensed software and manual workflows, our approach relies on code-based operations that ensure that any user globally, with access to Google Earth Engine and the model code, gets the same result.

Furthermore, having a code-based modelling framework enables version control, which is essential for openly and transparently sharing modifications and future improvements with both developers and data users. For example, users can review the full change history of the DeDuCE model's codebase to see exactly what updates have been made and when, since its initial release.

We also recognise that not all users of the model or dataset will be experts in GIS, but might have critical feedback for the **modelling framework**. To address this, the model follows a modular design that allows users to intuitively understand its structure and modify key parameters—such as tree cover thresholds, lag periods, or disaggregation of different resolution datasets—without requiring deep technical expertise. Whereas users with an expertise in Google Earth Engine and Python can not only have more flexibility to modify core components, but are also welcome to help us **improve the performance of the model**. This includes customising the commodity classification logic, integrating emerging land cover datasets, and adjusting aggregation rules to suit different contexts more efficiently.

3. Lack of clear and explicit guidelines on data and methods may hinder deforestation and carbon emission accounting

Despite increasing attention to deforestation associated with global food consumption and its impacts on climate, there remains a lack of clear and explicit guidelines for deforestation–carbon emission accounting, particularly regarding the datasets and methodological choices needed to operationalise assessments. Major frameworks such as the forthcoming Land Sector and Removals Guidance from the Greenhouse Gas (GHG) Protocol[29] and the Accountability Framework Initiative (Afi)[30] provide general principles on how companies should account for land-use change emissions, but they stop short of prescribing specific datasets or offering explicit instructions for capturing the complex dynamics of food systems (e.g., the allowable time lag between deforestation and commodity-driven land use for it to be classified as direct land-use change, or the attribution of deforestation in a multi-cropping scenario). Similarly, the Science-Based Targets initiative (SBTi) Forest, Land, and Agriculture (FLAG) guidance[31], which outlines the requirement for companies to commit to zero deforestation, does not specify exact data sources or methods for monitoring deforestation, either through field sampling or remote sensing.

For example, while these guides broadly define 'forest' (i.e., land spanning >0.5 ha with trees >5 m in height and a canopy cover ≥10%, including trees capable of reaching these thresholds at maturity) and 'deforestation' (i.e., the loss of natural forest as a result of conversion to agriculture, tree plantations, or other non-forest land uses), **they do not assess whether these definitions can be operationalised using standardised global datasets**, such as the Global Forest Change (GFC) dataset or JRC's Tropical Moist Forest (TMF) dataset. Nor do they provide clear protocols for distinguishing natural forests from plantations when one chooses to use these datasets. This vagueness introduces considerable latitude for interpretation, e.g., by private companies and governments, enabling discrepancies in reported deforestation footprints depending on methodological choices—for instance, whether a 10%, 25%, or 30% canopy cover threshold is applied, whether secondary forests are included, or whether plantation conversions are classified as forest loss[32–34]. Consequently, entities reporting deforestation linked to commodity production may either underestimate or overestimate their impacts purely based on methodological differences, undermining comparability across organisations, sectors, and countries.

This lack of such standardisation has also become a point of debate in the European Union Deforestation-Free Regulation (EUDR)[35]. The EUDR requires companies to demonstrate that commodities placed on the EU market are not linked to recent deforestation; however, without consistent, globally accepted

definitions that can be operationalised through the available datasets, establishing compliance becomes challenging. Different actors may benchmark risk differently based on their choice of dataset (e.g., forest and deforestation), reporting methods (Table 1), or historical baselines[33]. This can allow for instances where a company using a more lenient forest definition or a less sensitive forest-loss dataset might classify an area as “non-deforested,” while another using a stricter dataset could reach the opposite conclusion. This inconsistency not only complicates supply chain verification but also financial risk assessment, as investors and insurers increasingly seek to understand their exposure to deforestation-linked assets. Without harmonised methodologies, risk benchmarking across countries and commodities will remain uneven, creating legal uncertainty and competitive distortions. Ultimately, the absence of clear and explicit guidelines on how forest and deforestation is defined (including other aspects of deforestation-carbon emission accounting), detected through different datasets/methods, and attributed to commodity production, undermines the effectiveness of both voluntary initiatives like SBTi[31] and mandatory regulations like the EUDR.

4. Inherent risk of combining different spatial and statistical datasets

We acknowledge that combining independent spatial and statistical datasets carries the risk of compounding inaccuracies (e.g., *in spatial datasets*: differences in resolution, classification schemes, and temporal coverage; *in statistical dataset*: data collection methods, definitions, and reporting standards), potentially leading to a misrepresentation of commodity-linked deforestation relative to actual ground conditions. A common solution for quantifying—and, in some cases, correcting—such inaccuracies is independent validation using high-quality reference data, as recommended by Olofsson et al. (2014)[36]. **However, such high-quality reference data are often sourced from detailed satellite imagery or field observations. The latter, in particular, remains sparse and unevenly distributed across the globe.** Olofsson et al.[36] emphasise that reference data used for such independent validations must be of higher quality than the data used for map classification. If the same source imagery is used for both the reference and the classification (e.g., if we use the same satellite image) **the reference must be generated using a demonstrably more accurate and rigorous method than the classification data itself.**

For our model, which tries to incorporate different spatial and statistical datasets to attribute deforestation globally, this standard is difficult to achieve, especially when working with datasets that themselves are based on extensive field sampling and sophisticated training algorithms (Supplementary Table 2). It is also worth noting that recent remote sensing-based assessments of commodity-specific deforestation remain scarce. Most focus on major forest-risk commodities in key producer countries—such as oil palm in Indonesia and soybeans in Brazil—and even among these, estimates often vary considerably. For other commodities (e.g., staple crops) and regions, particularly in Africa, such data is largely absent (Pendrill et al.[37] and Supplementary Table 4).

Therefore, developing—or even claiming to use—reference data of higher quality than that used to train existing algorithms poses practical challenges, especially across different geographies and commodities without substantial expertise in data collection and model training. Therefore, while independent validation remains an ideal goal, the above-mentioned constraints currently limit its application in this study. As a workaround for this limitation, we took a multi-pronged approach:

(a) we developed an ‘**Integrated Quality Index (IQI)**’, which by accounting for the reported accuracies of input datasets, provides a probabilistic estimate of the relative reliability of the resulting deforestation estimates (Fig. 5),
(b) conducted an extensive **sensitivity analysis** to evaluate how variations in key input assumptions and dataset choices influence model outputs (Table 1), and
(c) performed an extensive **comparative analysis** of several widely used and recently published remote sensing studies attributing deforestation to specific commodities (Supplementary Note 5), in order to contextualise and assess the robustness of our results.

Nevertheless, this limitation also underscores the importance of transparently documenting methodological choices and modelling assumptions, which we have tried to be explicit about in our study (see Methods and Supplementary Information), to highlight potential discrepancies. **While we inform users that they should interpret findings with reasonable caution, even in the absence of independent validation, spatial overlay approaches do provide valuable, spatially explicit insights into global-scale deforestation drivers.** These insights are particularly useful when applying these estimates to inform conservation targets, policy frameworks, or deforestation-risk benchmarking. Nevertheless, we firmly believe that future advances in remote sensing technology, increased availability of high-quality reference data, and broader collaboration among researchers and institutions may progressively address these constraints (Supplementary Note 2), ultimately enabling more robust independent validations for such deforestation attributions at global scales.

5. Comparison of DeDuCE deforestation estimates with high-quality remote sensing-based studies

Supplementary Table 3 compares the DeDuCE model's deforestation estimates against (i) the best available evidence on commodity-driven deforestation from studies based on remote sensing data, (ii) estimates from the sensitivity analysis of the DeDuCE model that does not use any spatial data except the tree cover loss layer (thus avoiding potential errors arising from combining spatial data layers; see Supplementary Note 4), and (iii) the Pendrill et al.[38] data, which represents the only other pan-tropical dataset for commodity-driven deforestation across multiple commodities and also does not use spatial data beyond forest loss and existing plantations. From this comparison, we draw the following conclusions:

First, among the three compared models—DeDuCE, DeDuCE non-spatial, and Pendrill et al.[38]— the base DeDuCE model (utilising spatial data) overall produces estimates closest to those of remote sensing-based studies (Supplementary Table 3). Most of the instances where the non-spatial DeDuCE model performs better than the full DeDuCE model involve comparisons with the estimates from Masolele et al.[39] analysis for Africa. A likely reason is that this study simply uses Hansen tree cover loss as a deforestation layer (i.e., it includes tree cover loss in existing plantations and—importantly in Africa—existing shifting cultivation systems) just as the non-spatial DeDuCE model does, whereas the full DeDuCE model attempts to identify deforestation by excluding rotational clearing.

Second, the DeDuCE model performs best—both in absolute terms and relative to the other two datasets—when it utilises commodity-specific spatial maps (Supplementary Table 3). This includes Indonesian oil palm, South American soy, and cocoa in West Africa, which are all major deforestation commodities and regions. Typically, the DeDuCE model provides estimates within 20–30% of those from high-resolution studies when it uses multiple spatial data layers. The main exception is Brazilian beef. Since the comparison studies use the same pasture map as DeDuCE (i.e., MapBiomas), the reason for the large discrepancy here is likely due to the fact that the deforestation layer (i.e., PRODES data) used by the comparison studies excludes clearing of secondary forest. However, a recent study[40] estimates that clearing of secondary forests in the Brazilian Amazon averaged approximately 1.4 Mha yr^{-1} between 2001 and 2014—driven primarily by pasture expansion—highlighting their substantial contribution to overall deforestation. Similarly, the Trase estimate of deforestation for soy in Bolivia is substantially lower than that of DeDuCE, which is likely due to the fact that the Trase estimates do not cover deforestation in the Chiquitano dry forests (only in the Amazon biome, as defined by RAISG, and the Gran Chaco) and exclude isolated soy deforestation of less than 5 ha[41], both of which are captured by DeDuCE. The discrepancy caused by differences in the deforestation data was particularly evident in the case of cocoa in Côte d'Ivoire. When we switched from GFC to TMF data (analysed as part of the sensitivity analysis; see Table

1), the difference in estimates dropped to only -16%, compared to -54% from Trase[42] estimates (which also use TMF deforestation data; Supplementary Table 3).

Third, the performance of the DeDuCE model drops in cases where deforestation estimates are based on limited spatial data (i.e., no spatial data on post-forest-loss land use). This includes pasture and cashew nuts in Africa (cashew, as a tree crop, is not included in the global cropland map used in DeDuCE). Still, the DeDuCE model provides better results than the Pendrill et al. dataset it replaces —especially for pasture expansion in Africa.

Comparing the trends between DeDuCE, Pendrill et al.[38] and WRI[43] deforestation estimates reveal that most of these differences are relatively moderate at regional or pan-tropical scales (Supplementary Table 3)—where aggregation tends to smooth out methodological differences—but could become pronounced at finer scales (Supplementary Fig. 3). These discrepancies arise from several factors, including differences in the underlying datasets (e.g., using a different forest and deforestation layer than GFC[3]; or WRI using other spatial datasets such as MapSPAM[44] and EarthStat[45]). Differences in deforestation estimates when compared to independent remote sensing studies (Supplementary Table 3), which assume a different time lag between deforestation and commodity establishment (e.g., 5 years for soy, 10 years for oil palm and 15 years for cocoa in Trase) or handle indirect drivers differently (such as the displacement of food crops in Ghana's cocoa sector[46]), suggest that these methodological choices substantially influence final deforestation estimates. For policy and sustainability reporting—such as the assessment of deforestation risk in the supply chain (e.g., under EUDR compliance)—the choice of dataset and attribution methodology can greatly influence outcomes. Thus, this comparison further reinforces the need for more transparent, standardised, and commodity-specific accounting guidelines for deforestation and carbon emissions.

While discrepancies between different datasets highlight challenges in achieving fully harmonised deforestation and carbon emission accounting, they also offer valuable opportunities to better inform policy and conservation strategies. Each dataset brings unique methodological strengths that, when interpreted carefully, can uncover different dimensions of deforestation dynamics. For instance, datasets with shorter attribution periods are better suited for identifying recent land-use changes and evaluating near-term policy interventions. Conversely, datasets with longer attribution windows or broader spatial coverage can reveal legacy effects of land-use change and longer-term commodity-driven pressures on forests. Comparing multiple datasets thus enables policymakers and conservation practitioners to understand not only the immediate impacts of agricultural expansion, but also more complex patterns such as delayed commodity establishment, indirect land-use change, or secondary forest regrowth. **Such triangulation helps avoid blind spots that a single dataset might miss and promotes a more nuanced, evidence-based approach to forest conservation.** Moreover, it encourages greater transparency in reporting and stimulates methodological innovation, ultimately contributing to more robust national targets, corporate sustainability goals, and international agreements aimed at halting deforestation.

6. Multi-cropping and commodity-specific harvested area bias

FAOSTAT commodity-specific agricultural statistics[12] report harvested area, which can exceed the physical cropland area when multiple crops are grown sequentially on the same land. This distinction is crucial when linking cropland expansion to deforestation, as it can lead to double-counting of area in multi-cropped systems (both sequential and intercropping) and, consequently, to potential biases in crop-specific attribution (see 'Statistical land-use attribution' discussion in Supplementary Methods). A recent global estimate of multi-cropping systems indicates that approximately 12% of cropland is harvested more than once annually, including 34% of rice, 13% of wheat and 10% of maize areas[47]. Such cropping intensity patterns are particularly pronounced in South, East, and Southeast Asia, where wheat-rice-based rotations are dominant, and in parts of Latin America with double-crop maize and soybean

systems[47]. However, the apparent expansion estimated through FAOSTAT agricultural statistics may only indicate the increased conversion of new land to cropland, rather than an increase in cropping frequency.

Although this limitation does not influence the total deforestation area attributed to agriculture in the present study—since the DeDuCE framework restricts overall cropland expansion using spatial data and FAOSTAT land-use statistics (which report physical cropland area)—it may affect how deforestation is distributed among different crops, particularly those grown in multiple cycles per year. **The DeDuCE model addresses this issue only in Brazil, where subnational data from the Brazilian Institute of Geography and Statistics (IBGE)[13] provides multiple harvests for selected crops.** For these crops—specifically maize, groundnuts, beans, and potatoes—we use only the first harvested area to avoid overestimating their contribution to deforestation (see 'Statistical land-use attribution' discussion in Supplementary Methods). For other countries, such disaggregation is not yet possible due to the lack of subnational or globally harmonised data on cropping intensity.

The multi-cropping sensitivity analysis for the DeDuCE model reveals deviations of 7–35% when multiple cropping is not considered in the deforestation attribution framework (Table 1). These discrepancies imply that part of the statistically inferred increase in harvested area can be misattributed to frontier expansion rather than changes in cropping intensity on existing cropland. While substantial work remains to reconcile discrepancies arising from multi-cropping[48], a promising path forward involves integrating physical crop area (rather than commodity-specific harvested area) with emerging datasets such as FAO CROPGRIDS[24] (harvested area of 173 crop commodities at 10 km resolution) and seasonally resolved cropland activity from ESA WorldCereal[49] (pixel-level data on maize and cereals). This combined approach would enable systematic correction for harvested-area bias and support more robust, crop-specific deforestation attribution as better data becomes available.

7. Intention behind amortised and unamortised estimates

When a forested land is cleared, the majority of carbon is released during the initial clearing, while emissions from subsequent decay of biomass continues over the next few years. Thus, in environmental impact assessments, particularly regarding the impact of deforestation, it's crucial to consider not just the immediate impact of forest loss, but also the extended effects of this transformation[38,50]. Consequently, the deforestation emissions presented here are 'committed emissions', reflecting the long-term change in biomass carbon stocks due to the land-use change from forest to agricultural or forest plantation land-use, including adjustments in soil carbon contents and carbon sequestration in tree crops, for instance.

When attributing these emissions to commodities produced on cleared forest land—calculating a 'deforestation carbon footprint'—these committed emissions from the land-use change event must be distributed over the production period. This is achieved using an 'amortisation' period, which conceptually distributes the consequences of deforestation (i.e., committed emissions) across multiple years to account for the enduring productivity of the land. This is a common practice in land-use change-related impact assessments (e.g., IPCC[51], GHG Protocol[29]) and here this approach is adopted for calculating the estimates of deforestation emissions embodied in international trade, displayed in Extended Data Fig. 2.

Interestingly, several studies have criticised the use of an amortisation period for its arbitrary nature and weak scientific justification[52]. Since its introduction for GHG accounting (IPCC, 1996[53]), a 20-year amortisation period has been commonly used, albeit non-mandatory. The IPCC guidelines[53] explicitly state that "*the choice of a 20-year period represents a compromise*", and that amortised carbon emissions may not adequately capture the underlying biophysical processes related to carbon balance[52]. Following ref.[38], we adopt a shorter, 5-year amortisation period to better capture the immediate effects of

deforestation while also allowing for the analysis of the dynamic nature of current food systems, such as the influence of recent consumption patterns on deforestation (exemplified in Extended Data Fig. 2). However, our choice of a 5-year amortisation period does not impact the core DeDuCE model estimates, i.e., the annual emissions from deforestation attributed to commodities. Stakeholders have the flexibility to use this unamortized data to calculate emissions for any amortisation period that aligns with their reporting standards and requirements.

Furthermore, understanding these annualised/unamortised and amortised estimates helps balance immediate actions with long-term planning in climate change mitigation efforts. For example, commodities associated with peatland emissions require continuous (or annualised) monitoring and long-term regulatory measures. This approach enables policymakers to respond swiftly to sudden spikes in emissions, which is essential for implementing urgent regulatory actions. To identify and prioritise the most critical cases for intervention—particularly commodities causing substantial near-term deforestation, such as palm oil and cattle meat—unamortized emission estimates are more effective. Amortisation, by its nature, tends to smooth out the temporal dynamics of land-use change, potentially obscuring the urgency of recent impacts. For this reason, unamortised emissions highlight annual fluctuations, which are crucial for detecting trends and anomalies in specific commodities or regions. Understanding this annual variability is essential for grasping the dynamic nature of deforestation and its impact, thus facilitating more responsive and effective policy measures.

In contrast, amortised emissions (e.g., AGB, BGB, etc.) linked to deforestation might benefit from the development of intervention strategies, informing more targeted climate-change mitigation efforts and encouraging the adoption of sustainable practices[52]. Amortisation accounts for these annualised variabilities in deforestation emissions and assists in evaluating the effectiveness of intervention strategies. Furthermore, it also provides a clearer picture to investors and stakeholders about the long-term carbon liabilities associated with different commodities, aiding in more informed investment and operational decisions[54].

Both methods complement each other and provide a comprehensive understanding of the deforestation and carbon emissions landscape, helping to prioritise commodities and regions for targeted climate change mitigation efforts.

B. Supplementary Methods

1. Forest plantation mask

In our deforestation attribution, we filter out the tree cover loss over managed forests (i.e., both planted and plantation forests; see definition at ref.[55]), aiming to solely include the loss of natural forests. Since the global forest change dataset[3] does not differentiate between natural and managed forests, recognising any woody vegetation over 5m in height in a pixel as forested land, the signal from forest loss contains both removal of tree stands in natural forests (i.e., deforestation) and managed forests (due to logging/rotation harvesting in already established timber or oil palm plantation regions). To refine our analysis to only include deforestation, we exclude changes in tree cover associated with the management activities of planted and plantation forests established before 2001.

For datasets with annual updates, such as MapBiomas[56] and the oil palm extent in Indonesia[57], which documents land use since 2000 or earlier, we can readily discern whether tree cover losses occur in natural or managed forests. For those without such temporal land-use detail, we employ a forest plantation mask based on Du et al.[58] and Lesiv et al.[8] to identify and exclude managed forests (Supplementary Fig. 5). Du et al.[58] use the Spatial Database of Planted Trees (SDPT version 1.0[59]) – which is stated to cover nearly 82% of plantation forests globally – and time-series of Landsat satellite data

(from 1982-2020) to detect when these plantations in a pixel were first established (referred to as 'start year'). For our deforestation attribution, we only included forest plantations established after the year 2000 (i.e., start year > 2000), while tree cover loss in plantations established before 2000 was classified as rotational clearing. However, this approach carries the risk of overestimating deforestation for plantations with rotation periods exceeding 20 years, as these plantations may have been established before the timeframe analysed in Du et al.[58]. Conversely, Lesiv et al.[8] offer a global perspective on managed forests using more recent satellite imagery (2014-2016) and expert classification.

When pixels corresponding to forest plantations or tree crops (e.g., oil palm, coconut, and cocoa), those lacking a land-use record for the year 2000, intersect with the forest plantation mask (Supplementary Fig. 5), we consider these pixels to have been established pre-2001 and exclude them from our deforestation attribution analysis. We give precedence to Du et al.[58] plantation mask due to its comprehensive temporal coverage, which allows us to distinguish between natural and managed forest cover changes before and after the year 2000. In regions without coverage from Du et al.[58], such as Canada and Russia, we defer to Lesiv et al.[8] plantation mask. The latter case, however, may lead to conservative estimates of deforestation where plantation expansion occurred between 2001-2016 (since Lesiv et al.[8] is defined using remote sensing data from 2014-16), but the impact on our overall results is deemed minimal given the breadth of the SDPT database[59]. This masking is selectively applied to forest plantation and tree crop commodities; temporary crop and pasture commodities, typically non-woody and less likely to replace forest plantations, are not subjected to this masking.

2. Processing temporally explicit and temporally aggregated spatial datasets

We process temporally explicit datasets, such as MapBiomas and Soybeans, which provide yearly spatial extents from 2000 to 2022, unlike those that are temporally aggregated. Temporally explicit datasets facilitate direct attribution of deforestation to particular land-uses or commodities. We process them by applying a four-year moving window (i.e., a maximum three-year delay) from the year in which forest loss is detected. This window helps compensate for any delays between the observed forest loss and the actual conversion of that deforested land to agricultural land use. For instance, if a pixel shows forest loss in 2001 and is later identified as cropland in 2003 by MapBiomas, we attribute that forest loss to cropland. In cases where multiple land-use changes occur within the window, we prioritise the assignment in the order of forest plantations, woody perennial crops, pastures, herbaceous perennial and temporary crops (thus prioritising land-uses with higher rotation period over lower[60,61]).

Conversely, datasets that aggregate estimates over time pose challenges in pinpointing the immediate cause of deforestation, as they may not capture sequential land-use changes. Consider the cocoa plantations dataset as an example[21], which consolidates satellite data from 2018 to 2021 to create a cocoa plantation map for a single reference year. Suppose a forest loss occurred in a specific pixel in 2003, and that pixel overlaps with the cocoa plantation extent. In the absence of intervening land use data from 2003 to 2017, there is a risk of identifying or misidentifying cocoa as the deforestation driver if land use has changed during those intervening years. Thus, here, we follow a simplistic approach by aligning these temporally aggregated datasets with the year of forest loss when spatial overlap occurs (i.e., simply assuming that the land use that is eventually identified represents the proximate cause of deforestation). However, the attribution of forest loss does not extend beyond the final year of the remote sensing dataset used for the development of the spatial dataset (e.g., spatial attribution for cocoa beans in Côte d'Ivoire and Ghana does not go beyond 2021, and for sugarcane in Brazil, it does not go beyond 2019; see Supplementary Table 2).

3. Statistical land-use attribution

3.1 Estimating gross land-use expansion

We start the first step of this statistical attribution by estimating the expansion of croplands (*CLE*), permanent pastures (*PPE*), and forest plantations (*FPE*) over a three-year time lag following the observed year of forest loss (*t*), such that *lag* = min {3, 2021 – *t*} (Supplementary equations (1)-(3); Supplementary Fig. 1; 2021 is the last year of FAOSTAT data). The duration of this lag period is set to three years, reflecting empirical data on the typical interval between the initial forest clearing and the subsequent establishment of agricultural land for production[62,63]. This time-lagged approach is integral to synchronising the observed changes in land cover with the likely temporal dynamics of land-use development.

$$CLE_t = \max\left\{\frac{\left(CL_{t+lag} - CL_t\right) + \sum_{t}^{t+lag} Crop\ loss_t}{lag} - GPL_t, 0\right\};\ GPL_t = \max\left\{\min\left\{\frac{\left(PP_{t+lag} - PP_t\right)}{lag}, \frac{\sum_{t}^{t+lag} Grass\ loss_t}{lag}\right\}, 0\right\} \quad (1)$$

$$PPE_t = \max\left\{\frac{\left(PP_{t+lag} - PP_t\right) + \sum_{t}^{t+lag} Grassloss_t}{lag}, 0\right\} \quad (2)$$

$$FPE_t = \max\left\{\frac{FP_{t+lag} - FP_t}{lag}, 0\right\} \quad (3)$$

Here CL_t, PP_t, FP_t quantify the extent of croplands, permanent pastures, and forest plantations for a given year *t*, respectively. The land-use extent data for croplands and permanent pastures are sourced from FAOSTAT[12] (Supplementary equation (1)-(2)), while information on forest plantations is obtained from the FRA[55] (Supplementary equation (3)). Our analysis is focused on gross land-use change; hence, we enhance the net expansion figures from FAOSTAT and FRA with estimates of crop and pasture loss. These losses are computed using methodologies from Li et al.[64], which utilise a time series of the ESA CCI land cover dataset[65] (2000-2022) to track changes in crop and grass areas (i.e., proxy for pasture loss area).

Acknowledging the frequent expansions of croplands over pastures, as evidenced by remote sensing studies[66], we adjust our cropland expansion (CLE_t) calculations by deducting the gross pasture loss (GPL_t) (Supplementary equation (1)). This reflects the tendency for croplands to expand initially into pasture areas before encroaching on forested lands. This displaces cattle ranching into forest frontiers due to cropland expansion[67,68], leading us to correlate pasture expansion directly with forest loss (Supplementary equation (2)). In contrast, for forest plantations, we account only for the net change, as data on gross plantation loss is not available. Consequently, the expansion of forest plantations is directly linked to forest loss (Supplementary equation (3)).

3.2 Handling land-use mosaics

When faced with multi-land-use mosaics (specifically for MapBiomas[56], Curtis et al.[69] dominant driver dataset, and unclassified forest loss) that blend croplands, pastures, or forest plantations without clear demarcation, we distribute the area of forest loss within these mosaics (FL_{mosaic}) in proportion to the extent of each land use relative to the total observed expansion of land use (Supplementary equation (4)-(6); Supplementary Fig. 1). This means that the mosaic of cropland, pasture, and forest plantation is divided among them based on their respective contributions to overall land use expansion (i.e., the sum of CLE_t, PPE_t and FPE_t) (Supplementary equation (4)-(6)). In scenarios where the mosaic is solely composed of cropland and pasture (presently only MapBiomas[56]), we allocate the area between these two categories proportionately, with the combined extent of CLE_t and PPE_t – informing the total area used for this allocation.

$$FL_{CL,statistical,t} = \underset{(certain)}{FL_{mosaic,t}} \times \frac{CLE_t}{CLE_t + PPE_t + FPE_t} \quad \text{or} \quad \min\left\{ \max\left\{ CLE_t - FL_{CL,spatial,t}, 0 \right\}, \underset{(uncertain)}{FL_{mosaic,t}} \times \frac{CLE_t}{CLE_t + PPE_t + FPE_t} \right\} \quad (4)$$

$$FL_{PP,statistical,t} = \underset{(certain)}{FL_{mosaic,t}} \times \frac{PPE_t}{CLE_t + PPE_t + FPE_t} \quad \text{or} \quad \min\left\{ \max\left\{ PPE_t - FL_{PP,spatial,t}, 0 \right\}, \underset{(uncertain)}{FL_{mosaic,t}} \times \frac{PPE_t}{CLE_t + PPE_t + FPE_t} \right\} \quad (5)$$

$$FL'_{FP,statistical,t} = \underset{(certain)}{FL_{mosaic,t}} \times \frac{FPE_t}{CLE_t + PPE_t + FPE_t} \quad \text{or} \quad \min\left\{ \max\left\{ FPE_t - FL_{FP,spatial,t}, 0 \right\}, \underset{(uncertain)}{FL_{mosaic,t}} \times \frac{FPE_t}{CLE_t + PPE_t + FPE_t} \right\} \quad (6)$$

In this framework, mosaics are also divided into 'certain' and 'uncertain' categories. 'Certain' mosaics are those where the dataset confidently identifies the type of land use within the mosaics. For instance, MapBiomas[56] mosaics are certain that the mosaic land use is either cropland or pasture. Conversely, 'uncertain' mosaics, specifically those from the Curtis et al.[70] dataset, suggest probable land uses solely based on the predominant cause of forest loss over space and time, which may not always accurately reflect direct drivers of forest loss (since aggregated in a 10-km pixel over the full time period). This also encompasses unclassified forest loss as well, given that the driver of such forest loss cannot be associated with a specific land use. We impose a limit for these ambiguous cases (i.e., uncertain mosaics) (Supplementary equation (4)-(6) on the right). This constrains the categorisation of forest loss to whichever is smaller: the expansion of land-use categories minus the spatially attributed forest loss or the forest loss proportionally assessed based on relative land-use expansions – to avoid overestimating forest loss due to agriculture.

3.3 Capping deforestation due to forestry activities

Additionally, despite using a forest plantation mask, certain areas might inaccurately identify themselves as forest loss within natural forest, when in reality, they represent rotational clearing. This misclassification is particularly prevalent when tree cover loss pixels coincide with areas identified by Curtis et al.[70] as dominated by forestry activities ($FL_{forestry,spatial,t}$), stemming from challenges in differentiating between natural and managed forest losses. This issue is especially notable in countries like Sweden, Canada, and Russia, where extensively managed forest areas are not categorised as plantation forests according to FAO's definitions[71]. To counter potential overestimation of deforestation driven by forestry activities, our methodology enforces a cap on the statistical accounting of forest loss attributed to forest plantations ($FL_{FP,statistical,t}$). This cap ensures that the reported forest loss does not surpass the forest plantation expansion estimates provided by the FRA (i.e., FPE_t; Supplementary equation (7)).

$$FL_{FP,statistical,t} = \begin{cases} FL'_{FP,statistical,t} & \text{if } FL_{forestry,spatial,t} > 0 \text{ and } FPE_t \leq FL_{FP,spatial,t} + FL'_{FP,statistical,t} \\ \min\left\{ FPE_t - FL_{FP,spatial,t}, FL_{forestry,spatial,t} + FL'_{FP,statistical,t} \right\} & \text{if } FL_{forestry,spatial,t} > 0 \text{ and } FPE_t > FL_{FP,spatial,t} + FL'_{FP,statistical,t} \end{cases} \quad (7)$$

3.4 Gap filling

It should be noted that FAOSTAT provides land-use data up to the year 2021, which allows us to compute land-use expansion until 2020 (Supplementary equation (4)-(6)). To gap-fill for expansions in 2021 and 2022, we average the land use expansion from the preceding three years (i.e., 2018-2020) and then adjust it proportionally to the forest loss to estimates of 2021 and 2022 (Supplementary equation (8)).

$$CLE_t = \min\left\{\overline{\sum_{i=t-3}^{t-1} CLE_i},\ \overline{\sum_{i=t-3}^{t-1} CLE_i} \times \frac{FL_t}{\sum_{i=t-3}^{t-1} FL_{CL,i}}\right\} \qquad PPE_t = \min\left\{\overline{\sum_{i=t-3}^{t-1} PPE_i},\ \overline{\sum_{i=t-3}^{t-1} PPE_i} \times \frac{FL_t}{\sum_{i=t-3}^{t-1} FL_{PP,i}}\right\}$$

$$FPE_t = \min\left\{\overline{\sum_{i=t-3}^{t-1} FPE_i},\ \overline{\sum_{i=t-3}^{t-1} FPE_i} \times \frac{FL_t}{\sum_{i=t-3}^{t-1} FL_{FP,i}}\right\} \quad (8)$$

4. Statistical commodity attribution

4.1 Deforestation attributed to crop commodities

In the second-step of statistical attribution (Supplementary Fig. 1), we allocate total forest loss induced by cropland expansion ($FL_{CL,t}$, which is the sum of deforestation attributed to croplands spatially and statistically) to various crop commodities ($FL_{CL,statistical,i,t}$, where *i* refers to individual commodities). After excluding forest loss due to commodities already accounted for spatially ($\sum_i FL_{CL,spatial,i,t}$),the statistical land-use attribution step (Supplementary equation (9)) allocates cropland-driven deforestation proportionally to the expansion of each crop commodity ($CLE_{i,t}$) relative to the total expansion at the country level ($\sum_i CLE_{i,t}$). We use FAOSTAT's country scale 'crops and livestock products' statistics ($CL_{i,t}$) to estimate these expansions[12], maintaining the methodology and lag used previously (Supplementary equation (10)). The only exception is Brazil, where we use municipality-level (i.e., second-level administrative boundary) data from the Brazilian Institute of Geography and Statistics (IBGE)[13]. Notably, IBGE also estimates harvested areas for certain crops – specifically maize, groundnuts, potatoes, and beans – that are planted multiple times annually. To prevent double or triple counting of the deforestation attributable to these crops, we only use their first harvested area estimates rather than the total cumulative harvested area over the year. We note that currently, our focus is limited to Brazil due to the lack of available sub-national statistics in other countries. However, we anticipate incorporating these statistics in the future, as higher-quality data becomes available (see Supplementary Note 6).

If FAOSTAT or IBGE's total crop expansion ($\sum_i CLE_{i,t}$) exceeds the forest loss attributed to cropland ($FL_{CL,t}$; Supplementary equation (1)), we use the lower value between the two (Supplementary equation (9)). Additionally, any surplus ($FL_{CL,surplus,t}$) is apportioned among commodities based on their annual harvested areas, preserving proportionality and reflecting possible land-use changes (Supplementary equation (11)-(12)).

$$FL_{CL,statistical,i,t} = \left(\left(\max\left\{\min\left\{FL_{CL,t},\ \sum_i CLE_{i,t}\right\} - \sum_i FL_{CL,spatial,i,t}, 0\right\}\right) \times \frac{CLE_{i,t}}{\left(\sum_i CLE_{i,t} - \sum_j CLE_{j,t}\right)}\right) + FL_{CL,surplus,i,t} \quad (9)$$

$$CLE_{i,t} = \max\left\{\frac{CL_{i,t+lag} - CL_{i,t}}{lag}, 0\right\} \quad (10)$$

$$FL_{CL,surplus,t} = FL_{CL,t} - \left(\max\left\{\min\left\{FL_{CL,t},\ \sum_i CLE_{i,t}\right\} - \sum_j FL_{CL,spatial,i,t}, 0\right\}\right) - \sum_j FL_{CL,spatial,i,t} \qquad if\ \ FL_{CL,t} > \sum_i CLE_{i,t} \quad (11)$$

$$FL_{CL,surplus,i,t} = FL_{CL,surplus,t} \times \frac{CL_{i,t}}{\sum_i CL_{i,t}} \quad (12)$$

Here, $\sum FL_{CL,spatial,i,t}$ is the sum of all spatially attributed forest loss commodities. Since we prioritise deforestation estimated through remote sensing data over agricultural statistics, spatially attributed commodities with a score greater than 0.85 are excluded from statistical attribution. This threshold indicates a high confidence in the data reflecting the true extent of deforestation by that commodity, such as soybeans in South America and oil palm in Indonesia (scores for all datasets are mentioned in Supplementary Table 6, with the scoring methodology outlined in the 'Quality assessment' section). To compensate for this exclusion, we adjust the total crop commodity expansion by deducting $\sum_j CLE_{j,t}$ (i.e., the sum of harvested areas of commodities scoring above 0.85 or $FL_{CL,spatial,i,t} > CLE_{i,t}$) from $\sum_i CLE_{i,t}$ (Supplementary equation (10)). Additionally, as FAOSTAT provides harvest area data up to 2021, enabling commodity-driven expansions calculation up to 2020, we apply a similar methodology as before gap-fill for the year 2021 and 2022 (Supplementary equations (4)-(6)).

4.2 Deforestation attributed to pasture commodities

In the case of deforestation attributed to pastures ($FL_{PP,t}$), we attribute these losses to just two commodities: cattle meat and leather at 95% and 5% of the total deforested area, respectively, based on an economic allocation logic[38]. Although some studies have utilised weighted cattle density[72] data to minimise the inclusion of pastures used for other grazing livestock (e.g., sheep, camels, goats and horses)[43] and associated products (e.g., dairy), considerable uncertainties remain[17,73]. For instance, in some regions, the impact on pastoral communities could be considerable[74,75], however, the traditional land use and grazing patterns of these communities may diverge from what is detectable through satellite imagery or fit within formal land-use classifications. Moreover, the variability in cattle density over time poses a challenge, and therefore, is difficult to capture with datasets aggregated temporally, which might lead to under- or over-estimation of cattle meat-driven deforestation. As a result, we adopted an approach grounded in economic-allocation logic to attribute commodities to pastures[38].

4.3 Deforestation attributed to forestry commodities

Forest loss attributed to forest plantations ($FL_{FP,t}$) is categorised as 'Forest plantation (Unclassified)', unless the specific species of the plantations can be spatially attributed using the global plantation dataset[58]. In these cases, where the species information is available, the forest plantation is referred to as 'Forest plantation (*species name*)'.

5. Peatland drainage emissions

Peatland emissions can continue for many years, even decades, after initial land-use change due to the ongoing oxidation of organic carbon in the peat[76]. Assessing emissions from peatland drainage is difficult due to uncertainties in peat subsidence, which can vary with local conditions and management practices[77]. This variability, alongside the inherent challenges in measuring peatland emissions due to the dynamic nature of peat decomposition and water table fluctuations, complicates the accuracy of such estimates[76].

Unlike other deforestation emissions (AGB, BGB, etc.), which are considered locked-in or committed, the continuous emission profile of peatland emissions necessitates annual emission accounting to accurately

reflect their ongoing impact. Furthermore, international frameworks such as the IPCC guidelines[51] require countries to report their peatland emissions annually, which aligns with our approach to reporting peatland emissions.

Of the literature used for estimating peatland drainage emission factors[51,77–79], the factors from ref.[79] are based on the IPCC Wetland supplement[51]. For forest plantations, we prioritise the values from ref.[78], resorting to the IPCC values[51] only when ref.[78] does not provide the necessary emission factors. The ref.[78] indicates that the IPCC values for peatlands in tropical and boreal forestry regions are substantially lower in magnitude. They suggest that emission factors for forestry on drained organic soils provided by the IPCC are based on a limited number of measurements, often using trenching or the eddy covariance technique. These techniques might not fully capture the ongoing carbon emissions, especially for below-ground litter input, which can be substantial in peatlands.

6. Quality assessment

6.1 Scoring metric justification

Since the datasets used in deforestation attribution vary in spatio-temporal granularity (or resolutions) and explicitness (e.g., some datasets provide only land-use information while others capture the spatial extent of commodities), they differ in their ability to actually capture deforestation due to commodity production. The scoring metric normalises the scope of all datasets, making them comparable and allowing for a consistent assessment of the reliability of deforestation estimates.

For instance, a spatial dataset for cropland and oil palm may both exhibit 90% overall accuracy (OA), but their precision in pinpointing oil palm-induced deforestation differs substantially. This difference arises because spatial data on oil palm is explicitly designed to identify areas where oil palm is grown, making it more suitable for linking deforestation specifically to oil palm plantations (dLUC). In contrast, cropland spatial data only indicates that a crop commodity is leading to deforestation without explicitly identifying the commodity-specific driver. In the latter case, assessing the commodity's impact will require using agricultural statistics (Extended Data Fig. 1) to help associate the deforestation likely driven by oil palm (sLUC) with overall deforestation estimates resulting from cropland expansion. Therefore, a higher accuracy spatial dataset does not necessarily equate to a more reliable deforestation estimate.

Similarly, two oil palm datasets with the same temporal resolution and overall accuracy but varying spatial resolution will differ in their capacity to attribute deforestation accurately at a 30-meter pixel scale. The scoring metric adjusts the overall accuracy (OA_j; equation (2)) to account for differences in spatial, temporal, and explicitness aspects, thereby providing a nuanced understanding of the reliability of deforestation estimates produced by the DeDuCE model.

6.2 Calculation of Integrated Quality Index (IQI)

Examples of when deforestation estimates are calculated using only the spatial commodity datasets

Soya beans – Bolivia (2015)
Deforestation: 20840.45 ha

Only one dataset contributed to deforestation estimates:

1. Song et al.[20]-Soya beans: 20840.45 ha
   (OA = 0.95; Score = 0.93)

Oil palm fruit – Indonesia (2016)
Deforestation: 261034.13 ha

More than one dataset contributed to deforestation estimates (note that the spatial attributions from the datasets below are non-overlapping):

1. MapBiomas[56]-Oil palm fruit: 5904.05 ha

$$\textbf{IQI} = \frac{(20840.45 \times 0.95 \times 0.93)}{20840.45} = \mathbf{0.88}$$

(OA = 0.85; Score = 0.83)

2. Descals et al.[80]-Oil palm fruit: 2883.93 ha (OA = 0.9852; Score = 0.72)
3. Gaveau et al.[57]-Oil palm fruit: 252246.15 ha (OA = 0.956; Score = 1)

$$\textbf{IQI} = \frac{(5904.05 \times 0.85 \times 0.83) + (2883.93 \times 0.9852 \times 0.72) + (252246.15 \times 0.956 \times 1)}{261034.13} = \mathbf{0.95}$$

Example of when deforestation estimates are calculated using spatial land-use data and agricultural statistics

Sugar cane – Belize (2014)
Deforestation: 3031.61 ha

Agriculture statistics (see Supplementary Table 12):

1. *Flag*$_{Land\ use}$ = E
2. *Flag*$_{Production}$ = A

Multiple land-use datasets that contributed to the aggregation of deforestation estimates:

1. Potapov et al.[81]-Cropland (post-statistical attribution): 2876.96 ha (OA = 0.9735; Score = 0.65)
2. Curtis et al.[70]-Dominant driver (post-statistical attribution): 154.65 ha (OA = 0.89; Score = 0.40)

Modified OA with agricultural flags (see equation (2) and Supplementary Table 6):

$$OA = OA_j \times \left(\frac{0.80 + 1}{2} - \frac{0.5}{3} \right) = 0.73$$

$$\textbf{IQI} = \frac{(2876.96 \times 0.9735 \times 0.65) + (154.65 \times 0.89 \times 0.40)}{3031.61} \times 0.73 = \mathbf{0.45}$$

Example of when deforestation estimates are primarily calculated using good-quality agricultural statistics

Wheat – Kazakhstan (2006)
Deforestation: 717.05 ha

Agriculture statistics (see Supplementary Table 12):

1. *Flag*$_{Land\ use}$ = A
2. *Flag*$_{Production}$ = A

Multiple land-use datasets that contributed to the aggregation of deforestation estimates:

1. Potapov et al.[81]-Cropland (post-statistical attribution): 17.76 ha (OA = 0.9735; Score = 0.65)
2. Curtis et al.[70]-Dominant driver (post-statistical attribution): 0.08 ha (OA = 0.89; Score = 0.40)
3. Hansen et al.[3]-Tree cover loss (post-statistical attribution): 699.21 ha (OA = 0.996; Score = 0.53)

Modified OA with agricultural flags (see equation (2) and Supplementary Table 6):

$$OA = OA_j \times \left(\frac{1+1}{2} - \frac{0.5}{3} \right) = 0.83$$

$$\textbf{IQI} = \frac{(17.76 \times 0.9735 \times 0.65) + (0.08 \times 0.89 \times 0.40) + (699.21 \times 0.996 \times 0.53)}{717.05} \times 0.83 = \textbf{0.44}$$

Example of when deforestation estimates are primarily calculated using poor-quality agricultural statistics

Rubber – Cambodia (2017)
Deforestation: 27419.11 ha

Agriculture statistics (see Supplementary Table 12):

1. $Flag_{Land\ use}$ = E
2. $Flag_{Production}$ = T

Multiple land-use datasets that contributed to the aggregation of deforestation estimates:

1. Potapov et al.[81]-Cropland (post-statistical attribution): 4297.33 ha (OA = 0.9735; Score = 0.65)
2. Curtis et al.[70]-Dominant driver (post-statistical attribution): 23121.03 ha (OA = 0.89; Score = 0.40)
3. Du et al.[58]-Global Forest Plantation (directly classifies Rubber): 0.75 ha (OA = 0.7825; Score = 0.70)

Modified OA with agricultural flags (see equation (2) and Supplementary Table 6):

$$OA = OA_j \times \left(\frac{0.8+0.6}{2} - \frac{0.5}{3} \right) = 0.53$$

$$\textbf{IQI} = \frac{(4297.33 \times 0.9735 \times 0.65) + (23121.03 \times 0.89 \times 0.40)}{27419.11} \times 0.53 + \frac{(0.75 \times 0.7825 \times 0.70)}{27419.11} = \textbf{0.21}$$

C. Supplementary Figures

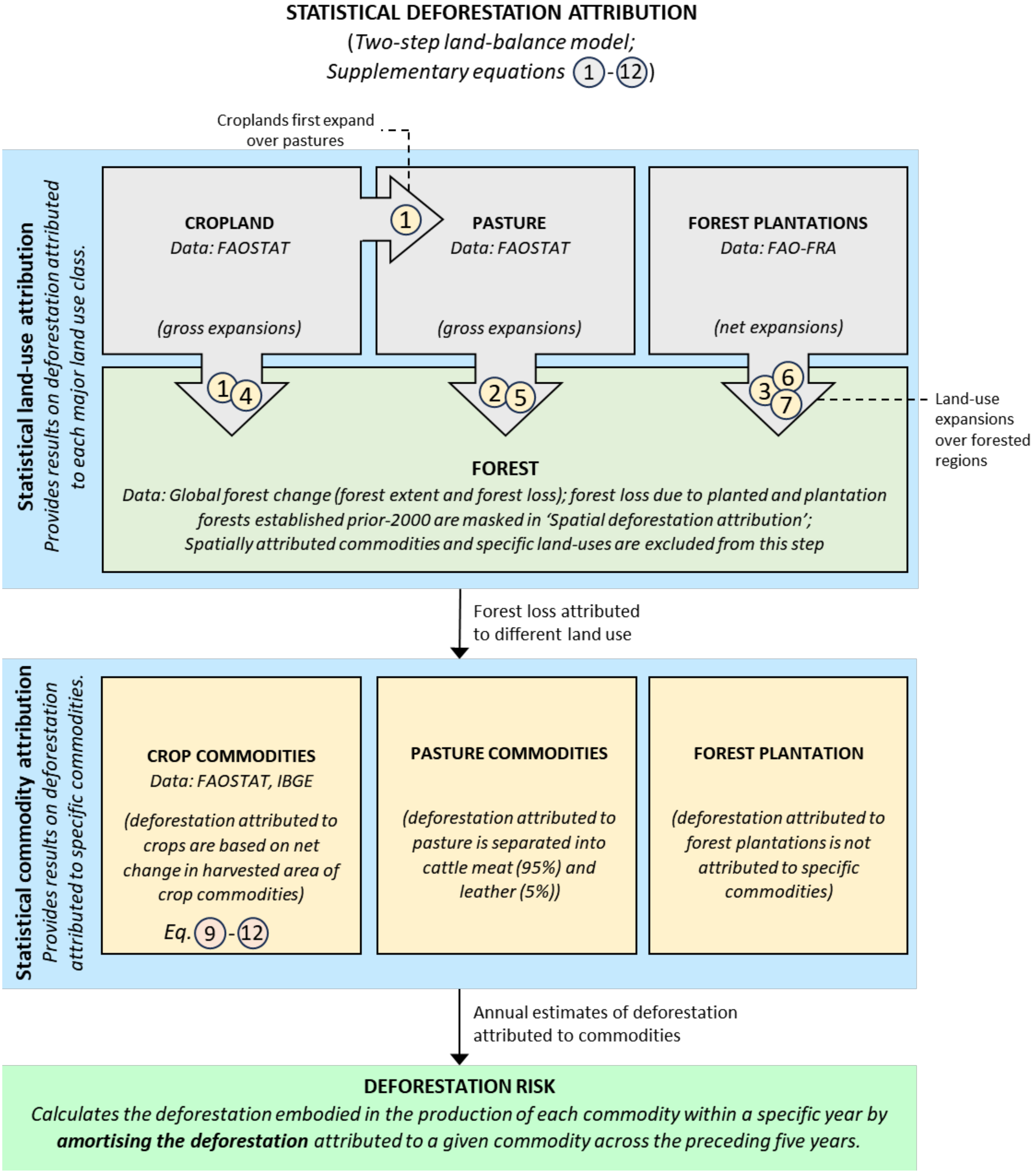

Supplementary Fig. 1 | Visual representation of the statistical deforestation attribution (i.e., two-step land balance model). The figure is adapted from ref.[67].

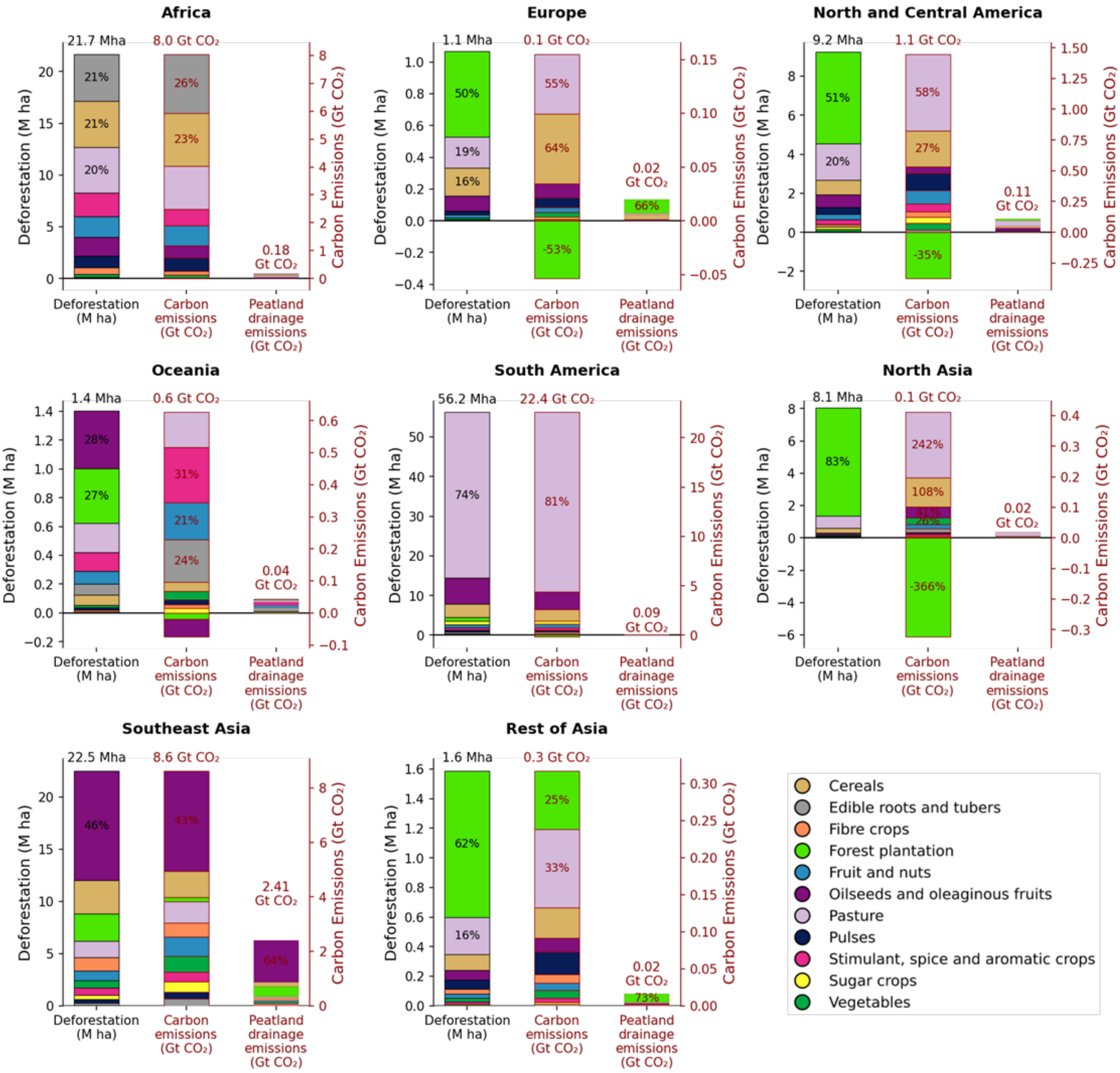

Supplementary Fig. 2 | Geographical overview of commodity-driven deforestation (2001-2022). Similar to Fig. 3b in the main text, this figure shows agriculture and forestry-driven deforestation and corresponding carbon emissions across but here broken down by different geographical regions. Total deforestation, carbon emissions and peatland drainage emissions for the period 2001-2022 are shown on the top on individual bars.

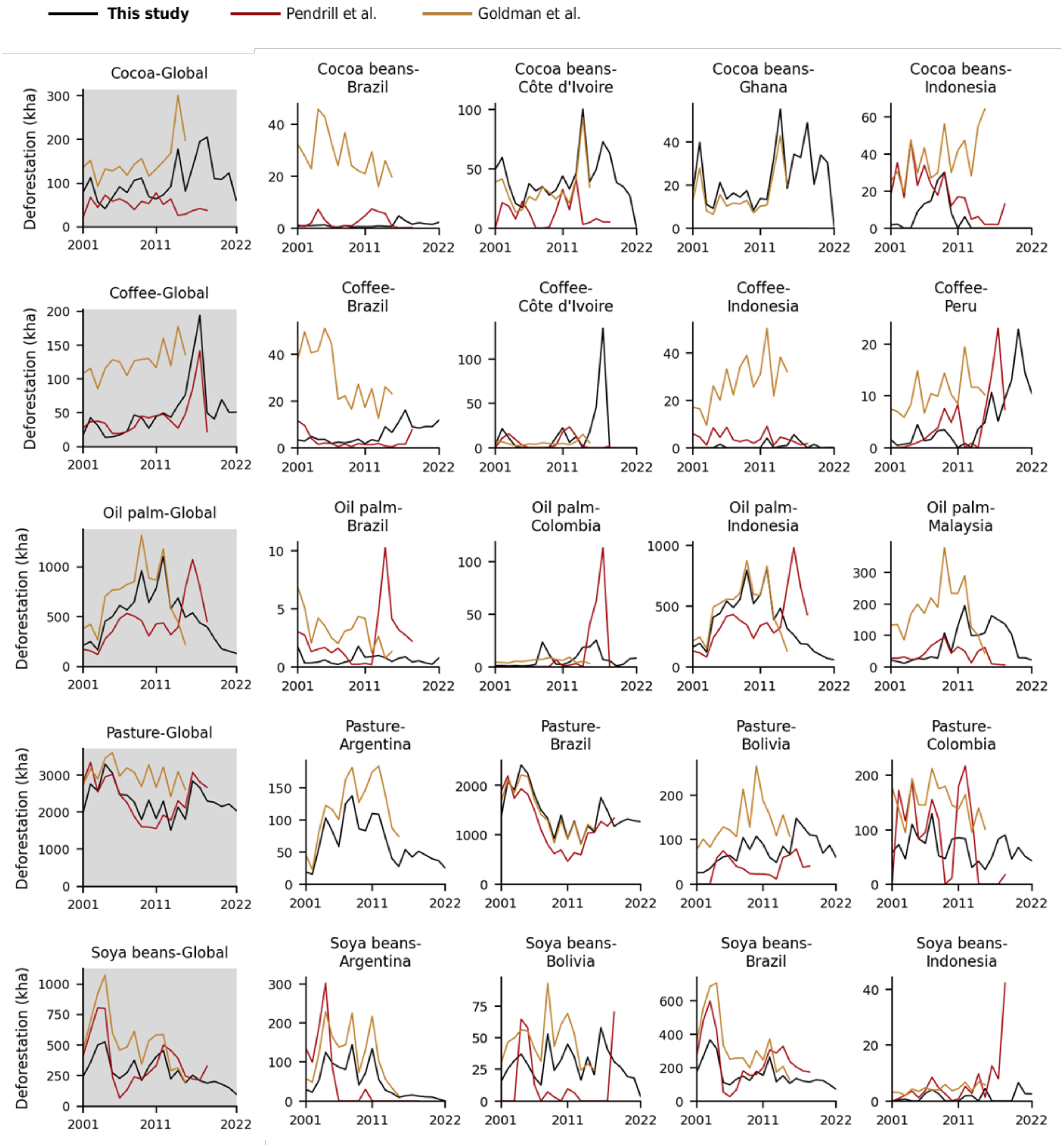

Supplementary Fig. 3 | Comparison of deforestation estimates of major deforestation-risk commodities and countries with other studies. Studies include estimates from Pendrill et al.[38] and Goldman et al.[43].

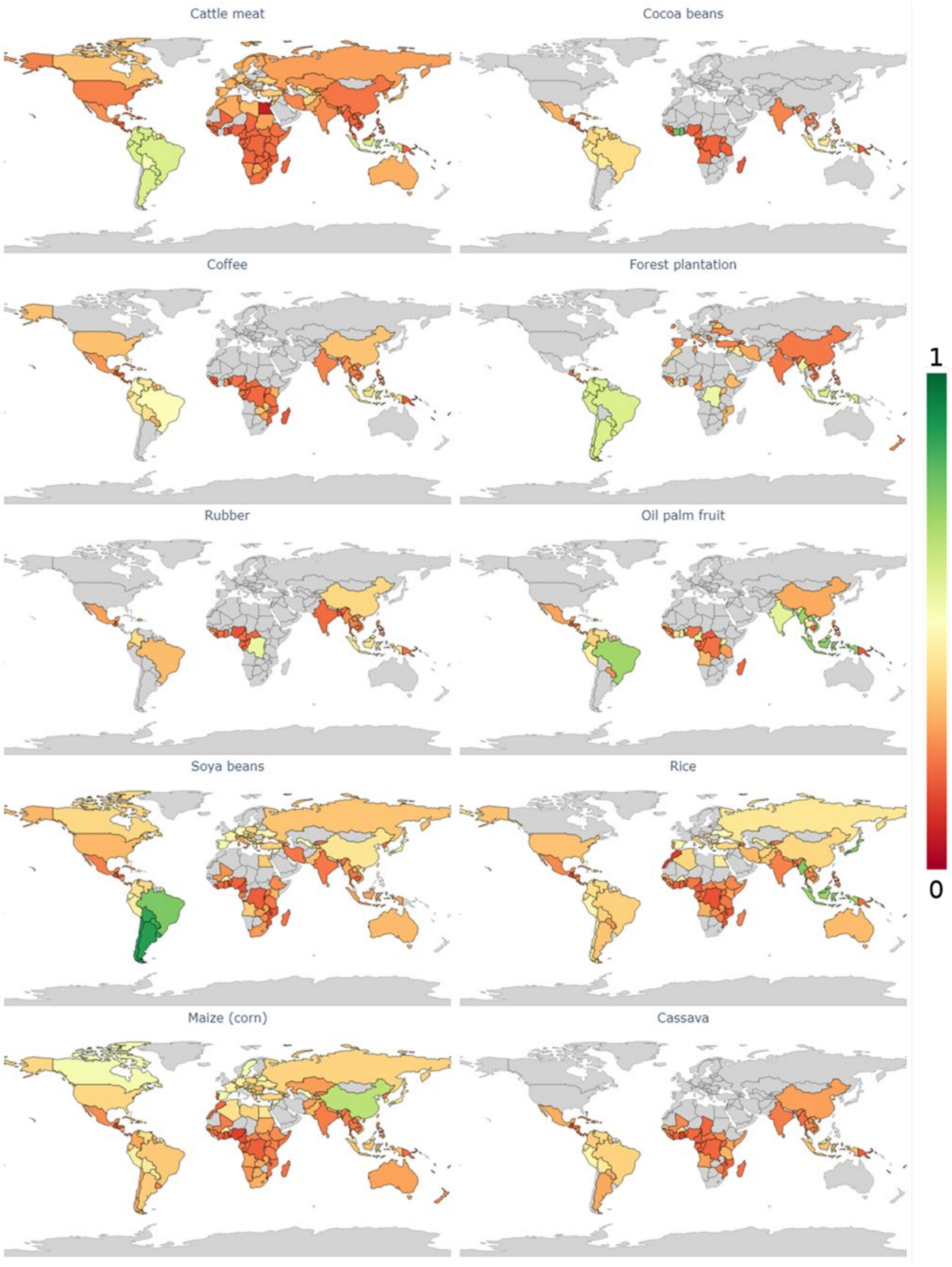

Supplementary Fig. 4 | Integrated Quality index (IQI) of major deforestation-risk commodities as shown in Fig. 4. The IQI above is weighted for estimates from 2018 to 2022. Here, higher values of the IQI indicate better quality of deforestation attribution.

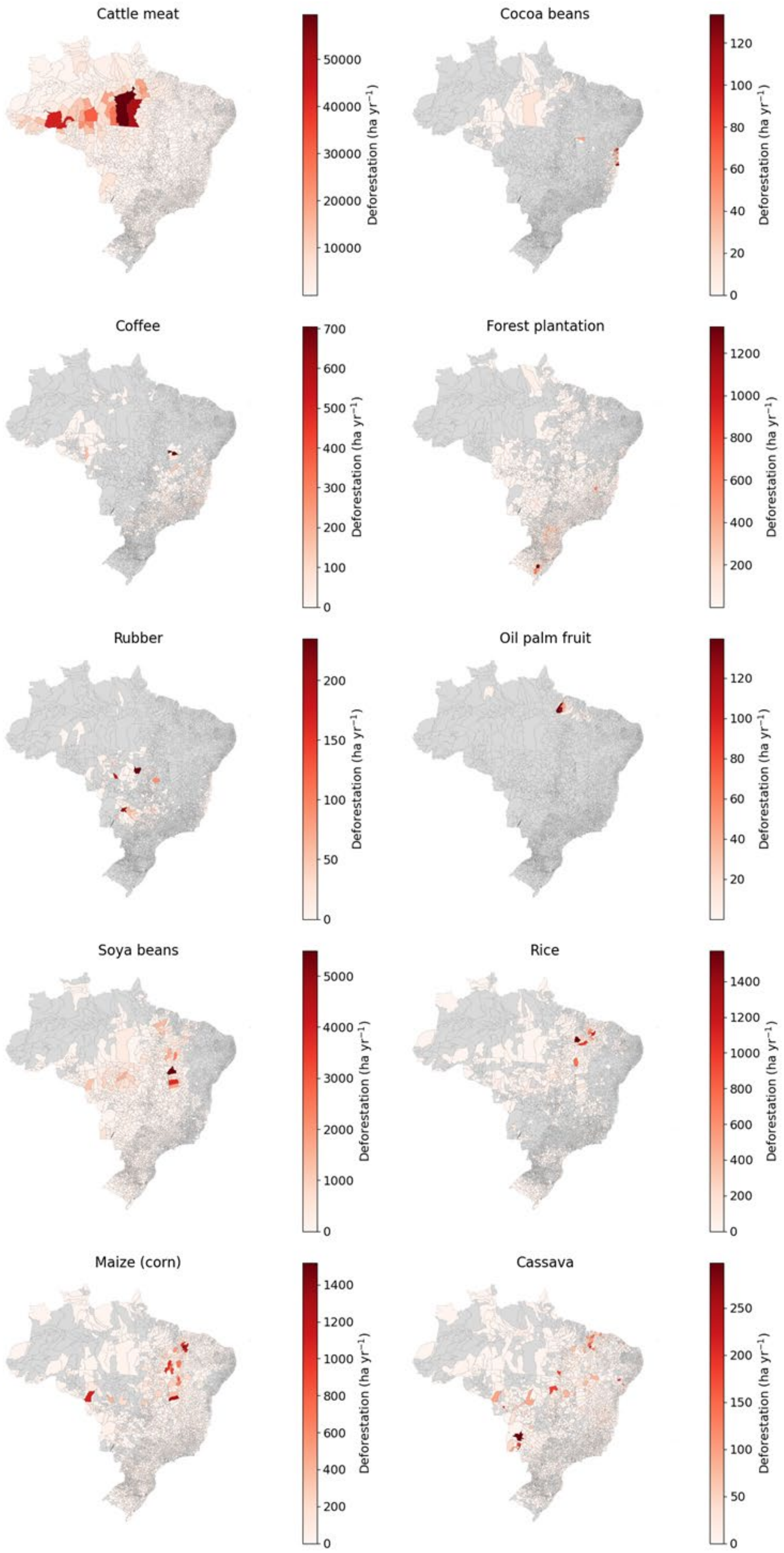

Supplementary Fig. 5 | Hotspots of major deforestation-risk commodities for Brazil (aggregated for 2018-2022).

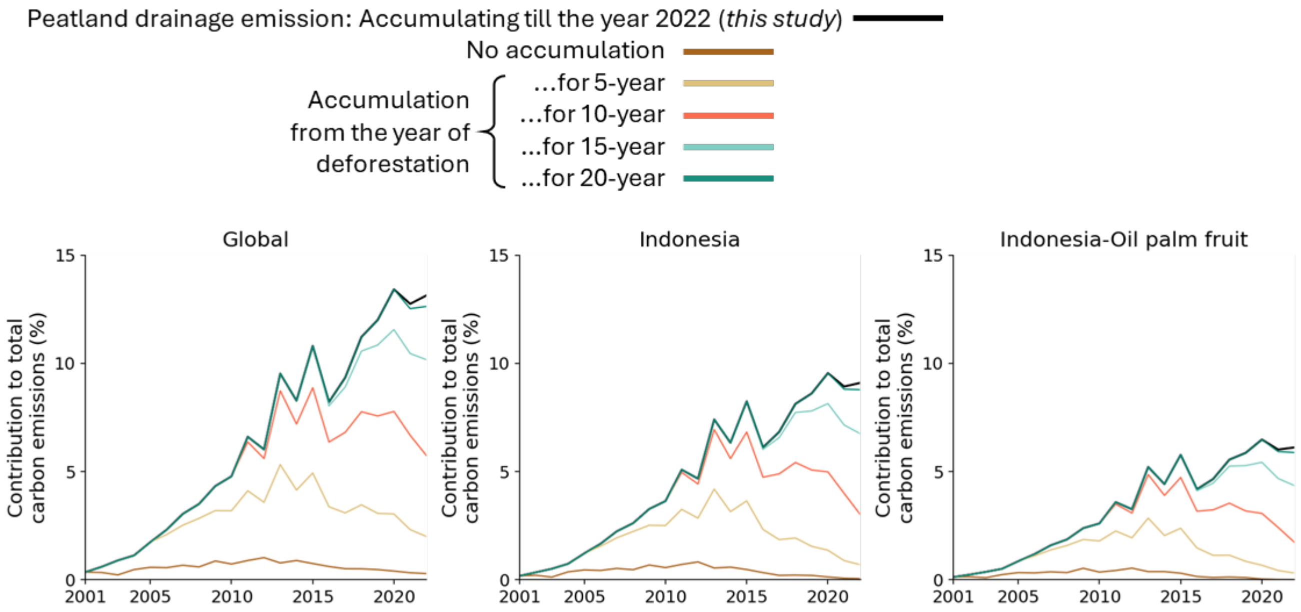

Supplementary Fig. 6 | Contribution of peatland drainage emissions to total deforestation carbon emissions. These contributions are based on different accumulation periods for peatland drainage emissions from the year of deforestation.

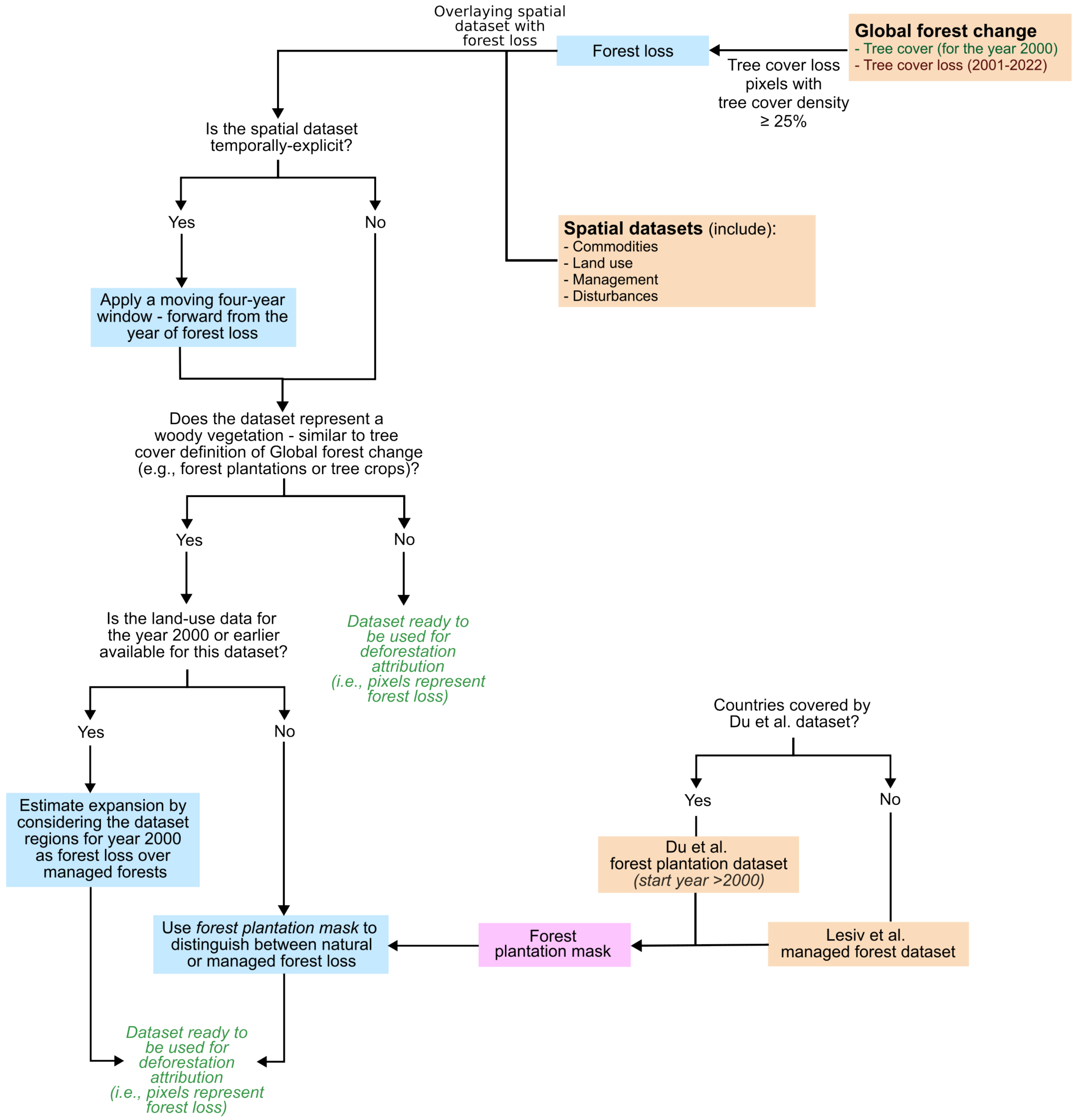

Supplementary Fig. 7 | Framework for distinguishing natural forest loss and loss over managed forests. Global forest plantation mask based on Du et al.[58] and Lesiv et al.[8].

D. Supplementary Tables

Supplementary Table 1 | Country and commodity groups with their respective deforestation-carbon emission estimates and integrated quality index (2001-2022). Note that the table below excludes countries and commodities that either experienced no deforestation or lacked FAOSTAT statistics for the period from 2001 to 2022. Absolute values are archived on Zenodo (see data availability).

**Continent/Country group (*number of countries in the group*)**	**Deforestation attribution, unamortized (ha)**	**Deforestation emissions excl. peat drainage, unamortized ($MtCO_2$)**	**Peatland drainage emissions ($MtCO_2$)**	**Weighted Integrated Quality Index (IQI)**
Africa (*53*)	21,681,260	8,040	178	0.29
Europe (*37*)	1,067,287	101	20	0.33
North and Central America (*22*)	9,236,562	1,071	108	0.28
Oceania (*8*)	1,402,479	553	44	0.25
South America (*14*)	56,244,470	22,436	88	0.60
North Asia (*8*)	8,068,203	88	19	0.28
Southeast Asia (*11*)	22,504,450	8,626	2,412	0.56
Rest of Asia (*26*)	1,589,387	317	17	0.28
**Total (179)**	**121,794,098**	**41,233**	**2,885**	

**Commodity group (*number of commodities in the group*)**	**Deforestation attribution, unamortized (ha)**	**Deforestation emissions excl. peat drainage, unamortized ($MtCO_2$)**	**Peatland drainage emissions ($MtCO_2$)**	**Weighted Integrated Quality Index (IQI)**
Cereals (*15*)	12,404,970	4,555	208	0.35
Edible roots and tubers with high starch (*7*)	5,392,571	2,754	75	0.27
Fibre crops (*15*)	2,336,180	761	89	0.31
Forest plantation (*16*)	16,989,600	-683	435	0.30
Fruit and nuts (*50*)	4,181,104	2,100	89	0.30
Oilseeds and oleaginous fruits (*22*)	20,086,950	5,942	1,595	0.74
Pasture (*2*)	51,058,210	21,593	199	0.53
Pulses - dried leguminous vegetables (*12*)	2,363,238	1,068	32	0.33
Stimulant, spice and aromatic crops (*16*)	4,016,254	1,426	79	0.49
Sugar crops (*3*)	1,534,438	767	20	0.52
Vegetables (*26*)	1,430,583	952	64	0.32
**Total (184)**	**121,794,098**	**41,233**	**2,885**	

Supplementary Table 2 | Datasets used in this study and their description.

Datasets	Spatial extent	Spatial resolution	Temporal resolution	References
**Datasets used for spatial deforestation attribution**				
Global forest change-v1.10: Tree cover (2000) and tree cover loss (2001-2022)	Global	30 m	2001-2022	3
Global plantation dataset* *(*Based on the spatial database of planted trees[59])*	Argentina, Australia, Brazil, Cambodia, Cameroon, Chile, China, Colombia, Costa Rica, Democratic Republic of the Congo, Ecuador, European countries, Gabon, Ghana, Guatemala, Honduras, India, Indonesia, Côte d'Ivoire, Japan, Kenya, Liberia, Malawi, Malaysia, Mexico, Myanmar, Nepal, New Zealand, Nicaragua, Nigeria, Pakistan, Panama, Papua New Guinea, Peru, Philippines, Rwanda, Solomon Islands, South Africa, South Korea, Sri Lanka, Thailand, Uruguay, United States, Venezuela, Vietnam	30 m	1982-2020	58
MapBiomas Collection	Argentina, Bolivia, Brazil, Chile, Colombia, Ecuador, French Guiana, Guyana, Paraguay, Peru, Suriname, Uruguay, Venezuela, Indonesia	30 m	2001-2022 (for all countries, except Bolivia); 2001-2021 (for Bolivia)	56
Croplands	Global	30 m	Aggregated temporally at every 4-year intervals between 2000-2019	81
Sugarcane	Brazil	30 m	Aggregated temporally using data for year 2016-2019	82
Soya beans	South America	30 m	2001-2022	20
Rice	Northeast and Southeast Asia	10 m	Aggregated temporally using the data for year 2017-2019	83
Rapeseed	Argentina, Europe, United States and Canada	10 m	Aggregated temporally using data for year 2017-2019	84
Maize (corn)	China	30 m	2001-2020	85
Cocoa	Côte d’Ivoire and Ghana	10 m	Aggregated temporally using data for year 2018-2021	21
Coconut	Pan-tropical	20 m	2020	86
Oil palm fruit	Indonesia	Vector	2000-2019	57
	Malaysia and Indonesia# (#not considered for Indonesia)	100 m	2001-2018	87
	Pan-tropical	10 m	2019	80
Forest loss due to fire	Global	30 m	2001-2022	88
Forest management	Global	100 m	Aggregated temporally using data for year 2014-2016	8
Dominant drivers of forest loss	Global	10 km	Aggregated temporally using data for year 2001-2022	70
**Datasets used for statistical deforestation attribution**				
FAOSTAT-Land use (values extracted for ‘Cropland’ and ‘Permanent meadows and pastures’)	Global	Aggregated at national level	1961-2021	12

FAOSTAT-Production	Global	Aggregated at national level	1961-2021	12
Forest Resource Assessment (FAO-FRA) (for forest plantation)	Global	Aggregated at national level	1990, 2000, 2010, 2015, 2016, 2017, 2018, 2019, 2020	55
Forestry statistics	Taiwan	Aggregated at national level	2000, 2005, 2010, 2015, 2020	89
Brazilian Institute of Geography and Statistics (IBGE)	Brazil	Aggregated at municipality level	1974-2022	13
Crop and grass loss	Global	300 m	1992-2020	64,65,90
**Datasets used for estimating carbon emissions**				
Aboveground biomass$ *($Used to estimate belowground biomass[91], deadwood and litter carbon stocks[92])*	Global	30 m	2000	92
Root-to-shoot biomass ratio	Global	1 km	Aggregated temporally using datasets from several years	93
Soil organic carbon stocks	Global	250 m	Aggregated temporally using datasets from several years	94
Peatland extentΦ *(ΦGlobally aggregated peatland extent is based on refs.[95–99])*	Global	30 m	Aggregated temporally using datasets from several years	100
Ecoregions	Global	Vector		101
Precipitation	Global	5 km	1981-2022	102
Elevation	Global	90 m		103
**Other datasets**				
Database of Global Administrative Areas-v4.1 (GADM)	Global	Vector		104

Supplementary Table 3 | Comparison of DeDuCE deforestation estimates with remote sensing datasets. Benchmarking the DeDuCE model against the best available evidence on commodity-driven deforestation derived from remote sensing data, as well as against a version of the model run without spatial data (beyond forest loss) and the Pendrill et al. dataset, which it extends and improves upon.

			**Other studies using spatial data**		**DeDuCE (spatial + statistical)**		**Non-spatial data**	
							**DeDuCE (statistical)**	**Pendrill et al.**[38]
**Commodity**	**Country/region**	**Time period**	**References**	**Total deforestation (kha)**	**Total deforestation (kha) & difference w.r.t reference (%)**	**Integrated Quality Index (IQI)**	**Total deforestation (kha) & difference w.r.t reference (%)**	
**Cattle meat**	Brazil	2001-2016	zu Ergmgassen et al. 2020[105]	15,941	22,792 (+43%)	0.59	10,246 (-36%)	**18,344 (+15%)**
		2004-2022[a]	Orbae[b]	19,482	25,099 (+29%)	0.59	10,249 (-47%)	**19,264 (-1%)**
	Brazil	2014-2018	Trase[c]	2,003	6,315 (+215%)	0.59	**2,959 (-48%)**	5,570 (+178%)
	Paraguay			1,220	**981 (-20%)**	0.59	72 (-94%)	123 (-90%)
	Africa	2001-2020[d]	Masolele et al. 2024[39]	1,713	**3,516 (+105%)**	0.21	7,095 (+314%)	9,345 (+446%)
	South America	2001-2005	de Sy et al. 2015[106]	15,310	**11,771 (-23%)**	0.59	1,821 (-85%)	10,950 (-28%)
**Cashew nuts**	Côte d'Ivoire	2001-2020[d]	Masolele et al. 2024[39]	329	**329 (0%)**	0.25	415 (+26%)	254 (-23%)
	Ghana			109	53 (-51%)	0.25	**67 (-38%)**	1 (-99%)
	Guinea			157	3 (-98%)	0.22	**6 (-96%)**	2 (-99%)
	Africa			1,343	510 (-62%)	0.21	**819 (-39%)**	421 (-69%)
**Cocoa beans**	Côte d'Ivoire	2001-2020[d]	Masolele et al. 2024[39]	957	**870 (-9%)**	0.79	638 (-33%)	238 (-75%)
		2003-2017	Trase[c]	1,353	**624 (-54%)**	0.79	586 (-57%)	187 (-86%)
	Ghana	2001-2019	Reiner et al. 2025[46]	354	**437 (+23%)**	0.79	174 (-83%)	0 (-100%)
		2001-2020[d]	Masolele et al. 2024[39]	445	**471 (+6%)**	0.79	174 (-61%)	0 (-100%)
		2003-2017	Trase[c]	1,032	**310 (-70%)**	0.79	109 (-89%)	0 (-100%)
	Côte d'Ivoire & Ghana	2001-2019[e]	Kalischek et al. 2023[21]	962	1,271 (+32%)	0.79	**812 (-16%)**	214 (-78%)
	Africa	2001-2020[d]	Masolele et al. 2024[39]	2,249	**1,606 (-29%)**	0.65	1,138 (-49%)	373 (-83%)
**Cotton**	United States	2004-2022	Orbae[b]	43	**69 (+61%)**	0.43	528 (+1128%)	–
**Maize (corn)**	China	2004-2022	Orbae[b]	12	**126 (+970%)**	0.75	710 (+5919%)	–
	United States			112	**81 (-28%)**	0.46	160 (+43%)	–
**Oil palm fruit**	Côte d'Ivoire	2001-2020[d]	Masolele et al. 2024[39]	81	**84 (+4%)**	0.52	44 (-46%)	17 (-79%)
	Ghana			33	55 (+66%)	0.52	**37 (+11%)**	3 (-90%)
	Indonesia	2001-2018	Gaveau et al. 2022[57]	8,346	7,445 (-11%)	0.95	**8,459 (+1%)**	6,707 (-20%)
	Liberia	2001-2020[d]	Masolele et al. 2024[39]	41	**13 (-67%)**	0.56	1 (-98%)	1 (-98%)

	Africa			356	418 (+18%)	0.36	**337 (-5%)**	126 (-65%)
**Soya beans**	Argentina	2014-2018	Trase[c]	49	**70 (+41%)**	0.88	10 (-81%)	0 (-100%)
	Bolivia	2015-2019		78	177 (+126%)	0.87	94 (+19%)	**88 (+12%)**
	Brazil	2014-2018		742	**621 (-16%)**	0.86	2,686 (+262%)	1,117 (+51%)
	Paraguay	2014-2018		37	**48 (+32%)**	0.88	49 (+8%)	100 (+122%)
	South America	2001-2016	Song et al. 2021[20]	3,275	**4,071 (+24%)**	0.87	6,597 (+101%)	4,686 (+43%)
	United States	2004-2022	Orbae[b]	77	**160 (+106%)**	0.43	1,502 (+1841%)	–
**Sugarcane**	Argentina	2004-2022[a]	Orbae[b]	20	**19 (-8%)**	0.38	33 (+65%)	6 (-70%)
	México			16	37 (+128%)	0.28	40 (+147%)	**14 (-13%)**
	United States			1	**2 (+42%)**	0.48	7 (+530%)	–
**Row crops**	Brazil	2001-2014	Zalles et al. 2019[107]	4,017	**3,557 (-11%)**	0.74	11,659 (+190%)	5,864 (+46%)
**Cropland**	South America	2001-2005	de Sy et al. 2015[106]	4,535	3,138 (-31%)	0.63	14,039 (+210%)	**5,241 (+16%)**

[a] As the Pendrill et al.[38] only has data up until the year 2018, the total deforestation in the period 2004-2018 is multiplied by 19/15 to provide a fair comparison to DeDuCE and Orbae[b] (i.e., for the period 2004-2022).
[b] Orbae[108] – by AdAstra Sustainability – quantifies land-use changes associated with (selective) agricultural commodities between 2004-2023. The total deforestation estimates are multiplied by 19/20 for it to be comparable to DeDuCE (i.e., for the period 2004-2022).
[c] Trase[42] – a global supply chain transparency platform – maps the flow of (selective) agricultural commodities linked to deforestation in global supply chain.
[d] As above, the Pendrill et al.[38] total deforestation in the period 2001-2018 is multiplied by 20/18 to provide a fair comparison to DeDuCE and Masolele et al.[39] (i.e., for the period 2001-2020).
[e] As above, the Pendrill et al.[38] total deforestation in the period 2001-2018 is multiplied by 19/18 to provide a fair comparison to DeDuCE and Kalischek et al.[21] (i.e., for the period 2001-2019).

Supplementary Table 4 | Summary of the datasets and models used for deforestation and carbon emission comparisons in Fig. 2. A comparison of deforestation estimates for major food commodities between this study, Pendrill et al., and Goldman et al. is presented in Supplementary Fig. 3.

Study or dataset	Brief methodology	Scope and comprehensiveness of the output	Accessibility, replicability and updates
DeDuCE model ***(present study)***	*Input*: Several remote sensing datasets and agricultural statistics (see Supplementary Table 2)<br>*Deforestation attribution model*: Hybrid (Spatial and statistical)<br>*Carbon emission accounting*: Hybrid (includes emission due to loss of AGB, BGB, deadwood, litter, SOC, and carbon stocks of replacing commodity)	*Spatial and temporal coverage:* Global (2001-2022)<br>*Spatial aggregation:* Deforestation and carbon emission estimates aggregated at national level (sub-national for Brazil)<br>*Comprehensiveness of estimates:* Commodity-level estimates	*Data availability:* Openly available (✓)<br>*Code for replicability:* Openly available (✓)<br>*Updated post-publication:* N.A.
Pendrill et al.[37]	*Input*: Spatial tree cover loss, agricultural statistics and AGB stocks<br>*Deforestation attribution model*: Statistical<br>*Carbon emission accounting*: Statistical (includes AGB, BGB, SOC and carbon stocks of replacing commodity)	*Spatial and temporal coverage:* Tropical countries (2001-2018)<br>*Spatial aggregation:* Deforestation and carbon emission estimates aggregated at national level (sub-national for Brazil and Indonesia)<br>*Comprehensiveness of estimates:* Commodity-level estimates	*Data availability:* ✓<br>*Code for replicability:* Not openly available (✗)<br>*Updated post-publication:* Yes (✓; now covers 2001-2018)
Goldman et al.[43]	*Input*: Spatial tree cover loss, commodity maps and dominant driver of forest loss<br>*Deforestation attribution model*: Spatial<br>*Carbon emission accounting*: Not estimated	*Spatial and temporal coverage:* Global, though spatial coverage limited to coverage of spatial datasets (2001-2015)<br>*Spatial aggregation:* Deforestation estimates aggregated at national level<br>*Comprehensiveness of estimates:* Commodity-level estimates for EUDR commodities (Oil palm, Soybeans, Cattle meat, Wood fibre, Cocoa beans, Coffee and Rubber)	*Data availability:* Can be requested from corresponding authors (✓)<br>*Code for replicability:* ✗<br>*Updated post-publication:* No (✗)
Hoang et al.[109] (uses Curtis et al.[70])	*Input*: Spatial tree cover loss, forest plantation mask and dominant drivers of forest loss<br>*Deforestation attribution model*: Spatial<br>*Carbon emission accounting*: Not estimated	*Spatial and temporal coverage:* Global (2001-2015); however, results only included G7 member counties, China, India, Brazil, Indonesia, Mexico, and remaining G20 countries<br>*Spatial aggregation:* Deforestation estimates aggregated at national level. Although it's theoretically possible to extract pixel-level emissions at 10-km resolution	*Data availability:* ✓<br>*Code for replicability:* ✓<br>*Updated post-publication:* ✗

		Comprehensiveness of estimates: Not quantified at commodity level	
Crippa et al.[110]	*Input*: FAOSTAT statistics<br>*Deforestation attribution model*: Not estimated. However, all land use and land-use changes from FAOSTAT are considered.<br>*Carbon emission accounting*: Statistical (includes all greenhouse gas emissions from the food supply chain)	*Spatial and temporal coverage:* Global (1990-2018)<br>*Spatial aggregation:* Carbon emission estimates aggregated at national level<br>*Comprehensiveness of estimates:* Not quantified at commodity level	*Data availability:* ✓<br>*Code for replicability:* ✗<br>*Updated post-publication:* ✓ (now covers 1990-2018)
Feng et al.[111] (uses Curtis et al. [70])	*Input*: Spatial tree cover loss and dominant drivers of forest loss<br>*Deforestation attribution model*: Spatial<br>*Carbon emission accounting*: Spatial (includes emission due to loss of AGB, BGB, SOC)	*Spatial and temporal coverage:* Tropical countries (2001-2019)<br>*Spatial aggregation:* Carbon emission estimates aggregated at national level. Although it's theoretically possible to extract pixel-level emissions at 10-km resolution<br>*Comprehensiveness of estimates:* Categorised into agriculture, forestry and other drivers	*Data availability:* ✓<br>*Code for replicability:* ✓<br>*Updated post-publication:* ✗
Curtis et al.[70] dominant driver (on which Global Forest Watch[100] estimates are based)	*Input*: Spatial tree cover loss and field training samples<br>*Deforestation attribution model*: Spatial<br>*Carbon emission accounting*: Not estimates	*Spatial and temporal coverage:* Global (Aggregated for whole time series, 2001-2022)<br>*Spatial aggregation:* Dominant deforestation driver estimates at 10-km resolution<br>*Comprehensiveness of estimates:* Dominant drivers of deforestation are broadly classified as Commodity-driven deforestation, Shifting agriculture, Forestry, Wildfire and Urbanisation.	*Data availability:* ✓<br>*Code for replicability:* ✓ (only initial code is available)<br>*Updated post-publication:* ✓ (now covers 2001-2022)

Supplementary Table 5 | Absolute values of deforestation and carbon emission estimates used for sensitivity analysis. The IDs will facilitate the association of results from various sensitivity analyses archived on Zenodo (see data availability).

<table>
<tr><th rowspan="2">ID</th><th rowspan="2">Broad category</th><th rowspan="2" colspan="2">Sensitivity control</th><th colspan="2">Sensitivity analysis (2001-2022)</th><th colspan="2">Reference analysis (2001-2022)</th><th rowspan="2">Remarks</th></tr>
<tr><th>Deforestation (Total; ha)</th><th>Carbon Emissions incl. peatland drainage (Total; MtCO$_2$)</th><th>Deforestation (Total; ha)</th><th>Carbon Emissions incl. peatland drainage (Total; MtCO$_2$)</th></tr>
<tr><td colspan="9">Forests are composed of trees established through natural regeneration. Conversion of these natural forests to other land uses is referred to as deforestation. Forest plantations, i.e., forests that are intensively managed for wood, fibre and energy, are excluded from this definition of forest.
This study: We define forest using tree cover threshold (≥25%; expressing canopy density for all vegetation taller than 5m in height within a pixel), with complete removal of tree cover canopy in a pixel representing tree cover loss. Using the spatio-temporal extent of forest plantation data, we exclude tree cover loss over forest plantations established prior to the year 2000 (i.e., rotational clearing; Supplementary Fig. 6), thus reflecting deforestation (i.e., loss of natural forests).
Sensitivity analysis: We modify tree cover thresholds, forest cover and deforestation data.</td></tr>
<tr><td>S1</td><td rowspan="4">Forest and deforestation</td><td colspan="2">Tree cover ≥ 10%</td><td>129,821,626</td><td>44,742</td><td rowspan="2">121,794,096</td><td rowspan="2">44,118</td><td rowspan="2">Lower tree cover threshold allows for inclusion of more forest loss pixels and vice versa</td></tr>
<tr><td>S2</td><td colspan="2">Tree cover ≥ 75%</td><td>85,276,875</td><td>39,280</td></tr>
<tr><td>S3</td><td colspan="2">JRC Global Forest Cover 2020
(compared only to estimates from 2020-2022)</td><td>12,088,808</td><td>3,362</td><td>13,605,957</td><td>5,577</td><td>Lower estimates are likely due to differing methodologies in delineating forests between JRC and Global Forest Change. Since JRC forest cover already excludes agricultural plantations (e.g., cocoa and oil palm plantations) from its forest coverage, this could be the possible reason for lower estimates.</td></tr>
<tr><td>S4</td><td colspan="2">JRC TMF Deforestation
(compared only for countries where TMF has spatial coverage)</td><td>72,163,858</td><td>34,757</td><td>100,460,663</td><td>42,825</td><td>JRC TMF deforestation accounts for disturbances over multiple years (excluding regions of regrowth to be classified as deforestation)[34], and excludes loss over dry forests (unlike GFC), thus being more conservative.</td></tr>
<tr><td rowspan="2">S5</td><td rowspan="2">Forest plantation</td><td rowspan="2">All plantations from SDPT established before the year 2000</td><td>(All commodity estimates)</td><td>121,756,874</td><td>44,116</td><td>121,794,096</td><td>44,118</td><td rowspan="2">Excluding all known forest plantation reduces deforestation attributed to forestry activities</td></tr>
<tr><td>(Forest plantation estimates only)</td><td>16,961,350</td><td>-247</td><td>16,989,601</td><td>-248</td></tr>
</table>

<table>
<tr><td colspan="9">The lag between the clearing of forest and the establishment of a productive agricultural or forestry land can vary widely depending on several factors, including the method of clearing, the intended use of the land, environmental conditions, and local agricultural practices.
This study: With spatio-temporal data, we attribute forest loss to land-use with a higher rotation period within a 4-year moving window (i.e., maximum lag of 3-years from the year of forest loss). The attribution is in the order of forest plantations, followed by woody perennial crops, pastures, herbaceous perennial and temporary crops. In statistical attribution, we use a lag period of 3 years.
Sensitivity analysis: We modify spatial and statistical lag, and the combined effect of both.</td></tr>
<tr><td>S6</td><td rowspan="6">Lag period</td><td colspan="2">Spatial lag period = 1 year
(compared only for MapBiomas countries)</td><td>60,479,552</td><td>25,915</td><td rowspan="2">67,161,919</td><td rowspan="2">28,469</td><td rowspan="6">Longer lag period captures more delayed land-use changes and vice versa</td></tr>
<tr><td>S7</td><td colspan="2">Spatial lag period = 5 year
(compared only for MapBiomas countries)</td><td>70,013,040</td><td>29,557</td></tr>
<tr><td>S8</td><td colspan="2">Statistical lag period = 1 year</td><td>120,912,241</td><td>43,749</td><td rowspan="2">121,794,096</td><td rowspan="2">44,118</td></tr>
<tr><td>S9</td><td colspan="2">Statistical lag period = 5 year</td><td>121,909,645</td><td>44,354</td></tr>
<tr><td>S10</td><td colspan="2">Both spatial and statistical lag = 1 year
(compared only for MapBiomas countries)</td><td>60,454,479</td><td>25,954</td><td rowspan="2">67,161,919</td><td rowspan="2">28,469</td></tr>
<tr><td>S11</td><td colspan="2">Both spatial and statistical lag = 5 year
(compared only for MapBiomas countries)</td><td>70,017,034</td><td>29,538</td></tr>
<tr><td colspan="9">This study: We overlay several spatial datasets providing extent of specific commodities, land use and dominant drivers to attribute forest loss.
Sensitivity analysis: We analyse deforestation attribution using only Dominant Driver of tree cover loss and only tree cover loss dataset.</td></tr>
<tr><td rowspan="4">S12</td><td rowspan="8">Inclusion of spatial datasets</td><td rowspan="4">Partial statistical attribution (Global Forest Change + Dominant driver + agricultural statistics)</td><td>Global</td><td>171,153,029</td><td>61,534</td><td>121,794,096</td><td>44,118</td><td rowspan="8">Poor quality data that overlooks spatio-temporal heterogeneity. Furthermore, deforestation from non-agriculture and forestry sectors (e.g., mining) might contribute to inflating these estimates, if not removed from attribution.</td></tr>
<tr><td>Oil palm-Indonesia</td><td>9,326,754</td><td>5,900</td><td>7,790,477</td><td>4,250</td></tr>
<tr><td>Cocoa-Côte d'Ivoire</td><td>634,953</td><td>137</td><td>896,994</td><td>238</td></tr>
<tr><td>Soya beans-Brazil</td><td>11,589,175</td><td>4,835</td><td>3,461,413</td><td>1,021</td></tr>
<tr><td rowspan="4">S13</td><td rowspan="4">Full statistical attribution (Global Forest Change + agricultural statistics)</td><td>Global</td><td>226,530,991</td><td>76,377</td><td>121,794,096</td><td>44,118</td></tr>
<tr><td>Oil palm-Indonesia</td><td>9,369,761</td><td>5,788</td><td>7,790,477</td><td>4,250</td></tr>
<tr><td>Cocoa-Côte d'Ivoire</td><td>637,999</td><td>138</td><td>896,994</td><td>238</td></tr>
<tr><td>Soya beans-Brazil</td><td>8,716,920</td><td>3,533</td><td>3,461,413</td><td>1,021</td></tr>
<tr><td colspan="9">This study: We use sub-national agricultural statistics to improve granularity of forest loss attribution in Brazil.
Sensitivity analysis: We directly assess deforestation in Brazil using FAOSTAT national agricultural statistics.</td></tr>
<tr><td>S14</td><td>Agriculture statistics</td><td colspan="2">National agricultural statistics
(analysed only for Brazil)</td><td>38,375,103</td><td>17,013</td><td>38,329,216</td><td>16,997</td><td>Different datasets</td></tr>
<tr><td colspan="9">Net land-use change shows the difference in total area between different time steps, while gross land-use change accounts for area gains and losses. In absence of spatio-temporal remote sensing dataset, it is difficult to discern gross losses over agricultural land systems.
This study: We use crop and grass loss data and an assumption that cropland expands over pastures as a proxy to statistically assess gross land-use expansion for agricultural land systems.</td></tr>
</table>

Sensitivity analysis: We analyse deforestation attribution assuming cropland directly led to deforestation (and do not expand over pastures first), and using net expansion estimates derived from agricultural statistics, not accounting for gross land-use change. Furthermore, we restrict (using only the right part for Supplementary equations (4)-(6) and don't restrict (left part for Supplementary equations (4)-(6) all land-use attributions.								
S15	**Land-use expansion**	Croplands do not expand over pastures, directly forests		122,067,977	44,249	121,794,096	44,118	More crop-commodity driven deforestation
		Net expansion for agricultural land systems		112,500,734	40,060			Net land-use change doesn't account for losses in pasture and crops (such as those resulting from crop failure), which in turn reduces the contribution of these commodities to deforestation estimates.
S16		All statistical land-use attribution restricted by FAOSTAT		120,295,888	43,389			Influences land-use expansion driven deforestation for certain and uncertain mosaics (see Supplementary equations (4)-(6))
S17		Statistical land-use attribution not restricted by FAOSTAT		147,103,233	56,508			
**Multi-cropping**: When two or more crops are grown on the same plot of land under different growing season.								
S18	**Multiple cropping**	Not accounting for harvested area from multiple cropping *(analysed only for Brazil)*	Maize	724,624	207	535,248	153	Not accounting for multi-cropping increases deforestation estimates for commodities with higher harvested areas (potentially due to proportional commodity attribution in Supplementary equation (9)-(12)), and vice versa.
			Beans	239,455	67	200,274	52	
			Potatoes	8,534	3.17	5,781	2.27	
			Groundnuts	7,730	2.48	8,323	2.73	
**Amortisation period** conceptually spreads the consequences of deforestation across multiple years to account for the enduring productivity of the land.<br>*This study:* We use a 5-year amortisation period.								
S19	**Amortisation period** *(compared with amortised estimates of year 2020)*	10 years *(i.e., average of 2011-2020)*		5,611,693	2,089	5,644,532	2,113	There is no universal global pattern, but selecting an appropriate amortization period can help reflect recent or historical trends for specific countries or commodities, such as changes in commodity demand, production trends, domestic consumption, and trade dynamics.
S20		15 years *(i.e., average of 2006-2020)*		5,739,464	2,160			
S21		20 years *(i.e., average of 2001-2020)*		5,625,950	2,134			
**ID**	**Broad category**	**Sensitivity control**		**Sensitivity analysis (2001-2022)**		**Reference analysis (2001-2022)**		**Remarks**
					**Peatland drainage emissions (Mt CO_2)**		**Peatland drainage emissions (Mt CO_2)**	
**Amortised peatland drainage emissions:**<br>*In this study*, we accumulate peatland drainage emissions till the year 2022 (end of the study period), starting from the year of deforestation.								

S22	**Accumulation period of peatland drainage emissions**	Global	No accumulation	-	248	-	2,885	While recognising the uncertainty surrounding the lifetime of peatland drainage emissions, assuming a shorter accumulation period for peatland drainage emissions can substantially underestimate their contribution to total carbon emissions associated with deforestation.
			5 years from the year of deforestation, or till 2022		1,186			
			10 years from the year of deforestation, or till 2022		2,132			
			15 years from the year of deforestation, or till 2022		2,681			
			20 years from the year of deforestation, or till 2022		2,869			
**Mature carbon stock of the replacing commodity:** Considering that the replacing commodity can only store a maximum of 75% of their mature carbon stock (Supplementary Table 9) in the net emissions calculation (see Eq. 1).								
S23	**Net carbon emissions** *(excluding peatland drainage emissions)*	Only considering 75% of the mature plant carbon stocks for the replacing commodity	All commodities-Global	-	44,598		41,233	Considering a lower maturity carbon stock for the replacing commodity in the net emissions calculation results in higher net carbon emissions.
			Tree crops-Global		6,067		5,209	
			Oil palm-Global		3,598		3,082	

Supplementary Table 6 | Scoring individual datasets for attribution and quality assessment. The criteria for the scoring methodology are detailed in Supplementary Table 11. Commodities are attributed in descending order of their scores, starting with the highest-scored commodity and proceeding to the lowest.

Dataset	Space	Time	Explicitness	Score	Special remarks
**Oil palm fruit** (Indonesia)	1.00	1.00	1.00	**1.00**	Reduce the score by 0.05 for every year after 2019
**Maize** (China)	0.90	1.00	1.00	**0.97**	
**Soya beans** (South America)	0.80	1.00	1.00	**0.93**	
**Sugarcane** (Brazil)	0.90	0.70	1.00	**0.87**	
**Oil palm fruit** (Malaysia)	0.65	0.90	1.00	**0.85**	Reduce the score by 0.05 for every year after 2018
**Cocoa** (Côte d'Ivoire and Ghana)	0.95	0.60	1.00	**0.85**	
**MapBiomas collection** (Commodities)	0.80	1.00	0.70	**0.83**	Includes only explicitly defined commodities
**Rice** (Asia)	0.90	0.60	1.00	**0.83**	
**Rapeseed** (North America, Canada, Europe and Chile)	0.85	0.60	1.00	**0.82**	
**Oil palm fruit** (Pan-tropical)	0.75	0.40	1.00	**0.72**	
**Coconut** (Pan-tropical)	0.70	0.40	1.00	**0.70**	
**Global plantation dataset**	0.65	0.80	0.65	**0.70**	
**MapBiomas collection** (Land use)	0.80	1.00	0.30	**0.70**	Includes all land-use classifications excluding commodities
**Croplands**	0.65	0.80	0.50	**0.65**	
**Forest loss due to fire**	0.65	1.00	0.10	**0.58**	Dataset not used for attribution, but for screening forest loss due to fire
**Global forest change** (Forest loss)	0.65	0.85	0.10	**0.53**	
**Dominant forest loss drivers**	0.10	0.70	0.40	**0.40**	
**Subnational stats**	1.00	1.00	1.00	**-**	We do not penalise this dataset when flagging (equation (2))
**FAOSTAT national stats**	0.50	1.00	1.00	**-**	Besides penalising the dataset based on flags (equation (2); Supplementary Table 12), we further reduce the FAOSTAT dataset score by '-0.50/3' for both land use and production statistics individually.

Supplementary Table 7 | Pre-processing and attribution assumptions for the spatial datasets.

Datasets	Pre-processing and attribution assumptions
Global forest change	- Forest loss is only considered for pixels with tree cover ≥ 25%
Global plantation dataset	- Only considered as forest plantation-driven deforestation if the start year of the dataset > 2000. 'Start year' defines the year when the first plantation was established based on the temporal extent of remote sensing datasets<br>- Forest loss pixels classified with start year ≤ 2000 are considered under rotational clearing and excluded from deforestation attribution<br>- *Attribution*: For this dataset, deforestation attribution is not temporally restricted
MapBiomas Collection	- Forest loss is attributed to MapBiomas when a commodity-driven land use occurs within a four-year window from the year of forest loss<br>- In case of multiple land use changes occurring within this four-year window, forest plantations will be prioritised over perennial crops, and perennial crops prioritised over pastures, followed by temporary crops<br>- If MapBiomas(t) land use is the same as MapBiomas(2000), we consider forest loss as 'historical/rotational clearing'<br>- *Attribution*: For this dataset, deforestation attribution is temporally restricted to 2021 only for Bolivia; not restricted for other MapBiomas countries
Croplands	- Forest loss recorded from 2001 to 2003 is attributed to cropland only if cropland extent is defined for the period of 2000-2003<br>- Forest loss recorded from 2001 to 2007 is attributed to cropland defined for the period of 2004-2007. The delay between forest loss and cropland extent is given to accommodate for forest loss and establishment of cropland<br>- Forest loss recorded from 2005 to 2011 is attributed to cropland defined for the period of 2008-2011<br>- Forest loss recorded from 2008 to 2015 is attributed to cropland defined for the period of 2012-2015<br>- Forest loss recorded from 2012 to 2019 is attributed to cropland defined for the period of 2016-2019<br>- *Attribution*: For this dataset, deforestation attribution (following above) is temporally restricted to 2019
Sugarcane	- *Attribution*: For this dataset, deforestation attribution is temporally restricted to 2019
Soya beans	- Forest loss is attributed to Soya beans when a Soya bean land use occurs within a four-year window from the year of forest loss<br>- *Attribution*: For this dataset, deforestation attribution is temporally restricted to 2022
Rice	- Resolution of the dataset is downscaled to 30 m (same resolution as Global forest change), determined by the majority of pixels within the designated reducer window<br>- *Attribution*: For this dataset, deforestation attribution takes place to 2019
Rapeseed	- Resolution of the dataset is downscaled to 30 m (same resolution as Global forest change), determined by the majority of pixels within the designated reducer window<br>- *Attribution*: For this dataset, deforestation attribution is temporally restricted to 2019
Maize (corn)	- Forest loss is attributed to Maize when a Maize land use occurs within a four-year window from the year of forest loss<br>- *Attribution*: For this dataset, deforestation attribution is temporally restricted to 2020
Cocoa	- Resolution of the dataset is downscaled to 30 m (same resolution as Global forest change), determined by the majority of pixels within the designated reducer window<br>- Pixels of forest loss classified as Cocoa and overlapping with plantation mask are considered under 'rotational clearing'<br>- *Attribution*: For this dataset, deforestation attribution is temporally restricted to 2021
Coconut	- Resolution of the dataset is downscaled to 30 m (same resolution as Global forest change), determined by the majority of pixels within the designated reducer window<br>- Pixels of forest loss classified as Coconut and overlapping with plantation mask are considered under 'rotational clearing'<br>- *Attribution*: For this dataset, deforestation attribution is temporally restricted to 2020

Oil palm fruit (Indonesia)	- Forest loss occurring in the regions (i.e., delineated within a boundary) of Oil palm plantations for the year 2000 are classified as 'rotational clearing', and these pixels are excluded from commodity-driven deforestation<br>- *Attribution*: This dataset is not temporally restricted, thus assuming that if a forest loss occurs in a pixel post-2019 (data's temporal extent), we consider it as forest loss due to Oil palm for that year
Oil palm fruit (Malaysia)	- Forest loss is attributed to Oil palm when an Oil palm land use occurs within a four-year window from the year of forest loss<br>- Pixels of forest loss classified as Oil palm and overlapping with plantation mask are considered under 'rotational clearing'<br>- *Attribution*: This dataset is not temporally restricted, thus assuming that if a forest loss occurs in a pixel post-2018 (data's temporal extent), we consider it as forest loss due to Oil palm for that year
Oil palm fruit (Global)	- Resolution of the dataset is downscaled to 30 m (same resolution as Global forest change), determined by the majority of pixels within the designated reducer window<br>- Pixels of forest loss classified as Oil palm and overlapping with plantation mask are considered under 'rotational clearing'<br>- *Attribution*: For this dataset, deforestation attribution is temporally restricted to 2019
Forest loss due to fire	- Forest loss pixels classified under '1. Forest loss due to other (non-fire) drivers' are open for attribution by other datasets<br>- Forest loss pixels classified under '2. Low certainty of forest loss due to fire' are open for attribution by other datasets<br>- Forest loss pixels classified under '3. Medium' and '4. High' certainty are excluded from commodity-driven deforestation<br>- Forest loss pixels classified under '5. Forest loss due to fire in Africa' are excluded from commodity-driven deforestation
Forest management	- Forest loss is considered 'rotational clearing' if the pixel falls under '20. Naturally regenerating forest with signs of management, e.g., logging, clear cuts etc', '31: Planted forests (rotation >15 years)', '32: Plantation forests (rotation ≤15 years)', '40: Oil palm plantations' and '53: Agroforestry'<br>- The above only applies to the spatial extent of countries covered in Supplementary Table 2 for 'Forest management'
Dominant drivers of forest loss	- Forest loss pixels classified under 'Commodity-driven deforestation' and 'Shifting agriculture' are considered under agricultural-driven deforestation<br>- Forest loss pixels classified under 'Forestry' are considered under forestry-induced deforestation<br>- Forest loss pixels classified under 'Wildfire' and 'Urbanisation' are excluded from commodity-driven deforestation<br>- Pixels of forest loss classified by this dataset and overlapping with plantation mask are considered under 'rotational clearing'

Supplementary Table 8 | Loss of soil organic carbon (SOC) across different land use and biomes. The values represent the % loss of actual SOC. Note that for depths 30-100 cm, the data is scarce. Thus, we use the 0-100 cm data to estimate SOC loss for 30-100 cm depth. We do this by assuming that SOC $\text{loss}_{0\text{-}100\ \text{cm}}$ = SOC $\text{loss}_{0\text{-}30\ \text{cm}}$ + SOC $\text{loss}_{30\text{-}100\ \text{cm}}$.

		Land use replacing forest (values in %)			
**Depth**	**Ecoregion group**	**Cropland**	**Pasture**	**Forest plantation**	**References**
0-30 cm	Global	26.6	18	13	112,113
0-30 cm	Tropical	29	4	22	114–116
0-30 cm	Temperate	31.4	4.15	15	113,117,118
0-30 cm	Boreal	21	18†	13†	119
30-100 cm	Global	13.8#	9.7#	23#	112,120
30-100 cm	Tropical	15	2	7	116
30-100 cm	Temperate	25	6.925*	19*	117
30-100 cm	Boreal	17.4*	13.85*	18*	

†Imputed using global average estimates

#Values available for depths of 0-100 cm

*Calculated using the average of global and respective ecoregions 0-30m estimates; consider these values for 0-100 cm

Supplementary Table 9 | Plant carbon stocks of replacing commodities and commodity groups across different biomes.

<table>
<tr><th></th><th colspan="3">(Values in MgC ha^{-1})</th><th></th></tr>
<tr><th>Crop or Commodity group</th><th>Tropical</th><th>Temperate</th><th>Boreal</th><th>References</th></tr>
<tr><td>Cereals</td><td>4.44</td><td colspan="2">3.15</td><td>121,122</td></tr>
<tr><td>Maize (corn)</td><td colspan="3">6.3</td><td>121</td></tr>
<tr><td>Rice</td><td colspan="3">4.5</td><td>121</td></tr>
<tr><td>Wheat</td><td colspan="3">2.3</td><td>121</td></tr>
<tr><td>Barley</td><td colspan="3">5.5</td><td>123</td></tr>
<tr><td>Sorghum</td><td colspan="3">4.12</td><td>121</td></tr>
<tr><td>Millet</td><td colspan="3">3.13</td><td>124</td></tr>
<tr><td>Edible roots and tubers with high starch or inulin content</td><td colspan="3">3</td><td>122</td></tr>
<tr><td>Cassava</td><td colspan="3">4.5</td><td>125</td></tr>
<tr><td>Potatoes</td><td colspan="3">0.5</td><td>126</td></tr>
<tr><td>Fibre crops</td><td colspan="3">3.71</td><td>121</td></tr>
<tr><td>Natural rubber in primary forms</td><td colspan="3">79.05</td><td>127</td></tr>
<tr><td>Jute, raw or retted</td><td colspan="3">3.9</td><td>121</td></tr>
<tr><td>Seed cotton, unginned</td><td colspan="3">4.3</td><td>121</td></tr>
<tr><td>Forest plantation</td><td>120.23</td><td>130.99</td><td>96.07</td><td>91,128</td></tr>
<tr><td>Fruit and nuts</td><td>31.96</td><td colspan="2">39.53</td><td>129,130</td></tr>
<tr><td>Apples</td><td colspan="3">26.48</td><td>131</td></tr>
<tr><td>Bananas</td><td colspan="3">6.2</td><td>132</td></tr>
<tr><td>Cashew nuts, in shell</td><td colspan="3">37.6</td><td>133</td></tr>
<tr><td>Grapes</td><td colspan="3">12.3</td><td>134</td></tr>
<tr><td>Mangoes</td><td colspan="3">84.75</td><td>135</td></tr>
<tr><td>Oranges</td><td colspan="3">7.69</td><td>136</td></tr>
<tr><td>Other citrus fruit, n.e.c.</td><td>20.65</td><td colspan="2">23.73</td><td>132</td></tr>
<tr><td>Plantains and cooking bananas</td><td colspan="3">6.2</td><td>132</td></tr>
</table>

**Oilseeds and oleaginous fruits**	31.96	39.53	129,130
Oil palm fruit	52.28		127
Soya beans	3		121
Sunflower seed	1.1		121
Groundnuts, excluding shelled	1.1		121
Olives	5.3		137
Coconuts, in shell	57.38	65.93	132
**Pasture**	6.8		121
**Pulses (dried leguminous vegetables)**	1.56		121
Beans, dry	2.39		124
Chick peas, dry	1.28		124
Cow peas, dry	1.82		124
Pigeon peas, dry	3		124
Lentils, dry	1.25		124
Peas, dry	0.9		121
**Stimulant, spice and aromatic crops**	31.96	39.53	129,130
Coffee, green	77.12		138
Cocoa beans	34.55		139
Tea leaves	21.06		140
**Sugar crops**	10.17		*Average of commodities in the group*
Sugar beet	8.32		126
Sugar cane	12.02		141
**Vegetables**	0.43		142
Cabbages	1.65		121
Lettuce and chicory	1.15		143
Tomatoes	3.48		143
Cauliflowers and broccoli	4.05		143

Supplementary Table 10 | Emission factor used to estimate carbon emissions from deforestation on peatlands. Emission factors from ref.[79] are based on IPCC Wetland Supplement[51].

	**(values in $MgCO_2$ ha^{-1} yr^{-1})**			
**Land use replacing forest**	**Tropical**	**Temperate**	**Boreal**	**References**
Cropland	45	28.6	27.9	79
Pasture	37.4	17.95	20.2	79
Forest plantation	40.34	2.5	6.42	51,78
Oil palm fruit	54.41			77

Supplementary Table 11 | Criteria's for scoring different aspects of spatial datasets.

Aspect	Criteria	Penalisation
**Space** (representing both resolution and area of focus)	Perfect score is given when the pixel size is ≤ 10m and is explicitly mapped for a country	0
	Resolution of 20 m	-0.05
	Resolution of 30 m	-0.1
	Resolution of 100 m	-0.3
	Resolution of 1 km	-0.5
	Resolution of 10 km	-0.75
	Mapped for two countries	-0.05
	Mapped for more than two countries or a continent	-0.1
	Multiple continents	-0.15
	Mapped globally	-0.25
**Time** (representing temporal resolution and standalone ability of the data to differentiate pre- and post-2000's deforestation)	Perfect score is given when the dataset is available from 2001-2022 for herbaceous crops, and at least the year 2000- or prior-onwards for woody vegetation crops (i.e., tree crops) and forest plantations (allowing for differentiation between post-2000's deforestation from the rotational clearing of managed plantations)	0
	For tree crops and forest plantations, deforestation is not differentiable from rotational clearing (need to be complimented with plantation mask to extract this information)	Using Du et al: -0.1<br>Using Lesiv et al: -0.2
	After the latest detection year (in cases allowed)	-0.05 each year
	Temporal aggregation based on a single year of remote sensing dataset	-0.3
	Temporal aggregation based on 2-3 years of remote sensing dataset	-0.2
	Temporal aggregation based on 4-6 years of remote sensing dataset	-0.1
	Temporal aggregation based on >6 years of remote sensing dataset	0
	Temporally-explicit estimates every 2-3 years between 2001-2022	-0.1
	Temporally-explicit estimates every 4-6 years between 2001-2022	-0.2
	Temporally-explicit estimates >6 years between 2001-2022	-0.3
	Starting year of detection is 1-5 years away from 2001 (i.e., the first year of analysed deforestation)	-0.05
	Starting year of detection is 6-10 years away from 2001	-0.1
	Starting year of detection is 11-15 years away from 2001	-0.15
	Starting year of detection is >15 years away from 2001	-0.2
**Explicitness** (representation of the deforestation driver and consideration given to training algorithm of the data)	Perfect score is given to datasets that maps a single commodity, where model training is performed using field samples	0
	When training is primarily based on remote sensing trends, without using field samples (including visual interpretations)	-0.5
	When multiple commodities or land uses are predicted by the same model using the same field samples	-0.1
	Dataset maps two or more than two commodities (differentiable)	-0.2
	Dataset maps a single land use	-0.3
	Dataset maps two or more than two different land uses (differentiable)	-0.4
	Dataset maps two or more than two different land uses (indifferentiable, i.e., mosaics)	-0.6
	Information about forest loss drivers is unavailable	-0.9

Supplementary Table 12 | The FAO flags, their description and associated penalisation. A detailed description of FAO flags is documented in ref.[144]. Since our statistical attribution relies on the expansion of land-use and commodities, we obtain flags for two years (*t+lag* and *t*; see Supplementary equations (1) and (9)). In the quality assessment, we use the flag with the lower penalization between the two.

Flag	Description	Penalisation
A	**Official figure**: Value provided as official when the source agency assigns sufficient confidence that it is not expected to be dramatically revised	0
B	**Time series break**: Observations are characterised as such when different content exists or a different methodology has been applied to this observation as compared with the preceding one	-0.10
E	**Estimated value**: Observation obtained through an estimation methodology or based on the use of a limited amount of data	-0.20
I	**Imputed value**: Observation imputed by a receiving agency to replace or fill gaps in reported data series	-0.30
P	**Provisional value**: An observation is characterised as "provisional" when the source agency – while it bases its calculations on its standard production methodology – considers that the data, almost certainly, are expected to be revised	-0.40
T	**Unofficial figure**: Observations are "temporary" or "tentative", indicating that the figure should be used with caution and may be subject to revision or replacement with official statistics once they become available.	-0.40
X	**Figure from international organisations**: Observation from an international or a supranational organisation that does not use any flagging system in data sharing	-0.50
M	**Missing value**: Used to denote empty cells resulting from the impossibility to collect a statistical value	-0.70
Z	**Authors' gap filling**: Gap filled by authors of this study (*not part of FAOSTAT flags*)	-0.70

Supplementary references

1. Ma, Y. *et al.* Remote sensing big data computing: Challenges and opportunities. *Future Generation Computer Systems* **51**, 47–60 (2015).
2. Wang, Y. *et al.* A review of regional and Global scale Land Use/Land Cover (LULC) mapping products generated from satellite remote sensing. *ISPRS Journal of Photogrammetry and Remote Sensing* **206**, 311–334 (2023).
3. Hansen, M. C. *et al.* High-Resolution Global Maps of 21st-Century Forest Cover Change. *Science* **342**, 850–853 (2013).
4. Tropical Moist Forests product - Data Access. https://forobs.jrc.ec.europa.eu/TMF/data.php.
5. Fischer, R. *et al.* Accelerated forest fragmentation leads to critical increase in tropical forest edge area. *Science Advances* **7**, eabg7012 (2021).
6. Schielein, J. & Börner, J. Recent transformations of land-use and land-cover dynamics across different deforestation frontiers in the Brazilian Amazon. *Land Use Policy* **76**, 81–94 (2018).
7. Fassnacht, F. E., White, J. C., Wulder, M. A. & Næsset, E. Remote sensing in forestry: current challenges, considerations and directions. *Forestry: An International Journal of Forest Research* **97**, 11–37 (2024).
8. Lesiv, M. *et al.* Global forest management data for 2015 at a 100 m resolution. *Sci Data* **9**, 199 (2022).
9. Parente, L. *et al.* Annual 30-m maps of global grassland class and extent (2000–2022) based on spatiotemporal Machine Learning. *Sci Data* **11**, 1303 (2024).
10. Carreiras, J. M. B., Jones, J., Lucas, R. M. & Gabriel, C. Land Use and Land Cover Change Dynamics across the Brazilian Amazon: Insights from Extensive Time-Series Analysis of Remote Sensing Data. *PLOS ONE* **9**, e104144 (2014).
11. Jiang, C., Guan, K., Huang, Y. & Jong, M. A vehicle imaging approach to acquire ground truth data for upscaling to satellite data: A case study for estimating harvesting dates. *Remote Sensing of Environment* **300**, 113894 (2024).
12. FAOSTAT. https://www.fao.org/faostat/en/#data.
13. Instituto Brasileiro de Geografia e Estatística (IBGE). IBGE Produção Agrícola Municipal. https://sidra.ibge.gov.br/pesquisa/pam/tabelas (2022).
14. Agricultural production statistics (2000–2022). *Statistics* https://www.fao.org/statistics/highlights-archive/highlights-detail/agricultural-production-statistics-(2000-2022)/en.
15. Artaxo, P. *et al.* Chapter 23: Impacts of deforestation and climate change on biodiversity, ecological processes, and environmental adaptation. in *Amazon Assessment Report 2021* (eds Nobre, C. et al.) (UN Sustainable Development Solutions Network (SDSN), 2021). doi:10.55161/VKMN1905.
16. Alves de Oliveira, B. F., Bottino, M. J., Nobre, P. & Nobre, C. A. Deforestation and climate change are projected to increase heat stress risk in the Brazilian Amazon. *Commun Earth Environ* **2**, 1–8 (2021).
17. Godde, C. M., Mason-D'Croz, D., Mayberry, D. E., Thornton, P. K. & Herrero, M. Impacts of climate change on the livestock food supply chain; a review of the evidence. *Global Food Security* **28**, 100488 (2021).
18. Davis, K. F., Downs, S. & Gephart, J. A. Towards food supply chain resilience to environmental shocks. *Nat Food* **2**, 54–65 (2021).
19. Grabs, J., Cammelli, F., Levy, S. A. & Garrett, R. D. Designing effective and equitable zero-deforestation supply chain policies. *Global Environmental Change* **70**, 102357 (2021).
20. Song, X.-P. *et al.* Massive soybean expansion in South America since 2000 and implications for conservation. *Nat Sustain* 1–9 (2021) doi:10.1038/s41893-021-00729-z.
21. Kalischek, N. *et al.* Cocoa plantations are associated with deforestation in Côte d'Ivoire and Ghana. *Nat Food* **4**, 384–393 (2023).
22. Descals, A., Gaveau, D. L. A., Wich, S., Szantoi, Z. & Meijaard, E. Global mapping of oil palm planting year from 1990 to 2021. *Earth System Science Data* **16**, 5111–5129 (2024).
23. Davis, K. F. *et al.* HarvestStat: a global effort towards open and standardized sub-national agricultural data. *Environ. Res. Lett.* **20**, 052001 (2025).
24. Tang, F. H. M. *et al.* CROPGRIDS: a global geo-referenced dataset of 173 crops. *Sci Data* **11**, 413 (2024).
25. You, L., Wood, S., Wood-Sichra, U. & Wu, W. Generating global crop distribution maps: From census to grid. *Agricultural Systems* **127**, 53–60 (2014).
26. Qin, X., Wu, B., Zeng, H., Zhang, M. & Tian, F. Global Gridded Crop Production Dataset at 10 km Resolution from 2010 to 2020. *Sci Data* **11**, 1377 (2024).
27. Xie, H. *et al.* GloRice, a global rice database (v1.0): I. Gridded paddy rice annual distribution from 1961 to 2021. *Sci Data* **12**, 182 (2025).
28. Cao, J. *et al.* Mapping global yields of four major crops at 5-minute resolution from 1982 to 2015 using multi-source data and machine learning. *Sci Data* **12**, 357 (2025).
29. WRI & WBCSD. Greenhouse Gas Protocol Land Sector and Removals Guidance.

30. Accountability Framework initiative - Accountability Framework. https://accountability-framework.org/the-accountability-framework/download-the-full-framework/.
31. Forests, Land and Agriculture - Science Based Targets. *Science Based Targets Initiative* https://sciencebasedtargets.org/sectors/forest-land-and-agriculture.
32. Deforestation Risk Assessment Methodology. *World Cocoa Foundation* https://worldcocoafoundation.org/programmes-and-initiatives/deforestation-risk-assessment-methodology.
33. Teo, H. C. *et al.* Uncertainties in deforestation emission baseline methodologies and implications for carbon markets. *Nat Commun* **14**, 8277 (2023).
34. Sims, M. *et al.* Differences Between Global Forest Watch's Tree Cover Loss Data and JRC's Tropical Moist Forest Data Explained. *Global Forest Watch Content* https://www.globalforestwatch.org/blog/data-and-tools/tree-cover-loss-and-tropical-moist-forest-data-compared (2024).
35. Rochmyaningsih, D. New European rules to curb deforestation have worrying flaws, scientists say. *Science* **385**, 485–485 (2024).
36. Olofsson, P. *et al.* Good practices for estimating area and assessing accuracy of land change. *Remote Sensing of Environment* **148**, 42–57 (2014).
37. Pendrill, F. *et al.* Disentangling the numbers behind agriculture-driven tropical deforestation. *Science* **377**, eabm9267 (2022).
38. Pendrill, F. *et al.* Agricultural and forestry trade drives large share of tropical deforestation emissions. *Global Environmental Change* **56**, 1–10 (2019).
39. Masolele, R. N. *et al.* Mapping the diversity of land uses following deforestation across Africa. *Sci Rep* **14**, 1681 (2024).
40. Wang, Y. *et al.* Upturn in secondary forest clearing buffers primary forest loss in the Brazilian Amazon. *Nat Sustain* **3**, 290–295 (2020).
41. Trase. SEI-PCS Bolivia soy v1.0 supply chain map: Data sources and methods. https://doi.org/10.48650/E6B0-9871 (2023) doi:10.48650/E6B0-9871.
42. Trase - Insights and analysis on commodity trade sustainability - Trase. *trase.earth* https://trase.earth/.
43. Goldman, E., Weisse, M., Harris, N. & Schneider, M. Estimating the Role of Seven Commodities in Agriculture-Linked Deforestation: Oil Palm, Soy, Cattle, Wood Fiber, Cocoa, Coffee, and Rubber. *WRIPUB* https://doi.org/10.46830/writn.na.00001 (2020) doi:10.46830/writn.na.00001.
44. Yu, Q. *et al.* A cultivated planet in 2010 – Part 2: The global gridded agricultural-production maps. *Earth System Science Data* **12**, 3545–3572 (2020).
45. EarthStat - GIS data for agriculture and the environment. *EarthStat* http://www.earthstat.org/.
46. Renier, C. *et al.* Direct and indirect deforestation for cocoa in the tropical moist forests of Ghana. *Environ. Res.: Food Syst.* **2**, 025006 (2025).
47. Waha, K. *et al.* Multiple cropping systems of the world and the potential for increasing cropping intensity. *Global Environmental Change* **64**, 102131 (2020).
48. Waha, K. *et al.* Land use modelling needs to better account for multiple cropping to inform pathways for sustainable agriculture. *Commun Earth Environ* **6**, 756 (2025).
49. Van Tricht, K. *et al.* WorldCereal: a dynamic open-source system for global-scale, seasonal, and reproducible crop and irrigation mapping. *Earth System Science Data* **15**, 5491–5515 (2023).
50. Persson, U. M., Henders, S. & Cederberg, C. A method for calculating a land-use change carbon footprint (LUC-CFP) for agricultural commodities - applications to Brazilian beef and soy, Indonesian palm oil. *Glob Change Biol* **20**, 3482–3491 (2014).
51. *2006 IPCC Guidelines for National Greenhouse Gas Inventories - Volume 4*. https://www.ipcc.ch/report/2006-ipcc-guidelines-for-national-greenhouse-gas-inventories/ (2006).
52. Maciel, V. G. *et al.* Towards a non-ambiguous view of the amortization period for quantifying direct land-use change in LCA. *Int J Life Cycle Assess* **27**, 1299–1315 (2022).
53. IPCC. *Revised 1996 IPCC Guidelines for National Greenhouse Gas Inventories (Volume 3)*. https://www.ipcc-nggip.iges.or.jp/public/gl/guidelin/ch5ref1.pdf (1996).
54. zu Ermgassen, E. K. H. J. *et al.* The origin, supply chain, and deforestation risk of Brazil's beef exports. *Proceedings of the National Academy of Sciences* **117**, 31770–31779 (2020).
55. FAO-FRA. Global Forest Resource Assessment 2020. https://fra-data.fao.org/assessments/fra/2020 (2023).
56. MapBiomas. *MapBiomas General "Handbook": Algorithm Theoretical Basis Document (ATBD)*. https://mapbiomas-br-site.s3.amazonaws.com/ATBD_Collection_7_v2.pdf (2022).
57. Gaveau, D. L. A. *et al.* Slowing deforestation in Indonesia follows declining oil palm expansion and lower oil prices. *PLOS ONE* **17**, e0266178 (2022).
58. Du, Z. *et al.* A global map of planting years of plantations. *Sci Data* **9**, 141 (2022).

59. Harris, N., Goldman, E. D. & Gibbes, S. *Spatial Database of Planted Trees (SDPT Version 1.0)*. https://www.wri.org/research/spatial-database-planted-trees-sdpt-version-10 (2019).
60. Maraseni, T. N., Son, H. L., Cockfield, G., Duy, H. V. & Nghia, T. D. Comparing the financial returns from acacia plantations with different plantation densities and rotation ages in Vietnam. *Forest Policy and Economics* **83**, 80–87 (2017).
61. Steinfeld, H. *Livestock's Long Shadow: Environmental Issues and Options*. (Food and Agriculture Organization of the United Nations, Rome, 2006).
62. Gibbs, H. K. *et al.* Brazil's Soy Moratorium. *Science* **347**, 377–378 (2015).
63. Gaveau, D. L. A. *et al.* Rapid conversions and avoided deforestation: Examining four decades of industrial plantation expansion in Borneo. *Sci. Rep.* **6**, (2016).
64. Li, W. *et al.* Gross and net land cover changes in the main plant functional types derived from the annual ESA CCI land cover maps (1992–2015). *Earth System Science Data* **10**, 219–234 (2018).
65. Harper, K. L. *et al.* A 29-year time series of annual 300m resolution plant-functional-type maps for climate models. *Earth System Science Data* **15**, 1465–1499 (2023).
66. Graesser, J., Aide, T. M., Grau, H. R. & Ramankutty, N. Cropland/pastureland dynamics and the slowdown of deforestation in Latin America. *Environ. Res. Lett.* **10**, 034017 (2015).
67. Pendrill, F., Persson, U. M., Godar, J. & Kastner, T. Deforestation displaced: trade in forest-risk commodities and the prospects for a global forest transition. *Environ. Res. Lett.* **14**, 055003 (2019).
68. Opio, C. *et al. Greenhouse Gas Emissions from Ruminant Supply Chains – a Global Life Cycle Assessment*. (Food and Agriculture Organization of the United Nations, Rome, 2013).
69. Pendrill, F. *et al.* Disentangling the numbers behind agriculture-driven tropical deforestation. *Science* **377**, eabm9267 (2022).
70. Curtis, P. G., Slay, C. M., Harris, N. L., Tyukavina, A. & Hansen, M. C. Classifying drivers of global forest loss. *Science* **361**, 1108–1111 (2018).
71. Ahlström, A., Canadell, J. G. & Metcalfe, D. B. Widespread Unquantified Conversion of Old Boreal Forests to Plantations. *Earth's Future* **10**, e2022EF003221 (2022).
72. Gilbert, M. *et al.* Global distribution data for cattle, buffaloes, horses, sheep, goats, pigs, chickens and ducks in 2010. *Sci Data* **5**, 180227 (2018).
73. Rahimi, J. *et al.* A shift from cattle to camel and goat farming can sustain milk production with lower inputs and emissions in north sub-Saharan Africa's drylands. *Nat Food* **3**, 523–531 (2022).
74. Fernández, P. D. *et al.* Understanding the distribution of cattle production systems in the South American Chaco. *Journal of Land Use Science* **15**, 52–68 (2020).
75. Yoshikawa, S. Agro-Pastoral Expansion and Land Use/Land Cover Change Dynamics in Mato Grosso, Brazil. *Earth* **4**, 823–844 (2023).
76. John Couwenberg. *Emission Factors for Managed Peat Soils - An Analysis of IPCC Default Values*. https://www.wetlands.org/publications/emission-factors-for-managed-peat-soils-an-analysis-of-ipcc-default-values/ (2009).
77. Cooper, H. V. *et al.* Greenhouse gas emissions resulting from conversion of peat swamp forest to oil palm plantation. *Nat Commun* **11**, 407 (2020).
78. John Couwenberg. Greenhouse gas emissions from managed peat soils: is the IPCC reporting guidance realistic? *Mires and Peat 8* **Art. 2**, (2011).
79. Günther, A. *et al.* Prompt rewetting of drained peatlands reduces climate warming despite methane emissions. *Nat Commun* **11**, 1644 (2020).
80. Descals, A. *et al.* High-resolution global map of smallholder and industrial closed-canopy oil palm plantations. *Earth System Science Data* **13**, 1211–1231 (2021).
81. Potapov, P. *et al.* Global maps of cropland extent and change show accelerated cropland expansion in the twenty-first century. *Nat Food* **3**, 19–28 (2022).
82. Zheng, Y., dos Santos Luciano, A. C., Dong, J. & Yuan, W. High-resolution map of sugarcane cultivation in Brazil using a phenology-based method. *Earth System Science Data* **14**, 2065–2080 (2022).
83. Han, J. *et al.* NESEA-Rice10: high-resolution annual paddy rice maps for Northeast and Southeast Asia from 2017 to 2019. *Earth System Science Data* **13**, 5969–5986 (2021).
84. Han, J. *et al.* The RapeseedMap10 database: annual maps of rapeseed at a spatial resolution of 10m based on multi-source data. *Earth System Science Data* **13**, 2857–2874 (2021).
85. Peng, Q. *et al.* A twenty-year dataset of high-resolution maize distribution in China. *Sci Data* **10**, 658 (2023).
86. Descals, A. *et al.* High-resolution global map of closed-canopy coconut palm. *Earth System Science Data* **15**, 3991–4010 (2023).

87. Xu, Y. *et al.* Annual oil palm plantation maps in Malaysia and Indonesia from 2001 to 2016. *Earth System Science Data* **12**, 847–867 (2020).
88. Tyukavina, A. *et al.* Global Trends of Forest Loss Due to Fire From 2001 to 2019. *Frontiers in Remote Sensing* **3**, (2022).
89. Agriculture, Forestry, Fishery and Animal Husbandry Census. *National Statistics, Republic of China (Taiwan)* https://eng.stat.gov.tw/cl.aspx?n=2405.
90. Copernicus Climate Change Service. Land cover classification gridded maps from 1992 to present derived from satellite observations. ECMWF https://doi.org/10.24381/CDS.006F2C9A (2019).
91. Mokany, K., Raison, R. J. & Prokushkin, A. S. Critical analysis of root : shoot ratios in terrestrial biomes. *Global Change Biology* **12**, 84–96 (2006).
92. Harris, N. L. *et al.* Global maps of twenty-first century forest carbon fluxes. *Nat. Clim. Chang.* **11**, 234–240 (2021).
93. Huang, Y. *et al.* A global map of root biomass across the world's forests. *Earth System Science Data* **13**, 4263–4274 (2021).
94. Poggio, L. *et al.* SoilGrids 2.0: producing soil information for the globe with quantified spatial uncertainty. *SOIL* **7**, 217–240 (2021).
95. Crezee, B. *et al.* Mapping peat thickness and carbon stocks of the central Congo Basin using field data. *Nat. Geosci.* **15**, 639–644 (2022).
96. Gumbricht, T. *et al.* An expert system model for mapping tropical wetlands and peatlands reveals South America as the largest contributor. *Global Change Biology* **23**, 3581–3599 (2017).
97. Hastie, A. *et al.* Risks to carbon storage from land-use change revealed by peat thickness maps of Peru. *Nat. Geosci.* **15**, 369–374 (2022).
98. Xu, J., Morris, P. J., Liu, J. & Holden, J. PEATMAP: Refining estimates of global peatland distribution based on a meta-analysis. *CATENA* **160**, 134–140 (2018).
99. Miettinen, J., Shi, C. & Liew, S. C. Land cover distribution in the peatlands of Peninsular Malaysia, Sumatra and Borneo in 2015 with changes since 1990. *Global Ecology and Conservation* **6**, 67–78 (2016).
100. Global Forest Watch (GFW). Global Peatlands. https://data.globalforestwatch.org/datasets/gfw::global-peatlands/about.
101. Dinerstein, E. *et al.* An Ecoregion-Based Approach to Protecting Half the Terrestrial Realm. *BioScience* **67**, 534–545 (2017).
102. Funk, C. *et al.* The climate hazards infrared precipitation with stations—a new environmental record for monitoring extremes. *Scientific Data* **2**, 150066 (2015).
103. Jarvis, A., Guevara, E., Reuter, H. I. & Nelson, A. D. Hole-filled SRTM for the globe : version 4 : data grid. (2008).
104. GADM. Database of Global Administrative Areas (Version v4.1). https://gadm.org/.
105. zu Ermgassen, E. K. H. J. *et al.* The origin, supply chain, and deforestation risk of Brazil's beef exports. *Proceedings of the National Academy of Sciences* **117**, 31770–31779 (2020).
106. Sy, V. D. *et al.* Land use patterns and related carbon losses following deforestation in South America. *Environ. Res. Lett.* **10**, 124004 (2015).
107. Zalles, V. *et al.* Near doubling of Brazil's intensive row crop area since 2000. *PNAS* **116**, 428–435 (2019).
108. Orbae - by Adastra. https://orbae.adastra.eco/.
109. Hoang, N. T. & Kanemoto, K. Mapping the deforestation footprint of nations reveals growing threat to tropical forests. *Nat Ecol Evol* **5**, 845–853 (2021).
110. Crippa, M. *et al.* Food systems are responsible for a third of global anthropogenic GHG emissions. *Nat Food* **2**, 198–209 (2021).
111. Feng, Y. *et al.* Doubling of annual forest carbon loss over the tropics during the early twenty-first century. *Nat Sustain* **5**, 444–451 (2022).
112. Sanderman, J., Hengl, T. & Fiske, G. J. Soil carbon debt of 12,000 years of human land use. *Proceedings of the National Academy of Sciences* **114**, 9575–9580 (2017).
113. Guo, L. B. & Gifford, R. M. Soil carbon stocks and land use change: a meta analysis. *Global Change Biology* **8**, 345–360 (2002).
114. Don, A., Schumacher, J. & Freibauer, A. Impact of tropical land-use change on soil organic carbon stocks – a meta-analysis. *Global Change Biology* **17**, 1658–1670 (2011).
115. Powers, J. S., Corre, M. D., Twine, T. E. & Veldkamp, E. Geographic bias of field observations of soil carbon stocks with tropical land-use changes precludes spatial extrapolation. *Proceedings of the National Academy of Sciences* **108**, 6318–6322 (2011).
116. Veldkamp, E., Schmidt, M., Powers, J. S. & Corre, M. D. Deforestation and reforestation impacts on soils in the tropics. *Nat Rev Earth Environ* **1**, 590–605 (2020).

117. Poeplau, C. *et al.* Temporal dynamics of soil organic carbon after land-use change in the temperate zone – carbon response functions as a model approach. *Global Change Biology* **17**, 2415–2427 (2011).
118. Dlamini, P., Chivenge, P. & Chaplot, V. Overgrazing decreases soil organic carbon stocks the most under dry climates and low soil pH: A meta-analysis shows. *Agriculture, Ecosystems & Environment* **221**, 258–269 (2016).
119. Wei, X., Shao, M., Gale, W. & Li, L. Global pattern of soil carbon losses due to the conversion of forests to agricultural land. *Sci Rep* **4**, 4062 (2014).
120. Beillouin, D. *et al.* A global meta-analysis of soil organic carbon in the Anthropocene. *Nat Commun* **14**, 3700 (2023).
121. Mathew, I., Shimelis, H., Mutema, M. & Chaplot, V. What crop type for atmospheric carbon sequestration: Results from a global data analysis. *Agriculture, Ecosystems & Environment* **243**, 34–46 (2017).
122. Wiesmeier, M. *et al.* Estimation of past and recent carbon input by crops into agricultural soils of southeast Germany. *European Journal of Agronomy* **61**, 10–23 (2014).
123. Durán Zuazo, V. H., Francia Martínez, J. R., Pleguezuelo, C. R. R. & Tavira, S. C. Biomass carbon stock in relation to different land uses in a semiarid environment. *Journal of Land Use Science* **9**, 474–486 (2014).
124. Kuyah, S. *et al.* Grain legumes and dryland cereals contribute to carbon sequestration in the drylands of Africa and South Asia. *Agriculture, Ecosystems & Environment* **355**, 108583 (2023).
125. K, R. & B, B. Potential of wastelands for carbon sequestration- A review. *Int. J. Chem. Stud.* **8**, 2873–2881 (2020).
126. Koga, N. *et al.* Estimating net primary production and annual plant carbon inputs, and modelling future changes in soil carbon stocks in arable farmlands of northern Japan. *Agriculture, Ecosystems & Environment* **144**, 51–60 (2011).
127. Guillaume, T. *et al.* Carbon costs and benefits of Indonesian rainforest conversion to plantations. *Nat Commun* **9**, 2388 (2018).
128. Bukoski, J. J. *et al.* Rates and drivers of aboveground carbon accumulation in global monoculture plantation forests. *Nat Commun* **13**, 4206 (2022).
129. Brakas, S. G. & Aune, J. B. Biomass and Carbon Accumulation in Land Use Systems of Claveria, the Philippines. in *Carbon Sequestration Potential of Agroforestry Systems: Opportunities and Challenges* (eds Kumar, B. M. & Nair, P. K. R.) 163–175 (Springer Netherlands, Dordrecht, 2011). doi:10.1007/978-94-007-1630-8_9.
130. Schafer, L. J., Lysák, M. & Henriksen, C. B. Tree layer carbon stock quantification in a temperate food forest: A peri-urban polyculture case study. *Urban Forestry & Urban Greening* **45**, 126466 (2019).
131. Zahoor, S. *et al.* Apple-based agroforestry systems for biomass production and carbon sequestration: implication for food security and climate change contemplates in temperate region of Northern Himalaya, India. *Agroforest Syst* **95**, 367–382 (2021).
132. Toensmeier, E. & Herren, H. *The Carbon Farming Solution: A Global Toolkit of Perennial Crops and Regenerative Agriculture Practices for Climate Change Mitigation and Food Security*. (Chelsea Green Publishing, White River Junction, Vermont, UNITED STATES, 2016).
133. Victor, A. D., Valery, N. N., Boris, N., Aimé, V. B. T. & Louis, Z. Carbon storage in cashew plantations in Central Africa: case of Cameroon. *Carbon Management* **12**, 25–35 (2021).
134. Morandé, J. A. *et al.* From berries to blocks: carbon stock quantification of a California vineyard. *Carbon Balance and Management* **12**, 5 (2017).
135. Sharma, S., Rana, V. S., Prasad, H., Lakra, J. & Sharma, U. Appraisal of Carbon Capture, Storage, and Utilization Through Fruit Crops. *Frontiers in Environmental Science* **9**, (2021).
136. Sahoo, U. K., Nath, A. J. & Lalnunpuii, K. Biomass estimation models, biomass storage and ecosystem carbon stock in sweet orange orchards: Implications for land use management. *Acta Ecologica Sinica* **41**, 57–63 (2021).
137. Lopez-Bellido, P. J., Lopez-Bellido, L., Fernandez-Garcia, P., Muñoz-Romero, V. & Lopez-Bellido, F. J. Assessment of carbon sequestration and the carbon footprint in olive groves in Southern Spain. *Carbon Management* **7**, 161–170 (2016).
138. Singh, K. P. *et al.* Biomass, carbon stock, CO2 mitigation and carbon credits of coffee-based multitier cropping model in Central India. *Environ Monit Assess* **195**, 1250 (2023).
139. Asigbaase, M., Dawoe, E., Lomax, B. H. & Sjogersten, S. Biomass and carbon stocks of organic and conventional cocoa agroforests, Ghana. *Agriculture, Ecosystems & Environment* **306**, 107192 (2021).
140. Das, M. *et al.* Biomass models for estimating carbon storage in *Areca* palm plantations. *Environmental and Sustainability Indicators* **10**, 100115 (2021).
141. Liang, X. *et al.* Quantifying shoot and root biomass production and soil carbon under perennial bioenergy grasses in a subtropical environment. *Biomass and Bioenergy* **128**, 105323 (2019).
142. Toensmeier, E., Ferguson, R. & Mehra, M. Perennial vegetables: A neglected resource for biodiversity, carbon sequestration, and nutrition. *PLOS ONE* **15**, e0234611 (2020).

143. Farina, R. *et al.* Potential carbon sequestration in a Mediterranean organic vegetable cropping system. A model approach for evaluating the effects of compost and Agro-ecological Service Crops (ASCs). *Agricultural Systems* **162**, 239–248 (2018).
144. FAO. *Statistical Standard Series: Observation Status Code List (Version 3)*. https://www.fao.org/3/cc6208en/cc6208en.pdf (2023).
