# Supplementary material for: Global patterns of commodity-driven deforestation and associated carbon emissions
Source: Nat Food. 2026 Feb 23;7(2):138–51. doi: 10.1038/s43016-026-01305-4 (PMC12935532; doi:10.1038/s43016-026-01305-4)
Supplement: Supplementary file 2 — Reporting Summary [file 43016_2026_1305_MOESM2_ESM.pdf]

Corresponding author(s): Chandrakant Singh

Last updated by author(s): Jan 12, 2026

# Reporting Summary

Nature Portfolio wishes to improve the reproducibility of the work that we publish. This form provides structure for consistency and transparency in reporting. For further information on Nature Portfolio policies, see our Editorial Policies and the Editorial Policy Checklist.

## Statistics

For all statistical analyses, confirm that the following items are present in the figure legend, table legend, main text, or Methods section.

| n/a | Confirmed | |
|---|---|---|
| ☐ | ☒ | The exact sample size (*n*) for each experimental group/condition, given as a discrete number and unit of measurement |
| ☒ | ☐ | A statement on whether measurements were taken from distinct samples or whether the same sample was measured repeatedly |
| ☒ | ☐ | The statistical test(s) used AND whether they are one- or two-sided<br>*Only common tests should be described solely by name; describe more complex techniques in the Methods section.* |
| ☒ | ☐ | A description of all covariates tested |
| ☒ | ☐ | A description of any assumptions or corrections, such as tests of normality and adjustment for multiple comparisons |
| ☐ | ☒ | A full description of the statistical parameters including central tendency (e.g. means) or other basic estimates (e.g. regression coefficient) AND variation (e.g. standard deviation) or associated estimates of uncertainty (e.g. confidence intervals) |
| ☒ | ☐ | For null hypothesis testing, the test statistic (e.g. *F*, *t*, *r*) with confidence intervals, effect sizes, degrees of freedom and *P* value noted<br>*Give P values as exact values whenever suitable.* |
| ☒ | ☐ | For Bayesian analysis, information on the choice of priors and Markov chain Monte Carlo settings |
| ☒ | ☐ | For hierarchical and complex designs, identification of the appropriate level for tests and full reporting of outcomes |
| ☒ | ☐ | Estimates of effect sizes (e.g. Cohen's *d*, Pearson's *r*), indicating how they were calculated |

*Our web collection on statistics for biologists contains articles on many of the points above.*

## Software and code

Policy information about availability of computer code

| | |
|---|---|
| Data collection | We utilised previously published remote sensing datasets and agricultural statistics (mentioned in Supplementary Table 2), and do not use any data collection software for our research. |
| Data analysis | Data analysis was conducted using Google Earth Engine and Python, with the codes archived on GitHub at: https://github.com/chandrakant6492/DeDuCE. Following are the packages and their respective versions used in this model: geetools: 1.4.0, rasterio: 1.4.0, matplotlib: 3.9.2, pandas: 2.2.3, tqdm: 4.66.5, numpy: 2.1.1, seaborn: 0.13.2, geopandas: 1.0.1, geemap: 0.34.5, plotly: 5.24.1, xarray: 2024.9.0. |

For manuscripts utilizing custom algorithms or software that are central to the research but not yet described in published literature, software must be made available to editors and reviewers. We strongly encourage code deposition in a community repository (e.g. GitHub). See the Nature Portfolio guidelines for submitting code & software for further information.

## Data

Policy information about availability of data

All manuscripts must include a data availability statement. This statement should provide the following information, where applicable:

- Accession codes, unique identifiers, or web links for publicly available datasets
- A description of any restrictions on data availability
- For clinical datasets or third party data, please ensure that the statement adheres to our policy

The deforestation and carbon emission estimates generated by the DeDuCE model, including those from sensitivity analyses, are available on Zenodo: https://doi.org/10.5281/zenodo.13624636. Trade analysis presented in Extended Fig.2 is available at: https://doi.org/10.5281/zenodo.10633818. All the datasets used in this study are documented in Supplementary Table 2. The insights from the DeDuCE model can be viewed at: https://www.deforestationfootprint.earth.

## Human research participants

Policy information about studies involving human research participants and Sex and Gender in Research.

| | |
|---|---|
| Reporting on sex and gender | N.A. |
| Population characteristics | N.A. |
| Recruitment | N.A. |
| Ethics oversight | N.A. |

Note that full information on the approval of the study protocol must also be provided in the manuscript.

# Field-specific reporting

Please select the one below that is the best fit for your research. If you are not sure, read the appropriate sections before making your selection.

☐ Life sciences ☐ Behavioural & social sciences ☒ Ecological, evolutionary & environmental sciences

For a reference copy of the document with all sections, see nature.com/documents/nr-reporting-summary-flat.pdf

# Ecological, evolutionary & environmental sciences study design

All studies must disclose on these points even when the disclosure is negative.

| | |
|---|---|
| Study description | The Deforestation Driver and Carbon Emission (DeDuCE) model leverages a comprehensive array of spatial and agricultural census data to quantify deforestation and the associated carbon emissions from agricultural and forestry activities. |
| Research sample | We employed the Global Forest Change dataset, that maps annual tree cover loss from 2001 to 2022 at 30-m resolution, to estimate deforestation linked to agriculture and forestry commodities. This estimation utilized datasets detailing the extent of specific crops (such as soybeans, oil palm, cocoa, and rubber), land uses (including croplands, forest plantations, and pastures), dominant drivers of deforestation, and the state of forest management. Additionally, when the explicit driver of deforestation was not apparent, we used agricultural statistics at the (sub-)national level for deforestation attribution.<br>Since this was a global-scale deforestation attribution of already defined tree cover loss pixels, there was no need for a research sample. |
| Sampling strategy | This study's findings cover 179 countries and 184 commodities, with results further aggregated into eight country groups and 11 commodity groups. Country groups were selected based on geographical location and specific deforestation patterns (particularly in Asia). Commodity groups were aggregated according to the FAO CPC classification (https://unstats.un.org/unsd/classifications/Family/Detail/1074).<br>This grouping was done solely for the purpose of reporting our results in the manuscript, and no additional sampling strategy was applied. |
| Data collection | We employed the best available datasets that have been published, quantifying commodities, land use/cover, and the dominant drivers of forest loss, with data available at least on a country scale. |
| Timing and spatial scale | The analysis was conducted annually from 2001 to 2022, with the final results aggregated at the national level (and at the sub-national level for Brazil). |
| Data exclusions | No data were excluded from this study. |
| Reproducibility | The utilization of Google Earth Engine and Python, combined with the use of openly available datasets, ensures the full |

| | |
|---|---|
| Reproducibility | reproducibility of this research. |
| Randomization | N.A. |
| Blinding | N.A. |

Did the study involve field work? ☐ Yes ☒ No

# Reporting for specific materials, systems and methods

We require information from authors about some types of materials, experimental systems and methods used in many studies. Here, indicate whether each material, system or method listed is relevant to your study. If you are not sure if a list item applies to your research, read the appropriate section before selecting a response.

## Materials & experimental systems

| n/a | Involved in the study |
|---|---|
| ☒ | ☐ Antibodies |
| ☒ | ☐ Eukaryotic cell lines |
| ☒ | ☐ Palaeontology and archaeology |
| ☒ | ☐ Animals and other organisms |
| ☒ | ☐ Clinical data |
| ☒ | ☐ Dual use research of concern |

## Methods

| n/a | Involved in the study |
|---|---|
| ☒ | ☐ ChIP-seq |
| ☒ | ☐ Flow cytometry |
| ☒ | ☐ MRI-based neuroimaging |
